# Novel Potent Imidazo[1,2-*a*]pyridine-*N*-Glycinyl-Hydrazone Inhibitors of TNF-α Production: *In Vitro* and *In Vivo* Studies

**DOI:** 10.1371/journal.pone.0091660

**Published:** 2014-03-14

**Authors:** Renata B. Lacerda, Natália M. Sales, Leandro L. da Silva, Roberta Tesch, Ana Luisa P. Miranda, Eliezer J. Barreiro, Patricia D. Fernandes, Carlos A. M. Fraga

**Affiliations:** 1 Laboratório de Avaliação e Síntese de Substâncias Bioativas (LASSBio), Universidade Federal do Rio de Janeiro, Rio de Janeiro, Rio de Janeiro, Brazil; 2 Programa de Pós-Graduação em Química, Instituto de Química, Universidade Federal do Rio de Janeiro, Rio de Janeiro, Rio de Janeiro, Brazil; 3 Programa de Pós-Graduação em Farmacologia e Química Medicinal, Instituto de Ciências Biomédicas, Universidade Federal do Rio de Janeiro, Rio de Janeiro, Rio de Janeiro, Brazil; 4 Laboratório de Estudos em Farmacologia Experimental, Faculdade de Farmácia, Universidade Federal do Rio de Janeiro, Rio de Janeiro, Rio de Janeiro, Brazil; 5 Laboratório de Farmacologia da Dor e da Inflamação, Instituto de Ciências Biomédicas, Universidade Federal do Rio de Janeiro, Rio de Janeiro, Rio de Janeiro, Brazil; Rutgers - New Jersey Medical School, United States of America

## Abstract

In this work, we describe the design, synthesis and pharmacological evaluation of novel imidazo[1,2-a]pyridine-N-glycinyl-hydrazone derivatives (1a–k) intended for use as inhibitors of tumor necrosis factor alpha (TNF-α) production. The compounds were designed based on the orally active anti-inflammatory prototype LASSBio-1504 (2), which decreases the levels of the pro-inflammatory cytokine TNF-α in vitro and in vivo. The in vitro pharmacological evaluation of the imidazo[1,2-a]pyridine compounds (1) showed that substitution of the N-phenylpyrazole core present in prototype 2 by a bioisosteric imidazo[1,2-a]pyridine scaffold generated anti-TNF-α compounds that were more potent than the previously described N-phenylpyrazole derivative 2 and as potent as SB-203580, a p38 MAPK inhibitor. The most active derivative (E)-2-(2-tert-butylimidazo[1,2-a]pyridin-3-ylamino)-N’-(4-chlorobenzylidene) acetohydrazide, or LASSBio-1749 (1i) was orally active as an anti-inflammatory agent in a subcutaneous air pouch model, reducing expressively the levels in vivo of TNF-α and other pro-inflammatory cytokines at all of the tested doses.

## Introduction

Tumor necrosis factor alpha (TNF-α) is a major pro-inflammatory cytokine that functions as a mediator of acute inflammation, platelet activation and participation in the genesis of fever and anemia. Increased production of this cytokine is associated with a number of autoimmune and inflammatory diseases, such as Crohn’s disease, psoriasis, diabetes [1], [2], multiple sclerosis [3], atherosclerosis and rheumatoid arthritis (RA) [4]. In these diseases, TNF-α modulates processes such as immune cell activation, proliferation, apoptosis and leukocyte migration. In RA, which is a chronic systemic inflammatory disease, TNF-α is involved in inflammation and in the mechanisms of cartilage and bone joint destruction. High levels of TNF-α have been detected in the synovial membranes of patients with acute and chronic RA [5].

The modulation of the biosynthesis of pro-inflammatory cytokines is an important strategy for the treatment of inflammatory diseases; the therapeutic potential of targeting TNF-α, for example, has been validated by the success of drugs such as infliximab and etanercept [6]. However, due to the various limitations of the chronic use of these drugs [7], such as the occurrence of serious side effects and high drug production costs, new small molecules capable of modulating the biosynthesis of TNF-α and other pleiotropic cytokines must be developed.

**Figure 1:**
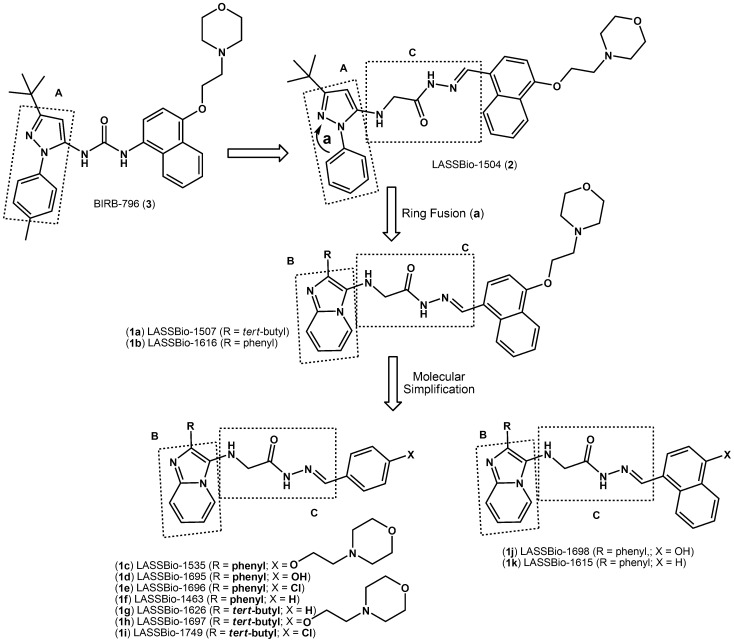
Design of the novel imidazo[1,2-*a*]pyridine-*N*-glycinyl-hydrazone derivatives 1a–k.

As part of a research program aimed at the identification of new anti-TNF-α lead compounds for the treatment of inflammatory diseases, our research group has recently described the discovery of LASSBio-1504 (2) [8], a structural analogue of BIRB-796 (3) (Figure 1) [9], [10] with in vitro and in vivo anti-TNF-α activity and in vivo anti-inflammatory and antinociceptive properties.

In a continuing effort to identify potent and safe anti-TNF-α inhibitors, we report herein the design, synthesis and anti-TNF-α evaluation of several novel imidazo[1,2-a]pyridine-N-glycinyl-hydrazone derivatives (1a–k), which were produced via the structural modification of prototype 2. For the proposed derivatives (1a–k), we replaced the N-phenyl-pyrazole nucleus (A, Figure 1) of LASSBio-1504 (2) with the isosteric heterocycle imidazo[1,2-a]pyridine (B, Figure 1). We then performed a series of molecular simplifications in the functionalized naphthyl framework attached to the imine unit of the N-acylhydrazone (NAH) groups of compounds 1a and 1b to better understand the structure-activity relationship (Figure 1).

## Results and Discussion

### Chemistry

**Figure 2:**
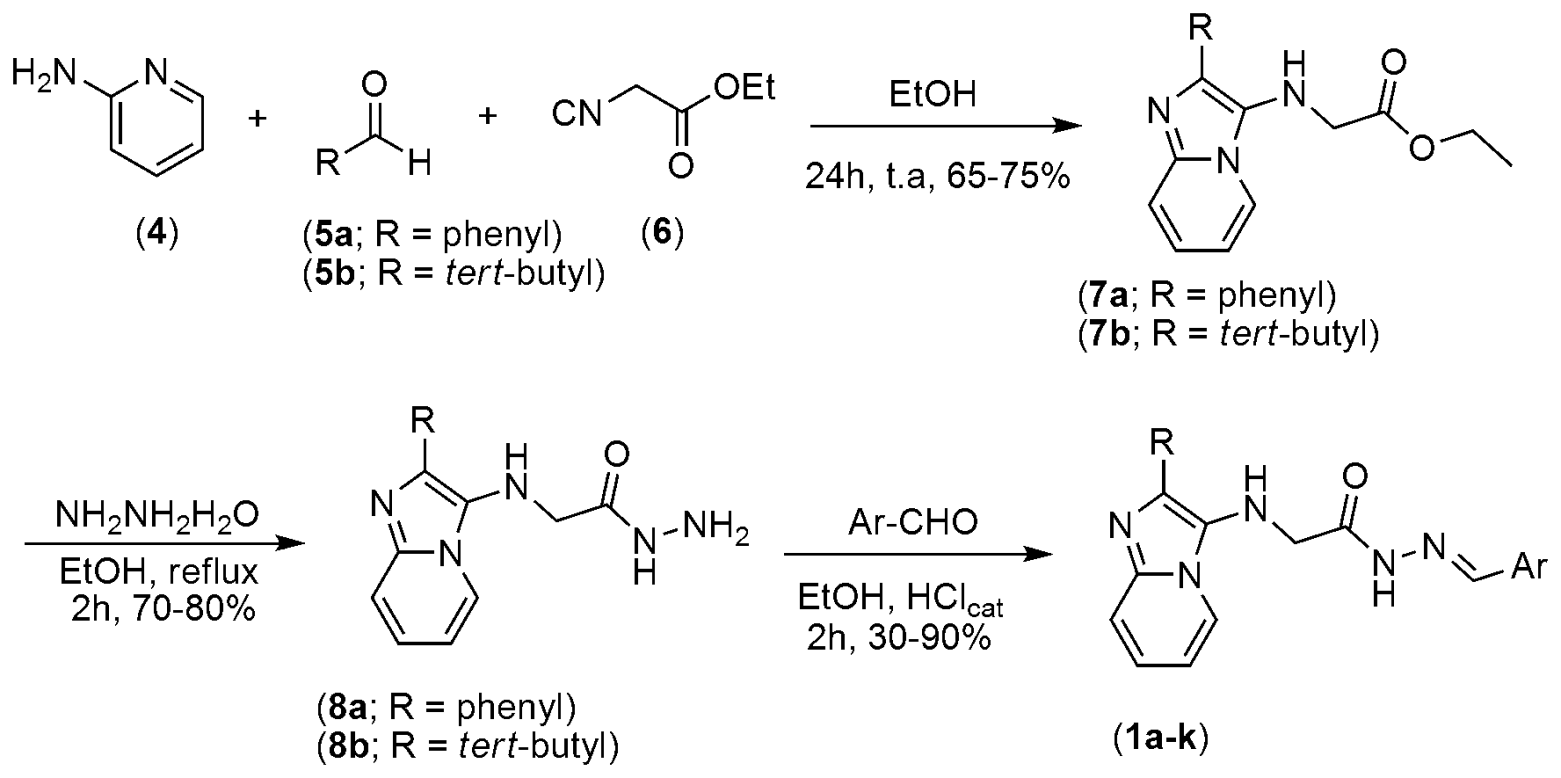
Synthesis of imidazo[1,2-*a*]-pyridine-*N*-glycinyl-hydrazone derivatives 1a–k.

To obtain the new imidazo[1,2-*a*]pyridine-*N*-glycinyl-hydrazone derivatives (1a–k), we performed multicomponent reactions (MCR) [11], [12] between 2-aminopyridine (4), the appropriate aldehyde (5a or 5b) and ethyl 2-isocyanoacetate (6) (an isonitrile) (Figure 2). MCRs represent rapid and efficient strategy for the generation of bioactive compounds [13], [14] because the number of possible products increases with an increase in the number of components. In addition, the proposed strategy is step economical compared to the condensation of 2-aminopyridine with α-haloketones and subsequent functionalization [15]–[18]. To prepare the 3-amino-imidazo[1,2-*a*]pyridine esters, we used the MCR developed by Groebke [19], which is a variation of the classical Ugi [12] four-component reaction. In the MCR proposed by Ugi, the fourth component is often a carboxylic acid. The MCR developed by Groebke occurs between an aminoazine, aldehyde and isonitrile to form 3-amine-substituted heterocycles in one pot. Thus, the first step for the production of imidazo[1,2-*a*]-pyridine-*N*-glycinyl-hydrazone derivatives 1a–k consisted of the preparation of imidazo[1,2-*a*]pyridine esters (7a–b) in 65–75% yield by an MCR in ethanol at room temperature (Figure 2). Next, hydrazinolysis of the methyl esters (7a–b) with hydrazine hydrate in ethanol under reflux produced the corresponding hydrazide intermediates (8a–b) in 70–80% yield. The novel imidazo[1,2-*a*]-pyridine-*N*-glycinyl-hydrazone derivatives 1a–k (Table 1) were prepared with satisfactory yields through the acid-catalyzed condensation of hydrazides (8a–b) with selected aromatic aldehydes at room temperature (Figure 2). The structures of imidazo[1,2-*a*]pyridine-*N*-glycinyl-hydrazones1a–k were fully characterized using common spectroscopic methods, and the analytical results for C, H and N were within ±0.4% of the calculated values. The purity of the imidazo[1,2-*a*]pyridine-*N*-glycinyl-hydrazones (1a–k) was greater than 97%, as determined by reversed-phase HPLC.

**Table 1:** Yields and physical properties of imidazo[1,2-*a*]pyridine-*N*-glycinyl-hydrazone derivatives 1a–k.

Compound	MolecularFormula^a^	MW	Yield (%)^b^	m.p. (°C)
**1a**	C_30_H_36_N_6_O_3_H_2_O	545.68	30	135–137
**1b**	C_32_H_32_N_6_O_3_H_2_O	566.65	85	105–108
**1c**	C_28_H_30_N_6_O_3_	498.58	60	169–171
**1d**	C_22_H_19_N_5_O_2_	385.42	90	273–275
**1e**	C_22_H_18_ClN_5_O	403.86	55	120–122
**1f**	C_22_H_19_N_5_O	369.42	70	138–141
**1g**	C_20_H_23_N_5_O	349.43	60	230–232
**1h**	C_26_H_34_N_6_O_3_	478.59	40	–c
**1i**	C_20_H_22_ClN_5_O	383.87	68	232–234
**1j**	C_20_H_22_N_5_OH_2_O	435.48	70	>300
**1k**	C_26_H_21_N_5_O	419.48	90	208–210

a The analytical results for C, H and N were within 0.4% of the calculated values for all *N*-acylhydrazone derivatives 1a–k. The purity of *N*-acylhydrazone derivatives 1a–k was also determined by reversed-phase HPLC.

b The yields refer to the condensation step of hydrazides 8a–b with the corresponding aromatic aldehydes (see Figure 2).

c Obtained as a yellow oil (see Material and Methods).

**Figure 3:**
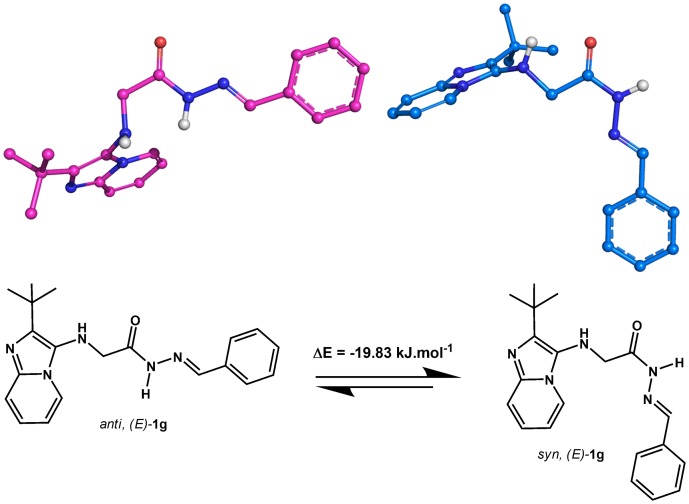
Probable conformational isomers of the NAH derivatives. Tridimensional representation of antiperiplanar (magenta) and synperiplanar (blue) conformers of LASSBio-1626 (1 g) identified in the Monte Carlo conformational search.

With respect to the C = N double bond, *N*-acylhydrazones (NAHs) may exist as *Z*/*E* geometrical isomers and syn/anti amide conformers [20], [21]. For most of the NAH derivatives described herein, the ^1^H-NMR spectra were recorded at room temperature, and two species were clearly detected. In contrast, only one species was observed by reversed-phase HPLC analysis (Figure S26 in File S1). In a study involving compounds 1f and 1 g, the ^1^H-NMR spectra obtained in DMSO-*d_6_* at 90°C showed that the two species were in rapid equilibrium (Figures S12–S13 and S15–S16 in File S1). Interestingly, a complete coalescence of the signals was achieved at 90°C, and the reversibility of the changes was verified, indicating the presence of conformational isomers [22], [23]. Indeed, a Monte Carlo conformational search performed on derivative 1 g followed by energy calculations of the selected conformers according to the Hartree-Fock 3–21G method indicated a slight difference in energy (ΔE =  −19.83 kJ.mol^-1^) between the synperiplanar and antiperiplanar conformers in the favor of the former (Figure 3). Therefore, we concluded that the novel derivatives 1 were obtained as single *E* geometrical isomers and that the observed duplication pattern was due to the presence of syn/anti amide conformers in DMSO (Figure 3).

### Pharmacology

Next, we investigated the ability of NAH derivatives 1a–k to inhibit TNF-α production *in vitro* [24]. The prototype LASSBio-1504 (2) and the p38 MAPK inhibitor SB-203580 (Figure S27 in File S1) were chosen as standards. As shown in Table 2, seven NAH derivatives, *i.e.*, 1a, 1b, 1d, 1e and 1i-k, inhibited the *in vitro* LPS-induced production of TNF-α in cultured mouse peritoneal macrophages at a concentration of 10 µM. Among the NAH derivatives, 1a (IC_50_ = 0.62 µM), 1b (IC_50_ = 0.34 µM), 1i (IC_50_ = 0.21 µM), 1j (IC_50_ = 0.31 µM) and 1 k (IC_50_ = 0.99 µM) showed the most potent inhibitory effects. Specifically, the inhibitory effects of 1a, 1b, 1i, 1j and 1k were superior to that of *N*-phenylpyrazole prototype 2 (IC_50_ = 3.6 µM) and similar to that of SB-203580 (IC_50_ = 0.22 µM). The in vitro anti-TNF-α inhibitory potency of *N*-acylhydrazone (NAH) derivatives (1) has increased with the addition of more lipophilic groups attached to the imine group of NAH moiety. This behavior could be seen through the comparison of the IC_50_ of compounds 1e and 1i that presented para-chlorophenyl group (cLog P = 4.3) with the corresponding NAH presenting an unsubstituted phenyl ring, *i.e.* 1f (cLog P = 3.7) and 1 g (cLog P = 3.8). Additionally, similar profile was evidenced when we compared the inhibitory potencies of compounds 1a and 1b, presenting 4-(2-(naphthalen-1-yloxy)ethyl)morpholine unit (cLog P = 4.2) with those present 4-(2-(phen-1-yloxy)ethyl)morpholine group, *i.e.* 1 h and 1c (cLog P = 3.2).

**Table 2:** Effects of imidazo[1,2-*a*]pyridine-*N*-glycinyl-hydrazone derivatives 1a–k on TNF-α production and cell viability in murine peritoneal macrophages.

Compound	TNF-α ^a^		% of cell viability ^b^		SelectivityIndex(SI)^ d^	*c*Log P ^e^
	% inhibition at 10 µM	IC_50_(µM)^c^	at 10 µM	IC_50_ (µM)^c^(0.1–100)		
**1a**	95.3*	0.62 (0.03–10)	20.1*	6.9	11.1	4.2
**1b**	77.3*	0.34 (0.03–30)	78.4	14.7	43.2	4.2
**1c**	18.3	–	100	–	–	3.2
**1d**	56.7*	3.67 (0.1–30)	100	–	–	3.3
**1e**	53.8*	3.05 (0.1–30)	100	–	–	4.3
**1f**	29.3	–	100	–	–	3.7
**1g**	5.6	–	100	–	–	3.8
**1h**	2.8	–	100	–	–	3.2
**1i**	78.0*	0.21 (0.03–30)	73.3	21.7	103.3	4.3
**1j**	90.8*	0.31 (0.03–10)	40.0*	10.4	33.5	4.3
**1k**	75.8*	0.99 (0.03–30)	83.2	23.5	23.7	4.7
**SB-203580**	90.0*	0.22 (0.03–10)	44.3*	9.7	44.0	–
**LASSBio-1504 (2)**	96.9*	3.60 (0.1–30)	61.5*	10.3	2.8	6.0

The results are expressed as the ^a^percent inhibition and ^b^percent cell viability compared to the vehicle (DMSO); n = 3 independent experiments performed in duplicate;

*p = 0.05 using student’s t test; ^c^IC_50_ values were determined using at least five concentrations, the range concentration are showed in parentheses; ^d^ SI = cytotoxicity IC_50_/anti-TNF-α IC_50_;^ e^ values calculated using ACDLABS software.

Because the novel *N*-acylhydrazone derivatives 1 were designed based on the p38α MAPK inhibitor BIRB-796 (3), some of them were evaluated for their *in vitro* capacity to inhibit p38α MAPK activity [25] at a concentration of 10 µM. Interestingly, as the prototype LASSBio-1504 (2) none of the new imidazo[1,2-*a*]pyridine-*N*-glycinyl-hydrazone derivatives (1) showed to the able to inhibit p38α activity (Table S1 in File S1).

The cytotoxicity of the compounds was also evaluated using an MTT assay (the reduction of (3-(4,5-dimethylthiazol-2-yl)-2,5-diphenyltetrazolium bromide, a yellow tetrazole, to form purple formazan by cellular mitochondrial enzymes) to test the macrophage viability [26]. The percentage of viable cells was determined by comparison to control conditions (100% cell viability). As shown in Table 2, few of the NAH compounds showed significant cytotoxicity at a concentration of 10 µM. Even derivatives that showed some degree of toxicity had higher IC_50_ values for macrophage toxicity than for TNF-α inhibition. The observed selectivity index (SI) comparing macrophage toxicity and TNF-α inhibition demonstrated that the novel *N*-imidazo[1,2-*a*]pyridine-*N*-glycinyl-hydrazone derivatives 1 may be safer than prototype 2. In particular, the derivative LASSBio-1749 (1i) presented an improved potency/safety balance compared with the p38 MAPK inhibitor SB-203580.

**Figure 4:**
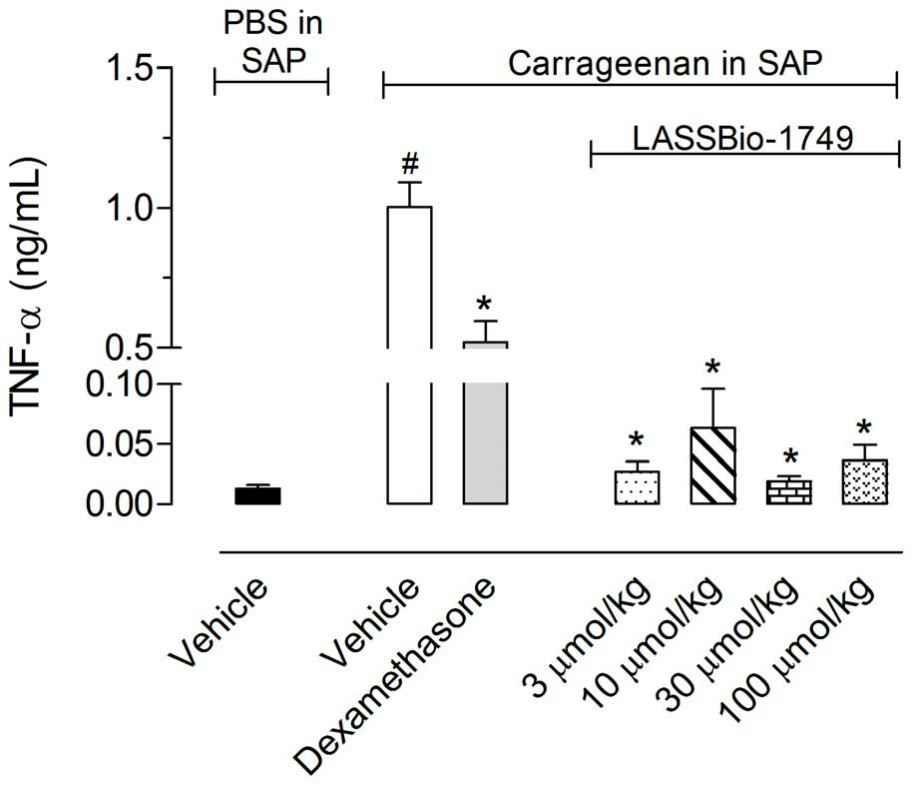
Effect of compound 1i on TNF-α production. Twenty-four hours prior to carrageenan (1%) injection into the subcutaneous air pouch (SAP), animals were pretreated by oral administration with different doses of a solution of 1i. The results are presented as the mean ± S. D. (n = 6–10) of TNF-α (pg/mL). Statistical significance was calculated by ANOVA followed by Bonferroni’s test. * indicates p<0.005 for the comparison of 1i -treated mice with the vehicle-treated group; # indicates p<0.005 for the comparison of vehicle-treated mice with the PBS-treated group.

Because compound 1i provided a better balance between the IC_50_ value for TNF-α production and cellular toxicity *in vitro*, we evaluated its effects in the subcutaneous air pouch (SAP) model, an *in vivo* model of inflammation and TNF production [27], [28]. In mice, oral pre-treatment with a solution of 1i significantly and dose dependently inhibited TNF-α production at all of the tested doses, and reductions greater than 90% were observed; *i.e.*, 96.8%, 90.6%, 97.3% and 95.31% for 3, 10, 30 and 100 µmol/kg doses, respectively (Figure 4).

**Figure 5:**
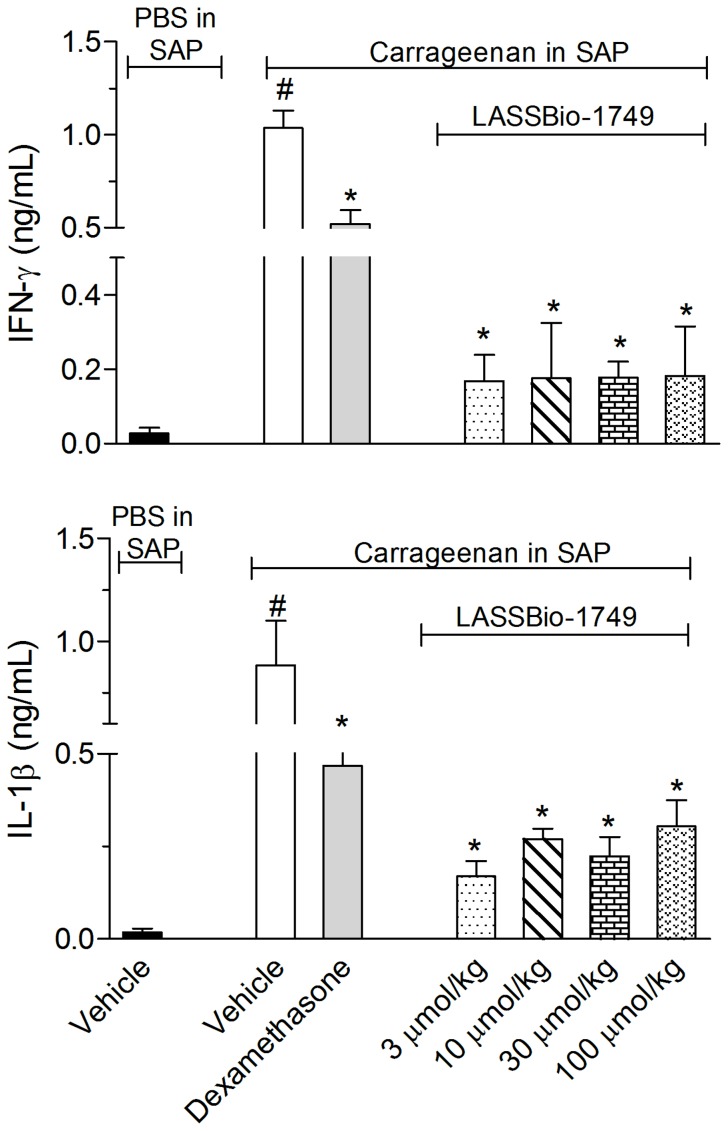
Effect of compound 1i on IL-1β and IFN-γ production. Twenty-four hours prior to carrageenan (1%) injection into the subcutaneous air pouch (SAP), animals were pretreated by oral administration with different doses of a solution of 1i. The results are presented as the mean ± S. D. (n = 6–10) of each cytokine (ng/mL). Statistical significance was calculated by ANOVA followed by Bonferroni’s test. * indicates p<0.005 for the comparison of 1i -treated mice with the vehicle-treated group; # indicates p<0.005 for the comparison of vehicle-treated mice with the PBS-treated group.

We also measured the levels of the cytokines interferon-γ (IFN- γ) and interleukin 1β (IL-1β). The derivative 1i also significantly inhibited both cytokines production in the SAP exsudate. The reduction in IL-1β vary between 65% and 81% (81%, 69.5%, 74.7% and 65.7% for 3, 10, 30 and 100 µmol/kg doses, respectively) and the inhibition of IFN- γ was higher than 80% (83.7%, 83%, 82.8% and 82.4% for 3, 10, 30 and 100 µmol/kg doses, respectively) (Figure 5).

## Conclusions

We described the design and synthesis of novel optimized NAH derivatives belonging to imidazo[1,2-a]pyridine-N-glycinyl-hydrazone series 1. These derivatives exhibited increased inhibitory effects on TNF-α production in vitro and in vivo due to the bioisosteric exchange of the N-phenylpyrazole ring present in prototype LASSBio-1504 (2) by the imidazo[1,2-a]pyridine nucleus. Our results indicated that differences in the hydrophobicity of the imine-attached framework played an important role in the in vitro anti-TNF-α activity of the imidazo[1,2-a]pyridine-N-glycinyl-hydrazone derivatives (1). LASSBio-1749 (1i) was determined to be the most active derivative of this series and was more potent than the prototype LASSBio-1504 (2) and equipotent to SB-203580 as an anti-TNF-α agent. Furthermore, the observed decrease in the cytotoxicity against macrophages indicated that the imidazo[1,2-a]pyridine-N-glycinyl-hydrazone derivatives are good candidates for safer anti-inflammatory drugs due to their potential low cytotoxicity profiles. The inhibition of the levels in vivo of IL-1β and IFN-γ indicated that LASSBio-1749 could act as a blocker of a transcription factor cascade responsible for the signaling the biosynthesis of these pro-inflammatory cytokines. Nowadays, we are currently investigating the efficacy of LASSBio-1749 (1i) in chronic inflammatory disease models, as the adjuvant-induced arthritis in rats, in order to confirm their therapeutic potential and advance in the preclinical pharmacological studies as a safer, more accessible and less costly alternative to the biotech drugs usually exploited to treat these diseases.

## Materials and Methods

### Chemistry

Reactions were routinely monitored by thin-layer chromatography (TLC) on silica gel (F245 Merck plates) and the products visualized with ultraviolet lamp (254 and 365 nm). NMR spectra were recorded on a 200/50 MHz Bruker DPX-200, 250/62.5 MHz Bruker DPX-250, 400/100 MHz Varian 400-Mr, 300/75 MHz Varian Unity-300 spectrometer at room temperature. Peak positions are given in parts per million (δ) from tetramethylsilane as internal standard, and coupling constant values (*J*) are given in Hz. Infrared (IR) spectra were obtained using a Nicolet Magna IR 760 spectrometer. Samples were examined as potassium bromide (KBr) disks. Elemental analyses were carried out on a Thermo Scientific Flash EA 1112 Series CHN-Analyzer. Melting points were determined using a Quimis instrument and are uncorrected. Column chromatography purifications were performed using silica gel Merck 230–400 mesh. All described products showed ^1^H and ^13^C NMR spectra according to the assigned structures. All organic solutions were dried over anhydrous sodium sulfate and all organic solvents were removed under reduced pressure in rotatory evaporator. HPLC for purity determinations were conducted using Shimadzu LC-20AD with a SHIM-PACK CLC-ODS analytical column (4.6 mm×250 mm) or Kromasil 100-5C18 (4.6 mm×250 mm) and a Shimadzu SPD-M20A detector at 254 nm wavelength. The solvent systems for HPLC purity analyses was acetonitrile:phosphate buffer solution pH 7 = 70∶30. The isocratic HPLC mode was used, and the flow rate was 1.0 mL/min.

#### General procedure for the multicomponent reaction (MCR) with isonitriles

Benzaldehyde or pivalaldehyde (1 mmol), 2-aminopyridine (1 mmol) and ethyl 2-isocyanoacetate (1 mmol) were dissolved or suspended in 3 mL EtOH. Glacial acetic acid (2 mmol) was added. The reaction mixture was stirred at room temperature overnight and then solvent was evaporated to dryness. The residue was taken up in aqueous KHCO_3_ and extracted with EtOAc (3×50 mL). Further purification was effected by column chromatography in dichloromethane.

#### Ethyl 2-(2-phenylimidazo[1,2-*a*]pyridin-3-ylamino)acetate (7a)

Yellow oil purified by silica gel chromatography after elution with dichloromethane, 65% yield; ^1^H NMR (200 MHz, DMSO-*d_6_*) δ = 8.47 (d,1H, *J* = 6.8 Hz, H5), 8.13 (d, 2H, *J* = 7.2 Hz, 2xCH), 7.44 (m, 3HAr), 7.31 (d, 1H, *J* = 7.2 Hz, H8), 7.23-7.20 (m, 1H, H7), 6.89 (t, 1H, *J* = 6.4 Hz, H6), 5.41 (t, 1H, *J* = 6.1 Hz, NH), 4.00 (q, 2H, *J* = 7.1 Hz, CH_2_), 3.80 (d, 2H, *J* = 6.1 Hz, CH_2_), 1.23 (t, 3H, *J* = 7.1 Hz, CH_3_); ^13^C NMR (50 MHz, DMSO-d_6_) δ = 171.6 (C = O), 140.4 (C8a), 134.4 (C1 phenyl), 133.2 (C2), 128,4 (C3 and C5 phenyl), 127.7 (C4 phenyl), 126.4 (C2 and C6 phenyl), 126.2 (C3), 123.9 (C5), 123.8 (C7), 116.5 (C8), 111.0 (C6), 60.3 (CH_2_), 48,7 (CH_2_), 13.8 (CH_3_); IR (KBr) cm^-1^ 3258 (NH), 2967 (CH), 1726 (C = O). Anal. Calcd for C_17_H_17_N_3_O_2_ (295.34): C 69.14; H 5.80; N 14.23. Found: C 68.97; H 5.82; N 14.19.

#### Ethyl 2-(2-*tert*-butylimidazo[1,2-*a*]pyridin-3-ylamino)acetate (7b)

Yellow oil purified by silica gel chromatography after elution with dichloromethane, 75% yield; ^1^H NMR (200 MHz, DMSO-*d_6_*) δ = 8.33 (d, 1H, *J* = 6.7 Hz; H5), 7.37 (d, 1H, *J* = 9.0 Hz, H8), 7.17-7.00 (m, 1H, H7), 6.81 (t, 1H, *J* = 6.7 Hz, H6), 4.59 (t, 1H, *J* = 6.1 Hz, NH), 4.12 (q, 2H, *J* = 7.1 Hz, CH_2_), 3.72 (d, 2H, *J* = 6.1 Hz, CH_2_), 1.37 (s, 9H, (CH_3_)_3_), 1.19 (s, 3H, *J* = 7.1 Hz, CH_3_); ^13^C NMR (50 MHz, DMSO-d_6_) δ = 171.7 (C = O), 145.8 (C2), 139.2(C3), 123.8 (C8a), 123.2 (C7), 123.0 (C5), 116.3(C8), 110.7 (C6), 60.3 (CH_2_), 50.3 (CH_2_), 32.7 (C(CH_3_)_3_), 30.3 (3xCH3), 14.0 (CH_3_); IR (KBr) cm^-1^ 3371 (NH), 2945 (CH), 1715 (C = O). Anal. Calcd for C_15_H_21_N_3_O_2_ (275.35): C 65.43; H 7.69; N 15.26. Found: C, 65.62; H, 7.71; N 15.22.

#### General procedure for preparation of hydrazides (8a,b)

A round-bottomed flask charged with 2 mmol of the respective ethyl ester (7a or 7b), 100% hydrazine hydrate (20 equiv.) and ethanol (5 mL) was stirred and heated at reflux for 2 hours. To the resulting mixture was added cold water and the precipitate formed was filtered out or the mixture was extracted with dichloromethane (3×50 mL) to give the corresponding hydrazides as described next.

#### 2-(2-Phenylimidazo[1,2-*a*]pyridin-3-ylamino)acetohydrazide (8a)

Yellow-green powder, 70% yield; ^1^H NMR (200 MHz, DMSO-*d_6_*) δ = 9.09 (NHC = O), 8.49 (d, 1H, *J* = 6.7 Hz, H5), 8.15 (d, 2H, *J* = 7.5 Hz, H2 and H6 phenyl), 7.44 (m, 3H, 3HAr), 7.31 (d, 1H, *J* = 7.0 Hz, H8), 7.17 (m, 1H, H7), 6.87 (t,1H, *J* = 6.7 Hz, H6), 5.19 (t, 1H, *J* = 6.0 Hz, NH), 4.22 (NH_2_), 3.55 (d, 2H, *J* = 6.0 Hz, CH_2_); ^13^C NMR (50 MHz, DMSO-d_6_) δ = 169.3 (C = O), 139.9 (C8a), 134.0 (C4 phenyl), 133.0 (C1 phenyl), 127.9 (C3 and C5 phenyl), 126.3 (C3), 126.2(C7), 125.9 (C2 and C6 phenyl), 123.6 (C2), 123.4 (C5), 116.0 (C8), 110.5 (C6), 48.5 (CH_2_); IR (KBr) cm^-1^ 3281 (NH), 1641 (C = O). Anal. Calcd for C_15_H_15_N_5_O (281.31): C 64.04; H 5.37; N 24.90. Found: C 64.21; H 5.36; N 24.87.

#### 2-(2-*Tert*-butylimidazo[1,2-*a*]pyridin-3-ylamino)acetohydrazide (8b)

Yellow oil, 80% yield; ^1^H NMR (200 MHz, DMSO-*d_6_*) δ = 9.16 (NHC = O), 8.28 (d, 1H, *J* = 6.8 Hz, H5), 7.37 (d, 1H, *J* = 8.9 Hz, H8), 7.17-7.02 (m, 1H, H7), 6.81 (t, 1H, *J* = 6.7 Hz, H6), 4.46 (t, 1H, *J* = 6.1 Hz, NH), 4.30 (NH_2_), 3.46 (d, 2H, *J* = 6.1 Hz, CH_2_), 1.38 (s, 9H, 3xCH_3_); ^13^C NMR (50 MHz, DMSO-d_6_) δ = 169.6 (C = O), 145.3 (C2), 139.1 (C3), 124.3 (C8a), 123.1 (C7), 122.9 (C5), 116.3 (C8), 110.8 (C6), 50.2 (CH_2_), 32.7 (C(CH_3_)_3_), 30.3 (3xCH_3_); IR (KBr) cm^-1^ 3388 (NH), 2963(CH), 1624(C = O). Anal. Calcd for C_13_H_19_N_5_O (261.32): C 59.75; H 7.33; N 26.80. Found: C 59.88; H 7.34; N 26.73.

#### General procedure for the preparation of imidazo[1,2-*a*]pyridine-*N*-glycinyl-*N*-acylhydrazones (1a–k)

In a round flask containing hydrazide derivative 8a or 8b (1.6 mmol) in ethanol (10 mL), was added the respective aromatic aldehyde (1.68 mmol; 1.05 equiv.) and catalytic concentrated hydrochloric acid. The mixture was stirred for about 2 hours at room temperature. At the end of the reaction, the solvent was removed under reduced pressure and a mixture of crushed ice and saturated sodium bicarbonate solution was added to the obtained residue. The precipitate formed was filtered out or the mixture was extracted with dichloromethane (3×50 mL) to furnish the title *N*-acylhydrazone compounds, as described next.

#### (*E*)-2-(2-*tert*-butylimidazo[1,2-*a*]pyridin-3-ylamino)-*N*’-((4-(2-morpholinoethoxy) naphthalen-1-yl)methylene)acetohydrazide (1a, LASSBio-1507)

Compound purified by silica gel chromatography after elution with dichloromethane/methanol (gradient) and recrystallized from acetone, 40% yield, m.p. = 135–137°C; ^1^H NMR (200 MHz, DMSO-*d_6_*) δ = 11.48 and 11.45 (2 s, 1H, NHC = O), 8.97 and 8.63 (2d, 1H, *J* = 8.0 Hz, HAr), 8.70 and 8.51 (2 s,1H, N = CH), 8.38 (d, 1H, *J* = 6.5 Hz, HAr), 8.24 (m, 1H, HAr), 7.82-7.56 (m, 3H, 3HAr), 7.40 (d, 1H, *J* = 8.9 Hz, HAr), 7.15-6.99 (m, 2H, 2HAr), 6.88–6.79 (m, 1H, HAr), 4.65 and 4.51 (2t, 1H, *J* = 6.0Hz, NH), 4.29 (-O-CH_2_-CH_2_-), 4.17 and 3.70 (2d, 2H, *J* = 6.0 Hz, CH_2_), 3.57 (2xCH_2_ morpholine), 2.85 (-O-CH_2_-CH_2_-), 2.52 (2xCH_2_ morpholine); 1.43 (s, (CH_3_)_3_) [Figure S1 in File S1]; ^13^C NMR (50 MHz, DMSO-*d_6_*) δ = 172.4 and 167.3 (C = O), 156.4 (C4’naphthyl), 148.3 (8a’naphthyl), 146.0 (C4a’naphthyl), 144.8 (N = CH), 139.7 (C2), 131.7 (C3), 130.7 (CH naphthyl), 130.0 (CH naphthyl), 128.4 (C1’naphthyl), 126.3 (CH naphthyl), 125.7 (CH naphthyl), 124.8 (C7), 123.76 (C8a), 122.8 (C5), 122.4 (CH), 116.9 (C8), 111.4 (CH naphthyl), 105.7 (C6), 66.3 (-O-CH_2_-), 66.2 (2CH_2_ morpholine), 56.9 (-N-CH_2_-), 53.4 (2CH_2_ morpholine), 49.5 (CH_2_), 32.9 (C(CH_3_)_3_), 30.4 (3CH_3_) [Figure S2 in File S1]; IR (KBr) cm^-1^ 3329 (NH), 2956 (CH), 1684 (C = O); Anal. Calcd for C_30_H_36_N_6_O_3_H_2_O (545.68): C 65.91; H 7.01; N 15.37. Found: C 65.93; H 6.99; N 15.32. % of purity >99% by HPLC.

#### (*E*)-*N*’-((4-(2-morpholinoethoxy)naphthalen-1-yl)methylene)-2-(2-phenylimidazo [1,2-*a*]pyridin-3-ylamino)acetohydrazide (1b, LASSBio-1616)

Yellow powder purified by silica gel chromatography after elution with dichloromethane/methanol (gradient) and recrystallized from ethanol, 85% yield, m.p. = 105–108°C; ^1^H NMR (200 MHz, DMSO-*d_6_*) δ = 11.69 and 11.42 (2 s, 1H, NHC = O), 9.10-8.88 (m, 2H, 2CH), 8.63 and 8.46 (2 s, 1H, N = CH), 8.51-8.36 (m, 1H, CH), 8.12 (m, 2H, 2CH), 7.91 (m, 2H, 2CH), 7.77-7.51 (m, 7H, 7CH), 7.07 (t, 1H, CH), 6.05 (NH), 4.68 (m, 2H), 4.30 (m, 2H), 3.90 (m, 4H), 3.72 (m, 4H); ^1^H NMR (200 MHz,CDCl_3_) δ = 10.51 and 10.31 (2 s, 1H, NHC = O), 8.34 (m, 1H, HAr), 8.20 and 7.91 (m, 5H, 4HAr and N = CH), 7.63-7.16 (m, 7H, 7HAr), 7.04 (m, 1H, HAr), 6.72-6.56 (m, 2H, 2HAr), 4.27 (m, 2H, CH_2_ spacer), 4.20 (m, 2H, -OCH_2_-), 3.65 (m, 4H, 2CH_2_ morpholine), 2.86 (m, 2H, -N-CH_2_-), 2.56 (m, 4H, 4H, 2CH_2_ morpholine) [Figures S3 and S4 in File S1]; ^13^C NMR (50 MHz, CDCl_3_) δ = 172.8 (C = O), 156.7 (C4 naphthyl), 145.6 (C8a), 141.7 (N = CH); 135.7 (C4a naphthyl), 134.1 (C1a naphthyl), 131.6 (C1 phenyl), 129.8 (C3 and C5 phenyl), 129.0 (C4 phenyl), 128.8 (C3), 128.1 (C1 naphthyl), 127.8 (C2), 127.6 (CH naphthyl), 127.1 (C2 and C6 phenyl), 125.8 (CH naphthyl), 124.3 (C7), 123.8 (CH naphthyl), 122.9 (CH naphthyl), 121.5 (C5), 117.4 (C8), 112.0 (CH naphthyl), 104.3 (C6), 67.1 (2CH_2_ morpholine), 66.8 (-O-CH_2_-), 57.6 (-CH_2_-N-), 54.2 (2CH_2_ morpholine), 49.0 (CH_2_); IR (KBr) cm^-1^ 3393 (NH), 1674 (C = O) [Figure S5 in File S1]; Anal. Calcd. for C_32_H_32_N_6_O_3_H_2_O (566.65): C 67.83; H 6.05; N 14.83. Found: C 68.09; H 6.04; N 14.77. % of purity >99% by HPLC.

#### (*E*)-*N*’-(4-(2-morpholinoethoxy)benzylidene)-2-(2-phenylimidazo[1,2-*a*]pyridin-3-ylamino)acetohydrazide (1c, LASSBio-1535)

Yellow powder purified by silica gel chromatography after elution with dichloromethane/methanol (gradient), 60% yield, m.p. = 167–169°C; ^1^H NMR (200 MHz, DMSO-*d_6_*) δ = 11.27 and 11.23 (2 s, 1H, NHC = O), 8.54 and 8.45 (2d, 1H, *J* = 6.7 Hz, H5), 8.15 (d, 2H, *J* = 7.7 Hz, 2HAr), 7.98 and 7.85 (2 s, 1H, N = CH), 7.57 (d, 1H, *J* = 8.5 Hz, ArH), 7.48–7.38 (m, 4H, 4HAr), 7.33-7.25 (m, 1H, HAr), 7.18 (t, 1H, *J* = 7.7 Hz, H7), 6.99-6.87 (m, 3H, 3HAr), 5.31 and 5.15 (2t, 1H, *J* = 5.7 Hz, NH), 4.09 (m, 4H, -OCH_2_- and CH_2_ spacer), 3.74 (m, 4H, 2CH_2_ morpholine), 2.66 (m, 2H, -N-CH_2_-), 2.44 (m, 4H, 2CH_2_ morpholine) [Figure S6 in File S1]; ^13^C NMR (50 MHz, DMSO-*d_6_*) δ = 171.9 and 167.1 (C = O), 160.1 (C4 phenyl), 159.9 (C8a), 146.9 (C1 phenyl), 143.6 (N = CH), 140.5 (C4 phenyl), 134.6 (C3), 133.3 (C1 phenyl), 128.7 (C2, C6, C3e C5 phenyl), 128.5 (C2), 126.5 (C2, C6, C3 and C5 phenyl), 124.1 (C7), 116.3 (C5), 114.9 (C8), 111.2 (C6), 66.2 (2CH_2_), 65.5 (-O-CH_2_-), 56.9 (-N-CH_2_-), 53.6 (2CH_2_), 49.6 and 47.9 (CH_2_); IR (KBr) cm^-1^ 3331 (NH), 1669 (C = O) [Figure S7 in File S1]; Anal. Calcd. for C_28_H_30_N_6_O_3_ (498.58): C 67.45; H 6.06; N 16.86. Found: C 67.29; H 6.06; N 16.91. % of purity >99% by HPLC.

#### (*E*)-*N*’-(4-hydroxybenzylidene)-2-(2-phenylimidazo[1,2-*a*]pyridin-3-ylamino) acetohydrazide (1d, LASSBio-1695)

White powder recrystallized from n-hexane/ethyl acetate, 87% yield, m.p. = 273–275°C; ^1^H NMR (200 MHz, DMSO-*d_6_*) δ = 11.26 and 11.18 (2s, 1H, NHC = O), 9.95 and 9.89 (2s, 1H, OH), 8.55 and 8.45 (2d, 1H, *J* = 6.7 Hz, H5), 8.17 (d, 2H, *J* = 7.7 Hz, 2HAr), 7.96 and 7.82 (2 s, 1H, N = CH), 7.49-7.13 (m, 7H, 7HAr), 6.88-6.71 (m, 3H, 3HAr), 5.33 and 5.14 (2t, 1H, *J* = 5.7 Hz, NH), 4.13 and 3.72 (2d, 2H, *J* = 5.7 Hz, CH_2_) [Figure S8 in File S1]; ^13^C NMR (50 MHz, DMSO-*d_6_*) δ = 171.7 and 166.9 (C = O), 159.4 (C4 phenyl), 159.1 (C1 phenyl), 147.2 (C8a), 143.9 (N = CH), 140.4 (C4 phenyl), 134.5 (C1 phenyl), 133.2 (C2), 128.8 (C2 and C6 phenyl), 128.4 (C3 and C5 phenyl), 126.7 (C3), 126.4 (C2 and C6 phenyl), 124.9 (C7), 123.8 (C5), 116.5 (C8), 115.6 (C3 and C5 phenyl), 111.0 (C6), 49.6 and 47.8 (CH2) [Figure S9 in File S1]; IR (KBr) cm^-1^ 3340 (NH), 1670 (C = O), 1605 (CN), 1274 (C-OH); Anal. Calcd. for C_22_H_19_N_5_O_2_ (385.42): C 68.56; H 4.97; N 18.17. Found: C 68.67; H 4.96; N 18.13. % of purity >99% by HPLC.

#### (*E*)-*N*’-(4-chlorobenzylidene)-2-(2-phenylimidazo[1,2-*a*]pyridin-3-ylamino) acetohydrazide (1e, LASSBio-1696)

White powder recrystallized from ethanol, 54% yield, m.p. = 122–124°C; ^1^H NMR (200 MHz, DMSO-*d_6_*) δ = 11.47 (s, 1H, NHC = O), 8.55 and 8.44 (2d, 1H, *J* = 6.8 Hz, H5), 8.16 (d, 2H, *J* = 7.4 Hz, 2HAr), 8.06 and 7.89 (2s, 1H, N = CH), 7.66 (d, 1H, *J* = 8.3 Hz, HAr), 7.47-7.25 (m, 7H, 7HAr), 7.17 (t, 1H, *J* = 7.6 Hz, H7), 6.87 (t, 1H, *J* = 6.8 Hz, H6), 5.35 and 5.18 (2t, 1H, *J* = 5.7 Hz, NH), 4.16 and 3.76 (2d, 2H, *J* = 5.7 Hz, CH_2_) [Figure S10 in File S1]; ^13^C NMR (50 MHz, DMSO-*d_6_*) δ = 172.2 and 167.4 (C = O), 145.5 (C8a), 142.3 (N = CH), 140.4 (C4 phenyl), 134.6 (C1 phenyl), 134.2 (C1 phenyl), 133.3 (C4 phenyl), 132.8 (C2), 128.7 (C3 and C5 phenyl), 128.4 (C3 and C5 phenyl), 126.8 (C3), 126.7 (C7), 126.4 (C2 and C6 phenyl), 123.8 (C5), 116.6 (C8), 111.1 (C6), 47.8 (CH_2_); IR (KBr) cm^-1^ 3346 (NH), 1669 (C = O) [Figure S11 in File S1]; Anal. Calcd. for C_22_H_18_ClN_5_O (403.86): C 65.43; H 4.49; N 17.34. Found: C 65.62; H 4.49; N 17.29. % of purity >99% by HPLC.

#### (*E*)-*N*’-benzylidene-2-(2-phenylimidazo[1,2-*a*]pyridin-3-ylamino)acetohydrazide (1f, LASSBio-1463)

White powder, 69% yield, m.p. = 138–141°C; ^1^H NMR (200 MHz, DMSO-*d_6_*) δ = 11.73 and 11.49 (2s, 1H, NHC = O), 8.59 and 8.50 (2d, 1H, *J* = 6.9 Hz, H5), 8.15-8.13 (m, 2H, 2HAr), 8.09 and 7.92 (2s, N = CH), 7.65-7.22 (m, 10H, 10HAr), 6.95 (t, 1H, *J* = 6.9 Hz, H6), 5.37 and 5.23 (2t; 1H, *J* = 5.9 Hz, NH), 417 and 3.75 (2d, 2H, *J* = 5.9 Hz, CH_2_) [Figure S12 in File S1]; ^13^C NMR (50 MHz, DMSO-*d_6_*) δ = 171.6 and 166.9 (C = O), 146.8 (C8a), 143.7 (N = CH), 137.0 (C2), 133.8 (C3), 131.1 (C1 phenyl), 129.8 (C1 phenyl), 129.0 (C3 and C5 phenyl), 128.7 (C3 and C5 phenyl), 128.0 (C4 phenyl), 127.7 (C7), 126.9 (C2 and C6 phenyl), 126.8 (C5), 126.6 (C2 and C6 phenyl), 125.8 (C5), 115.2 (C8), 112.4 (C6), 47.3 and 40.8 (CH_2_) [Figure S14 in File S1]; IR (KBr) cm^-1^ 3338 (NH), 1687 (C = O); Anal. Calcd. for C_22_H_19_N_5_O (369.42): C 71.53; H 5.18; N 18.96. Found: C, 71.69; H, 5.16; N, 19.01. % of purity >99% by HPLC.

#### (*E*)-*N*’-benzylidene-2-(2-*tert*-butylimidazo[1,2-*a*]pyridin-3-ylamino)acetohydrazide (1g, LASSBio-1626)

Recrystallized from acetone, 60% yield, m.p. = 230–232°C; ^1^H NMR (200 MHz, DMSO-*d_6_*) δ = 11.51 and 11.44 (2 s, 1H, NHC = O), 8.36 and 8.28 (2d, 1H, *J* = 6.8 Hz, H5), 8.22 and 7.99 (2 s, 1H, N = CH), 7.99-7.22 (m, 6H, 6HAr), 7.12 (t, 1H, *J* = 7,8 Hz, H7), 6.82 (t, 1H, *J* = 6.7 Hz, H6), 4.58 and 4.13 (2t, 1H, *J* = 6.1 Hz, NH), 4.08 and 3.66 (2d, 2H, *J* = 6,1 Hz, CH_2_), 1.40 (s, 9H, 3x(CH_3_)) [Figure S15 in File S1]; ^13^C NMR (50 MHz, DMSO-*d_6_*) δ = 171.8 and 167.1 (C = O), 146.9 (C2), 144.3 and 143.8 (N = CH); 138.8 (C1 phenyl), 134.1 and 133.9 (C2), 130.0 (C3), 128.7 (C3 and C5 phenyl), 127.0 (C2 and C6 phenyl), 124.4 (C8a), 123.7 (C7), 123.3 (C5), 115.9 (C8), 111.3 (C6), 50.8 and 48.9 (CH_2_), 32.6 (C(CH_3_)_3_) 30.2 (3CH_3_) [Figure S17 in File S1]; IR (KBr) cm^-1^ 3343 (NH), 1678 (C = O); Anal. Calcd. for C_20_H_23_N_5_O (349.43): C 68.74; H 6.63; N 20.04. Found: C 68.57; H 6.65; N 19.97. % of purity >99% by HPLC.

#### (*E*)-2-(2-*tert*-butylimidazo[1,2-*a*]pyridin-3-ylamino)-*N*’-(4-(2-morpholinoethoxy) benzylidene)acetohydrazide (1 h, LASSBio-1697)

Yellow oil purified by silica gel chromatography after elution with dichloromethane/methanol (gradient), 40% yield; ^1^H NMR (200 MHz, DMSO-*d_6_*) δ = 11.40 and 11.36 (2 s, 1H, NHC = O), 8.35 and 8.27 (2d,1H, *J* = 6.9 Hz, H5), 8.13 and 7.92 (2s, N = CH), 7.63 (d, 1H, *J* = 8.2 Hz, H8), 7.50-7.37 (m, 2HAr, H3 and H5 phenyl), 7.12 (t, 1H, *J* = 7.5 Hz, H7), 7.02-6.83 (m, 3HAr, H2 and H6 phenyl, H6 pyridine), 4.56 and 4.42 (2s, 1H, NH), 4.06 (m, 2H, O-CH_2_-), 3.80-3.40 (CH_2_ spacer and 2CH_2_ morpholine), 2.66 (m, 2H, -N-CH_2_-), 2,22 (2CH_2_ morpholine), 1.43((CH_3_)_3_) [Figure S18 in File S1]; ^13^C NMR (50 MHz, DMSO-*d_6_*) δ = 172.4 and 167.4 (C = O), 160.4 (C4 phenyl), 145.6 (C2), 144.4 (N = CH), 139.8 (C3), 129.3 (C1 phenyl), 128.9 (C3 and C5 phenyl), 127.1 (C8a), 124.9 (C7), 123.7 (C5), 116.8 (C8), 115.4 (C2 and C6 phenyl), 111.6 (C6), 66.7 (-O-CH_2_-), 66.0 (2CH_2_ morpholine), 57.4 (-N-CH_2_-), 54.1 (2CH_2_ morpholine), 49.4 (CH_2_), 33.2 (C(CH_3_)_ 3_), 30.8 (3CH_3_) [Figure S19 in File S1]; IR (KBr) cm^-1^ 3485 (NH), 2953 (CH), 1671 (C = O); Anal. Calcd. for C_26_H_34_N_6_O_3_ (478.59): C 65.25; H 7.16; N 17.56. Found: C 65.13; H 7.17; N 17.60. % of purity >99% by HPLC.

#### (*E*)-2-(2-*tert*-butylimidazo[1,2-*a*]pyridin-3-ylamino)-*N*’-(4-chlorobenzylidene) acetohydrazide (1i, LASSBio-1749)

Recrystallized from acetone, 68% yield, m.p. = 232–234°C; ^1^H NMR (200 MHz, DMSO-*d_6_*) δ = 11.63 and 11.55 (2s, 1H, NHC = O), 8.36 and 8.27 (2d, 1H, *J* = 6.7 Hz, H5), 8.21 and 7.97 (2 s, 1H, N = CH), 7.73 (d, 1H, *J* = 8.5 Hz, H8), 7.60-7.37 (m, 4H, 4HAr), 7.10 (t, 1H, *J* = 8.0 Hz, H7), 6.81 (t, 1H, *J* = 6.7 Hz, H6), 4.61 and 4.44 (2t, 1H, *J* = 6.1 Hz, NH), 4.08 and 3.67 (2d, 2H, *J* = 6.0 Hz, CH_2_), 1.39 (s, 9H, 3xCH_3_) [Figure S20 in File S1]; ^13^C NMR (50 MHz, DMSO-*d_6_*) δ = 171.9 and 167.2 (C = O), 145.6 (C2), 145.3 and 142.5 (N = CH), 139.1 (C4 phenyl), 134.5 and 134.2 (C1phenyl), 133.1 and 132.8 (C3), 128.8 (C3 and C5 phenyl), 128.4 (C2 and C6 phenyl), 124.2 (C7), 123.1 (C8a), 122.8 (C5), 116.3 (C8), 110.8 (C6), 50.8 and 48.8 (CH_2_), 32.6 (C(CH_3_)_3_), 30.2 (3CH_3_) [Figure S21 in File S1]; IR (KBr) cm^-1^ 3348 (NH), 1682 (C = O); Anal. Calcd. for C_20_H_22_ClN_5_OH_2_O (383.87): C 59.77; H 6.02; N 17.43. Found: C 60.01; H 6.03; N 17.37. % of purity >99% by HPLC.

#### (*E*)-*N*’-((4-hydroxynaphthalen-1-yl)methylene)-2-(2-phenylimidazo[1,2-*a*] pyridin-3-ylamino)acetohydrazide (1j, LASSBio-1698)

White powder, 67% yield, m.p. >300°C; ^1^H NMR (200 MHz, DMSO-*d_6_*) δ = 11.26 (s, 1H, NHC = O), 10.76 (OH), 8.86 (d, 1H, *J* = 8.5 Hz, CH), 8.60-8.49 (m, 2H, 2HAr), 8.40 (s, 1H, N = CH), 8.20 (m, 3H, 3xCH), 7.70-7.13 (m, 8H, 8xHAr), 6.94-6.84 (m, 2H, 2HAr), 5.38 and 5.26 (2t, 1H, *J* = 5.7 Hz, NH), 4.24 and 3.77 (2d, 2H, *J* = 5.7 Hz, CH_2_) [Figure S22 in File S1]; ^13^C NMR (50 MHz, DMSO-*d_6_*) δ = 171.7 and 166.9 (C = O), 155.5 (C4 naphthyl), 147.9 (N = CH), 140.3 (C1 phenyl), 132.8 (C1a phenyl), 131.5 (C3), 130.5 (CH naphthyl), 129.8 (CH naphthyl), 128.4 (C3 and C5 phenyl), 127.5 (C1 naphthyl), 126.4 (C4a naphthyl), 125.0 (CH naphtyl), 124.1 (C7), 123.7 (C5), 122.7 (CH naphthyl), 120.1 (CH naphthyl), 110.9 (C8), 107.9 (C6), 49.7 and 48.3 (CH_2_) [Figure S23 in File S1]; IR (KBr) cm-1 3304 (NH), 1667 (C = O), 1574 (C-N), 1277 (C-OH); Anal. Calcd. for C_26_H_21_N_5_O_2_ (435.48): C 71.71; H 4.86; N 16.08. Found: C 71.59; H 4.87; N 16.11. % of purity >99% by HPLC.

#### (*E*)-N’-(naphthalen-1-ylmethylene)-2-(2-phenylimidazo[1,2-*a*]pyridin-3-ylami- no)acetohydrazide (1k, LASSBio-1615)

Yellow powder, 90% yield, m.p. = 208–210°C; ^1^H NMR (200 MHz, DMSO-*d_6_*) δ = 11.51 and 11.46 (2s, 1H, NHC = O), 8.77-8.70 and 8.61-8.54 (2m, 2H, HAr and N = CH), 8.45 (d, 1H, *J* = 7.7 Hz, HAr), 8.18 (d, 2H, *J* = 7.2 Hz, 2HAr), 8.06-7.82 (m, 3H, 3HAr), 7.68-7.44 (m, 6H, 6HAr), 7.34-7.15 (m, 2H, 2HAr), 6.87-6.96 (m, 1H, HAr), 5.41 and 5.30 (2t, 1H, *J* = 5.7 Hz, NH), 4.26 and 3.81 (2d, 2H, *J* = 5.7 Hz, CH_2_) [Figure S24 in File S1]; ^13^C NMR (50 MHz, DMSO-*d_6_*) δ = 172.8 and 167.9 (C = O), 146.9 (C8a), 143.6 (N = CH), 140.3 (C1 naphtyl), 134.4 (C1 phenyl), 133.5 (C2), 132.7 (C4a naphthyl), 130.4 (C4 phenyl), 130.0 (C naphthyl), 129.4 (C naphthyl), 129.2 (C naphthyl), 129.0 (C naphthyl), 128.9 (C naphthyl), 128.6 (C2, C6, C3 and C5 phenyl), 127.3 (C naphthyl), 127.1 (C naphthyl), 126.5 (C3), 125.6 (C naphthyl), 124.3 (C7), 123.7 (C5), 116.5 (C8), 111.3 (C6), 50.1 and 48.7 (CH_2_) [Figure S25 in File S1]; IR (KBr) cm^-1^ 3340 (NH), 1671 (C = O); Anal. Calcd. for C_26_H_21_N_5_O (419.48): C 74.44; H 5.05; N 16.70. Found: C, 74.34; H 5.07; N 16.65. % of purity >99% by HPLC.

### Pharmacology

#### Ethics statements

BALBc mice were obtained from the LASSBio breeding unit (Faculty of Pharmacy, UFRJ, Brazil) or from Instituto Vital Brazil (Niteroi, Brazil). All animals were kept under standard conditions, maintained in a 12-h light/dark cycle with water and food ad libitum until use. Animal experiments were performed according to the “Principles of Laboratory Animal Care and Use in Research”. All the animal procedures were approved by Ethics Committee on Animal Use of the Center of Health Sciences at Federal University of Rio de Janeiro (Comissão de Ética no Uso de Animais do Centro de Ciências da Saúde da Universidade Federal do Rio de Janeiro, CEUA-CCS-UFRJ) under the protocol numbers Farmácia 01 (ALPM) and DFBCICB-015 (PDF).

#### LPS-induced TNF-α production in culture of mice peritoneal macrophage

BALBc mice received thyoglicollate 3% (1 mL/mice; i.p.) and 3 days later the peritoneal cavities were washed with RPMI 1640 culture medium (Sigma, USA, supplemented with 10% fetal bovine serum). The peritoneal macrophages were plated onto 96-wells plate (30.000 cells/well) for 1 hour at 37°C in a humidified 5% CO_2_ atmosphere. Then, macrophages were incubated with the vehicle or compounds and 1 hour later stimulated with LPS(100 ng/mL) for 24 hours when the supernatants were collected to evaluate TNF-α production by ELISA kit (B&D Bioscience, USA).

#### Cell viability by MTT assay

The peritoneal macrophage were obtained and plated as described above. The cells were incubated with the vehicle or compounds for 20 hours when was added 200 µL of RPMI medium containing 0.5 mg/mL MTT 20 mL of MTT (3-(4,5-dimethylthiazol-2-yl)-2,5-diphenyltetrazolium bromide) followed by 4 hour of incubation at 37°C in a 5% CO_2_ atmosphere. After incubation supernatants were discarded and 200 µL DMSO (dimethylsulfoxide) were added to solubilize the MTT-formazan crystals. The optical density was measured in a microplate reader at 490 nm. The control groups consisted of cells with medium plus vehicle used to dissolve the substances and was considered as 100% of viable cells. Results are expressed as percentage of viable cells when compared with control groups.

#### Subcutaneous air pouch (SAP) model

The experimental protocol was similar to that described by Romano and colleagues (1997) [26] with several modifications described by Raymundo and colleagues (2011) [27]. The animals received a dorsal subcutaneous injection of sterile air (10 mL) on three alternate days to induce the SAP. On the sixth day, animals received a subcutaneous injection of sterile carrageenan suspension (1%; 1 mL). Mice were pre-treated with vehicle (Polysorbate 80), 1i (3, 10, 30 and 100 µmol/kg) or dexamethasone (1.5 µmol/kg, i.p.) 1 h before carrageenan injection into the SAP. The control group received an injection of sterile PBS (1 mL) into the SAP. Animals were sacrificed 24 h after carrageenan injection. The cavity was washed with 1 mL of sterile PBS. Exudates were collected, centrifuged at 11,000 rpm for 10 min at 4°C and TNF-α accumulated was quantified. The cytokines quantification was done by enzyme-linked immunosorbent assay (ELISA) according to the manufacturer’s instructions (B&D, USA). The results are expressed as pg/mL of each cytokine. Animals were sacrificed after an exposition to a saturated carbon dioxide atmosphere in an appropriate gas chamber.

#### Statistical analysis

Each experimental group was composed by 6–8 mice. Data obtained from experiments were expressed as mean ± S.E.M., compared with vehicle control and statistically analyzed by the Student’s t test. p<0.05 was considered significant. When appropriate, the IC_50_ values (i.e. the concentration able to inhibit 50% of the maximum effect observed) and the ED_50_ dose (i.e. the dose able to inhibit 50% the effect in vivo) were determined by non-linear regression using GraphPad Prism software v. 5.0.

## Supporting Information

File S1 **Figures S1–S26 and Table S1.** Figure S1. ^1^H NMR spectrum of 1a (LASSBio-1507) (DMSO-*d_6_*, 200 MHz). Figure S2.^ 13^C NMR spectrum of 1a (LASSBio-1507) (DMSO-*d_6_*, 50 MHz). Figure S3. ^1^H NMR spectrum of 1b (LASSBio-1616) (DMSO-*d_6_*, 200 MHz). Figure S4. ^1^H NMR spectrum of 1b (LASSBio-1616) (CDCl_3_, 200 MHz). Figure S5. ^13^C NMR spectrum of 1b (LASSBio-1616) (CDCl_3_, 50 MHz). Figure S6. ^1^H NMR spectrum of 1c (LASSBio-1535) (DMSO-*d_6_*, 200 MHz). Figure S7. ^13^C NMR spectrum of 1c (LASSBio-1535) (DMSO-*d_6_*, 50 MHz). Figure S8. ^1^H NMR spectrum of 1d (LASSBio-1695) (DMSO-*d_6_*, 200 MHz). Figure S9. ^13^C NMR spectrum of 1d (LASSBio-1695) (DMSO-*d_6_*, 50 MHz). Figure S10. ^1^H NMR spectrum of 1e (LASSBio-1696) (DMSO-*d_6_*, 200 MHz). Figure S11. ^13^C NMR spectrum of 1e (LASSBio-1696) (DMSO-*d_6_*, 50 MHz). Figure S12.^ 1^H NMR spectrum of 1f (LASSBio-1463) (DMSO-*d_6_*, 300 MHz). Figure S13.^ 1^H NMR spectrum of 1f (LASSBio-1463) (DMSO-*d_6_*, 300 MHz, T∼90°C). Figure S14.^ 13^C NMR spectrum of 1f (LASSBio-1463) (DMSO-*d_6_*, 50 MHz). Figure S15. ^1^H NMR spectrum of 1g (LASSBio-1626) (DMSO-*d_6_*, 300 MHz). Figure S16. ^1^H NMR spectrum of 1g (LASSBio-1626) (DMSO-*d_6_*, 300 MHz, T∼90°C). Figure S17. ^13^C NMR spectrum of 1g (LASSBio-1626) (DMSO-*d_6_*, 50 MHz). Figure S18. ^1^H NMR spectrum of 1h (LASSBio-1697) (DMSO-*d_6_*, 200 MHz). Figure S19. ^13^C NMR spectrum of 1h (LASSBio-1697) (DMSO-*d_6_*, 50 MHz). Figure S20. ^1^H NMR spectrum of 1i (LASSBio-1749) (DMSO-*d_6_*, 200 MHz). Figure S21. ^13^C NMR spectrum of 1i (LASSBio-1749) (DMSO-*d_6_*, 50 MHz). Figure S22. ^1^H NMR spectrum of 1j (LASSBio-1698) (DMSO-*d_6_*, 200 MHz). Figure S23. ^13^C NMR spectrum of 1j (LASSBio-1698) (DMSO-*d_6_*, 50 MHz). Figura S24.^ 1^H NMR spectrum of 1k (LASSBio-1615) (DMSO-*d_6_*, 200 MHz). Figura S25.^ 13^C NMR spectrum of 1k (LASSBio-1615) (DMSO-*d_6_*, 50 MHz). Figura S26. Reverse phase HPLC spectrum of 1f (LASSBio-1463) (acetonitrile:water (60∶40)). Table S1. p38α MAPK inhibitory activity of compounds (1a–c, 1 k) at 10 µM.
(ZIP)

## References

[1] Hotamisligil GS, Arner P, Caro JF, Atkinson RL, Spiegelman BM (1995) Increased adipose-tissue expression of tumor-necrosis-factor-alpha in human obesity and insulin-resistance. J Clin Invest 95: 2409–2415. 7738205 10.1172/JCI117936 PMC295872

[2] Moller DE (2000) Potential role of TNF-alpha in the pathogenesis of insulin resistance and type 2 diabetes. Trends Endocrinol Metab 11: 212–217. 10878750 10.1016/s1043-2760(00)00272-1

[3] Selmaj K, Raine CS, Cannella B, Brosnan CF (1991) Identification of lymphotoxin and tumor-necrosis-factor in multiple-sclerosis lesions. J Clin Invest 87: 949–954. 1999503 10.1172/JCI115102 PMC329886

[4] Campbell IK, Roberts LJ, Wicks IP (2003) Molecular targets in immune-mediated diseases: the case of tumour necrosis factor and rheumatoid arthritis. Immunol Cell Biol 81: 354–366. 12969323 10.1046/j.0818-9641.2003.01185.x

[5] Wong SH, Lord JM (2004) Factors underlying chronic inflammation in rheumatoid arthritis. Arch Immunol Ther Exp 52: 379–388. 15577739

[6] Toussirot E, Wendling D (2004) The use of TNF-alpha blocking agents in rheumatoid arthritis: an overview. Expert Opin Pharmacother 5: 581–594. 15013927 10.1517/14656566.5.3.581

[7] Atzeni F, Sarzi-Puttini P, Botsios C, Carletto A, Cipriani P, et al (2012) Long-term anti-TNF therapy and the risk of serious infections in a cohort of patients with rheumatoid arthritis: Comparison of adalimumab, etanercept and infliximab in the GISEA registry. Autoimmun Rev 12: 225–229. 22796281 10.1016/j.autrev.2012.06.008

[8] Lacerda RB, da Silva LL, de Lima CKF, Miguez E, Miranda ALP, et al (2012) Discovery of novel orally active anti-inflammatory *N*-phenylpyrazolyl-*N*-glycinyl-hydrazone derivatives that inhibit TNF-alpha production. PLoS One 7(10): e46925. 23056531 10.1371/journal.pone.0046925 PMC3466213

[9] Regan J, Breitfelder S, Cirillo P, Gilmore T, Graham AG, et al (2002) Pyrazole urea-based inhibitors of p38 MAP kinase: From lead compound to clinical candidate. J Med Chem 45: 2994–3008. 12086485 10.1021/jm020057r

[10] Regan J, Capolino A, Cirillo PF, Gilmore T, Graham AG, et al (2003) Structure-activity relationships of the p38 alpha MAP kinase inhibitor 1-(5-tert-Butyl-2-p-tolyl-2H-pyrazol-3-yl)-3-[4-(2-morpholin-4-yl-ethoxy)naphthalen-1-yl]urea (BIRB 796). J Med Chem 46: 4676–4686. 14561087 10.1021/jm030121k

[11] A Domling, Wang W, Wang K (2012) Chemistry and biology of multicomponent reactions. Chem Rev 112: 3083–3135. 22435608 10.1021/cr100233r PMC3712876

[12] Domling A, Ugi I (2000) Multicomponent reactions with isocyanides. Angew Chem Int Ed 39: 3169–3210. 10.1002/1521-3773(20000915)39:18<3168::aid-anie3168>3.0.co;2-u 11028061

[13] Slobbe P, Ruijter E, Orru RVA (2012) Recent applications of multicomponent reactions in medicinal chemistry. Med Chem Comm 3: 1189–1218.

[14] Lacerda RB, de Lima CKF, da Silva LL, Romeiro NC, Miranda ALP, et al (2009) Discovery of novel analgesic and anti-inflammatory 3-arylamine-imidazo[1,2-*a*] pyridine symbiotic prototypes. Bioorg Med Chem 17: 74–84. 19059783 10.1016/j.bmc.2008.11.018

[15] Zhu DJ, Chen JX, Liu MC, Ding JC, Wu HY (2009) Catalyst- and solvent-free synthesis of imidazo[1,2-*a*]pyridines. J Braz Chem Soc 20: 482–U130.

[16] Patel HS, Linn JA, Drewry DH, Hillesheim DA, Zuercher WJ, et al (2003) New synthetic approaches to estrogen receptor modulators: imidazo[1,2-*a*]pyridines. Tetrahedron Lett 44: 4077–4080.

[17] Ismail MA, Brun R, Wenzler T, Tanious FA, Wilson WD, et al (2004) Novel dicationic imidazo[1,2-*a*]pyridines and 5,6,7,8-tetrahydro-imidazo[1,2-*a*]pyridines as antiprotozoal agents. J Med Chem 47: 3658–3664. 15214792 10.1021/jm0400092

[18] DiMauro EF, Vitullo JR (2006) Microwave-assisted preparation of fused bicyclic heteroarylboronates: Application in one-pot Suzuki couplings. J Org Chem 71: 3959–3962. 16674073 10.1021/jo060218p

[19] Groebke K, Weber L, Mehlin F (1998) Synthesis of imidazo[1,2-*a*] annulated pyridines, pyrazines and pyrimidines by a novel three-component condensation. Synlett 661–663.

[20] Palla G, Predieri G, Domiano P, Vignali C, Turner W (1986) Conformational behavior and E/Z isomerization of *N*-acyl and *N*-aroylhydrazones. Tetrahedron 42: 3649–3654.

[21] Palla G, Pelizzi C, Predieri G, Vignali C (1982) Conformational study on *N*-acylhydrazones of aromatic-aldehydes by nmr-spectroscopy. Gazz Chim Ital 112: 339–341.

[22] Wyrzykiewicz E, Blaszczyk A (2000) New isomeric *N*-substituted hydrazones of 2-, 3-and 4-pyridinecarboxaldehydes and methyl-3-pyridylketone. J Heterocyclic Chem 37: 975–981.

[23] Lopes AB, Miguez E, Kümmerle AE, Rumjanek V, Fraga CAM, et al (2013) Characterization of amide bond conformers for a novel heterocyclic template of *N*-acylhydrazone derivatives. Molecules 18: 11683–11704. 24071978 10.3390/molecules181011683 PMC6270085

[24] Gallily R, Yamin A, Waksmann Y, Ovadia H, Weidenfeld J, et al (1997) Protection against septic shock and suppression of tumor necrosis factor alpha and nitric oxide production by dexanabinol (HU-211), a nonpsychotropic cannabinoid. J Pharmacol Exp Ther 283: 918–924. 9353414

[25] Goettert M, Graeser R, Laufer SA (2010) Optimization of a nonradioactive immunosorbent assay for p38 alpha mitogen-activated protein kinase activity. Anal Biochem 406: 233–234. 20638357 10.1016/j.ab.2010.07.007

[26] Mosmann T (1983) Rapid colorimetric assay for cellular growth and survival – Application to proliferation and cytotoxicity assays. J Immunol Methods 65: 55–63. 6606682 10.1016/0022-1759(83)90303-4

[27] Romano M, Faggioni R, Sironi M, Sacco S, Echtenacher B, et al (1997) Carrageenan-induced acute inflammation in the mouse air pouch synovial model, Role of tumour necrosis factor. Mediators Inflamm 6: 32–38. 18472831 10.1080/09629359791901 PMC2365839

[28] Raymundo LJ, Guilhon CC, Alviano DS, Matheus ME, Antoniolli AR, et al (2011) Characterisation of the anti-inflammatory and antinociceptive activities of the Hyptis pectinata (L.) Poit essential oil. J Ethnopharmacol 134: 725–732. 21277967 10.1016/j.jep.2011.01.027

