# Supplementary figures and images for: Novel Potent Imidazo[1,2-a]pyridine-N-Glycinyl-Hydrazone Inhibitors of TNF-α Production: In Vitro and In Vivo Studies
Source: PLoS One. 2014 Mar 14;9(3):e91660. doi: 10.1371/journal.pone.0091660 (PMC3954757; doi:10.1371/journal.pone.0091660)

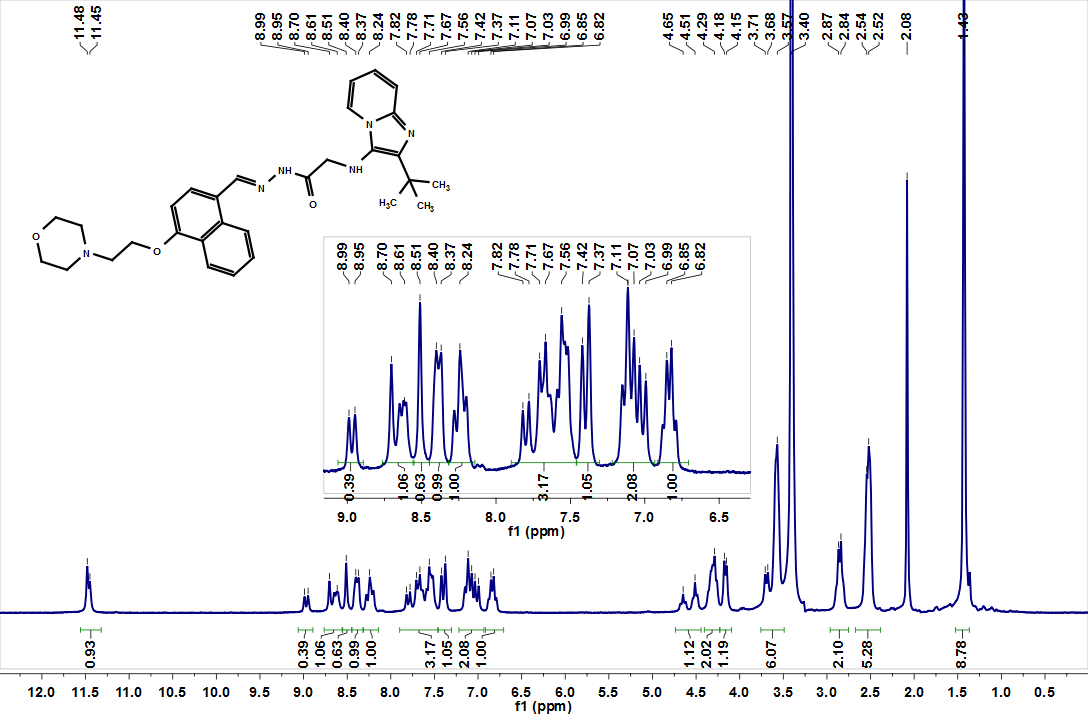

Supplement: File S1 — Figures S1–S26 and Table S1. Figure S1. 1H NMR spectrum of 1a (LASSBio-1507) (DMSO-d6, 200 MHz). Figure S2. 13C NMR spectrum of 1a (LASSBio-1507) (DMSO-d6, 50 MHz). Figure S3. 1H NMR spectrum of 1b (LASSBio-1616) (DMSO-d6, 200 MHz). Figure S4. 1H NMR spectrum of 1b (LASSBio-1616) (CDCl3, 200 MHz). Figure S5. 13C NMR spectrum of 1b (LASSBio-1616) (CDCl3, 50 MHz). Figure S6. 1H NMR spectrum of 1c (LASSBio-1535) (DMSO-d6, 200 MHz). Figure S7. 13C NMR spectrum of 1c (LASSBio-1535) (DMSO-d6, 50 MHz). Figure S8. 1H NMR spectrum of 1d (LASSBio-1695) (DMSO-d6, 200 MHz). Figure S9. 13C NMR spectrum of 1d (LASSBio-1695) (DMSO-d6, 50 MHz). Figure S10. 1H NMR spectrum of 1e (LASSBio-1696) (DMSO-d6, 200 MHz). Figure S11. 13C NMR spectrum of 1e (LASSBio-1696) (DMSO-d6, 50 MHz). Figure S12. 1H NMR spectrum of 1f (LASSBio-1463) (DMSO-d6, 300 MHz). Figure S13. 1H NMR spectrum of 1f (LASSBio-1463) (DMSO-d6, 300 MHz, T∼90°C). Figure S14. 13C NMR spectrum of 1f (LASSBio-1463) (DMSO-d6, 50 MHz). Figure S15. 1H NMR spectrum of 1g (LASSBio-1626) (DMSO-d6, 300 MHz). Figure S16. 1H NMR spectrum of 1g (LASSBio-1626) (DMSO-d6, 300 MHz, T∼90°C). Figure S17. 13C NMR spectrum of 1g (LASSBio-1626) (DMSO-d6, 50 MHz). Figure S18. 1H NMR spectrum of 1h (LASSBio-1697) (DMSO-d6, 200 MHz). Figure S19. 13C NMR spectrum of 1h (LASSBio-1697) (DMSO-d6, 50 MHz). Figure S20. 1H NMR spectrum of 1i (LASSBio-1749) (DMSO-d6, 200 MHz). Figure S21. 13C NMR spectrum of 1i (LASSBio-1749) (DMSO-d6, 50 MHz). Figure S22. 1H NMR spectrum of 1j (LASSBio-1698) (DMSO-d6, 200 MHz). Figure S23. 13C NMR spectrum of 1j (LASSBio-1698) (DMSO-d6, 50 MHz). Figura S24. 1H NMR spectrum of 1k (LASSBio-1615) (DMSO-d6, 200 MHz). Figura S25. 13C NMR spectrum of 1k (LASSBio-1615) (DMSO-d6, 50 MHz). Figura S26. Reverse phase HPLC spectrum of 1f (LASSBio-1463) (acetonitrile:water (60∶40)). Table S1. p38α MAPK inhibitory activity of compounds (1a–c, 1 k) at 10 µM. (ZIP) [file pone.0091660.s001.zip › Figure S1.tiff]

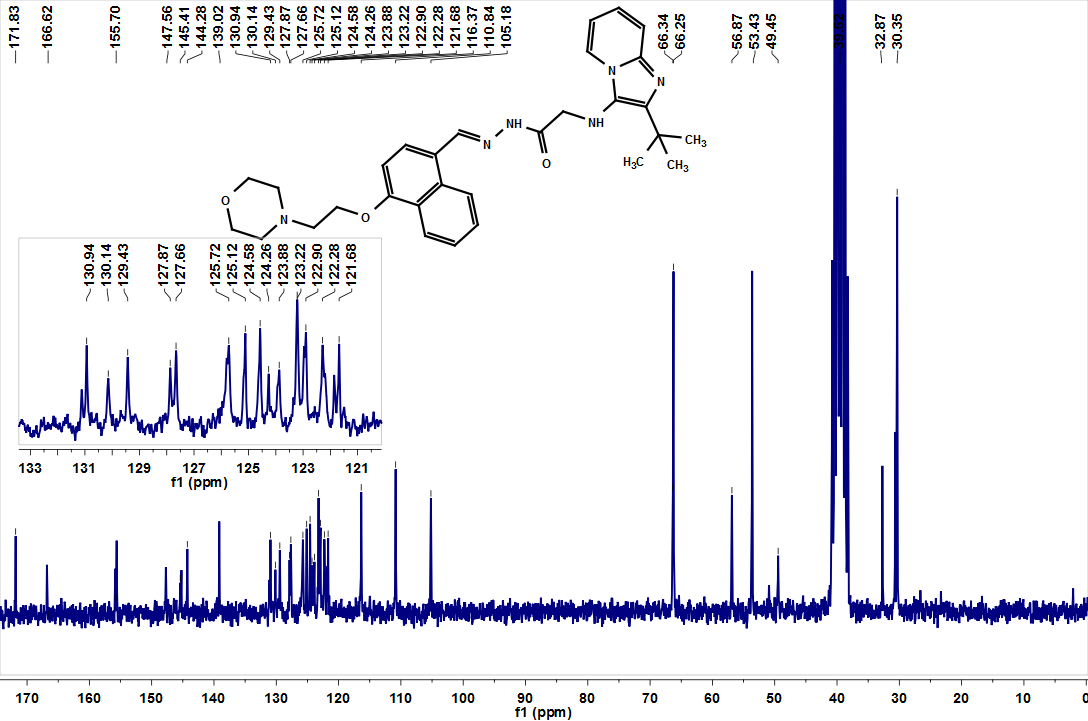

Supplement: File S1 — Figures S1–S26 and Table S1. Figure S1. 1H NMR spectrum of 1a (LASSBio-1507) (DMSO-d6, 200 MHz). Figure S2. 13C NMR spectrum of 1a (LASSBio-1507) (DMSO-d6, 50 MHz). Figure S3. 1H NMR spectrum of 1b (LASSBio-1616) (DMSO-d6, 200 MHz). Figure S4. 1H NMR spectrum of 1b (LASSBio-1616) (CDCl3, 200 MHz). Figure S5. 13C NMR spectrum of 1b (LASSBio-1616) (CDCl3, 50 MHz). Figure S6. 1H NMR spectrum of 1c (LASSBio-1535) (DMSO-d6, 200 MHz). Figure S7. 13C NMR spectrum of 1c (LASSBio-1535) (DMSO-d6, 50 MHz). Figure S8. 1H NMR spectrum of 1d (LASSBio-1695) (DMSO-d6, 200 MHz). Figure S9. 13C NMR spectrum of 1d (LASSBio-1695) (DMSO-d6, 50 MHz). Figure S10. 1H NMR spectrum of 1e (LASSBio-1696) (DMSO-d6, 200 MHz). Figure S11. 13C NMR spectrum of 1e (LASSBio-1696) (DMSO-d6, 50 MHz). Figure S12. 1H NMR spectrum of 1f (LASSBio-1463) (DMSO-d6, 300 MHz). Figure S13. 1H NMR spectrum of 1f (LASSBio-1463) (DMSO-d6, 300 MHz, T∼90°C). Figure S14. 13C NMR spectrum of 1f (LASSBio-1463) (DMSO-d6, 50 MHz). Figure S15. 1H NMR spectrum of 1g (LASSBio-1626) (DMSO-d6, 300 MHz). Figure S16. 1H NMR spectrum of 1g (LASSBio-1626) (DMSO-d6, 300 MHz, T∼90°C). Figure S17. 13C NMR spectrum of 1g (LASSBio-1626) (DMSO-d6, 50 MHz). Figure S18. 1H NMR spectrum of 1h (LASSBio-1697) (DMSO-d6, 200 MHz). Figure S19. 13C NMR spectrum of 1h (LASSBio-1697) (DMSO-d6, 50 MHz). Figure S20. 1H NMR spectrum of 1i (LASSBio-1749) (DMSO-d6, 200 MHz). Figure S21. 13C NMR spectrum of 1i (LASSBio-1749) (DMSO-d6, 50 MHz). Figure S22. 1H NMR spectrum of 1j (LASSBio-1698) (DMSO-d6, 200 MHz). Figure S23. 13C NMR spectrum of 1j (LASSBio-1698) (DMSO-d6, 50 MHz). Figura S24. 1H NMR spectrum of 1k (LASSBio-1615) (DMSO-d6, 200 MHz). Figura S25. 13C NMR spectrum of 1k (LASSBio-1615) (DMSO-d6, 50 MHz). Figura S26. Reverse phase HPLC spectrum of 1f (LASSBio-1463) (acetonitrile:water (60∶40)). Table S1. p38α MAPK inhibitory activity of compounds (1a–c, 1 k) at 10 µM. (ZIP) [file pone.0091660.s001.zip › Figure S2.tiff]

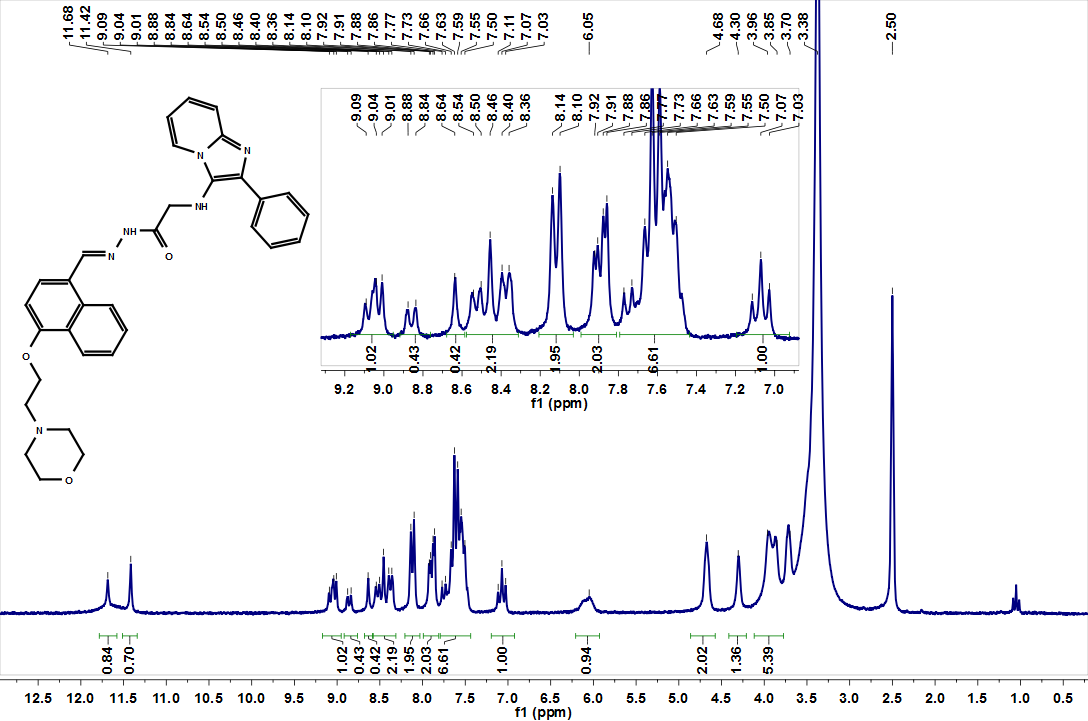

Supplement: File S1 — Figures S1–S26 and Table S1. Figure S1. 1H NMR spectrum of 1a (LASSBio-1507) (DMSO-d6, 200 MHz). Figure S2. 13C NMR spectrum of 1a (LASSBio-1507) (DMSO-d6, 50 MHz). Figure S3. 1H NMR spectrum of 1b (LASSBio-1616) (DMSO-d6, 200 MHz). Figure S4. 1H NMR spectrum of 1b (LASSBio-1616) (CDCl3, 200 MHz). Figure S5. 13C NMR spectrum of 1b (LASSBio-1616) (CDCl3, 50 MHz). Figure S6. 1H NMR spectrum of 1c (LASSBio-1535) (DMSO-d6, 200 MHz). Figure S7. 13C NMR spectrum of 1c (LASSBio-1535) (DMSO-d6, 50 MHz). Figure S8. 1H NMR spectrum of 1d (LASSBio-1695) (DMSO-d6, 200 MHz). Figure S9. 13C NMR spectrum of 1d (LASSBio-1695) (DMSO-d6, 50 MHz). Figure S10. 1H NMR spectrum of 1e (LASSBio-1696) (DMSO-d6, 200 MHz). Figure S11. 13C NMR spectrum of 1e (LASSBio-1696) (DMSO-d6, 50 MHz). Figure S12. 1H NMR spectrum of 1f (LASSBio-1463) (DMSO-d6, 300 MHz). Figure S13. 1H NMR spectrum of 1f (LASSBio-1463) (DMSO-d6, 300 MHz, T∼90°C). Figure S14. 13C NMR spectrum of 1f (LASSBio-1463) (DMSO-d6, 50 MHz). Figure S15. 1H NMR spectrum of 1g (LASSBio-1626) (DMSO-d6, 300 MHz). Figure S16. 1H NMR spectrum of 1g (LASSBio-1626) (DMSO-d6, 300 MHz, T∼90°C). Figure S17. 13C NMR spectrum of 1g (LASSBio-1626) (DMSO-d6, 50 MHz). Figure S18. 1H NMR spectrum of 1h (LASSBio-1697) (DMSO-d6, 200 MHz). Figure S19. 13C NMR spectrum of 1h (LASSBio-1697) (DMSO-d6, 50 MHz). Figure S20. 1H NMR spectrum of 1i (LASSBio-1749) (DMSO-d6, 200 MHz). Figure S21. 13C NMR spectrum of 1i (LASSBio-1749) (DMSO-d6, 50 MHz). Figure S22. 1H NMR spectrum of 1j (LASSBio-1698) (DMSO-d6, 200 MHz). Figure S23. 13C NMR spectrum of 1j (LASSBio-1698) (DMSO-d6, 50 MHz). Figura S24. 1H NMR spectrum of 1k (LASSBio-1615) (DMSO-d6, 200 MHz). Figura S25. 13C NMR spectrum of 1k (LASSBio-1615) (DMSO-d6, 50 MHz). Figura S26. Reverse phase HPLC spectrum of 1f (LASSBio-1463) (acetonitrile:water (60∶40)). Table S1. p38α MAPK inhibitory activity of compounds (1a–c, 1 k) at 10 µM. (ZIP) [file pone.0091660.s001.zip › Figure S3.tiff]

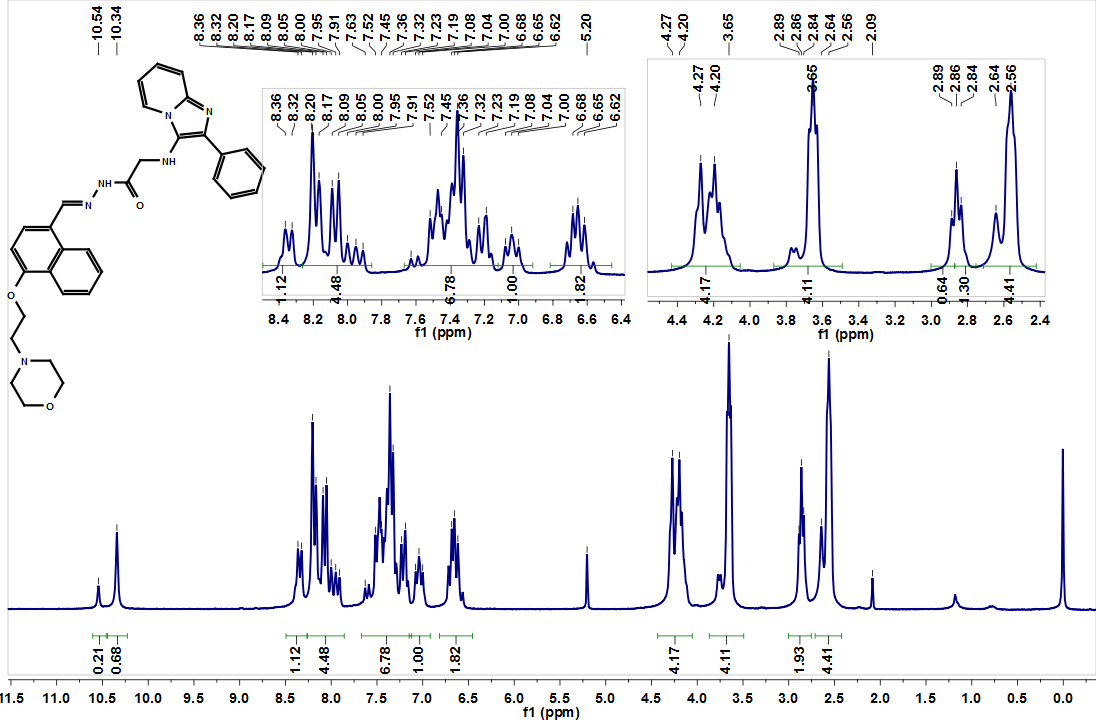

Supplement: File S1 — Figures S1–S26 and Table S1. Figure S1. 1H NMR spectrum of 1a (LASSBio-1507) (DMSO-d6, 200 MHz). Figure S2. 13C NMR spectrum of 1a (LASSBio-1507) (DMSO-d6, 50 MHz). Figure S3. 1H NMR spectrum of 1b (LASSBio-1616) (DMSO-d6, 200 MHz). Figure S4. 1H NMR spectrum of 1b (LASSBio-1616) (CDCl3, 200 MHz). Figure S5. 13C NMR spectrum of 1b (LASSBio-1616) (CDCl3, 50 MHz). Figure S6. 1H NMR spectrum of 1c (LASSBio-1535) (DMSO-d6, 200 MHz). Figure S7. 13C NMR spectrum of 1c (LASSBio-1535) (DMSO-d6, 50 MHz). Figure S8. 1H NMR spectrum of 1d (LASSBio-1695) (DMSO-d6, 200 MHz). Figure S9. 13C NMR spectrum of 1d (LASSBio-1695) (DMSO-d6, 50 MHz). Figure S10. 1H NMR spectrum of 1e (LASSBio-1696) (DMSO-d6, 200 MHz). Figure S11. 13C NMR spectrum of 1e (LASSBio-1696) (DMSO-d6, 50 MHz). Figure S12. 1H NMR spectrum of 1f (LASSBio-1463) (DMSO-d6, 300 MHz). Figure S13. 1H NMR spectrum of 1f (LASSBio-1463) (DMSO-d6, 300 MHz, T∼90°C). Figure S14. 13C NMR spectrum of 1f (LASSBio-1463) (DMSO-d6, 50 MHz). Figure S15. 1H NMR spectrum of 1g (LASSBio-1626) (DMSO-d6, 300 MHz). Figure S16. 1H NMR spectrum of 1g (LASSBio-1626) (DMSO-d6, 300 MHz, T∼90°C). Figure S17. 13C NMR spectrum of 1g (LASSBio-1626) (DMSO-d6, 50 MHz). Figure S18. 1H NMR spectrum of 1h (LASSBio-1697) (DMSO-d6, 200 MHz). Figure S19. 13C NMR spectrum of 1h (LASSBio-1697) (DMSO-d6, 50 MHz). Figure S20. 1H NMR spectrum of 1i (LASSBio-1749) (DMSO-d6, 200 MHz). Figure S21. 13C NMR spectrum of 1i (LASSBio-1749) (DMSO-d6, 50 MHz). Figure S22. 1H NMR spectrum of 1j (LASSBio-1698) (DMSO-d6, 200 MHz). Figure S23. 13C NMR spectrum of 1j (LASSBio-1698) (DMSO-d6, 50 MHz). Figura S24. 1H NMR spectrum of 1k (LASSBio-1615) (DMSO-d6, 200 MHz). Figura S25. 13C NMR spectrum of 1k (LASSBio-1615) (DMSO-d6, 50 MHz). Figura S26. Reverse phase HPLC spectrum of 1f (LASSBio-1463) (acetonitrile:water (60∶40)). Table S1. p38α MAPK inhibitory activity of compounds (1a–c, 1 k) at 10 µM. (ZIP) [file pone.0091660.s001.zip › Figure S4.tiff]

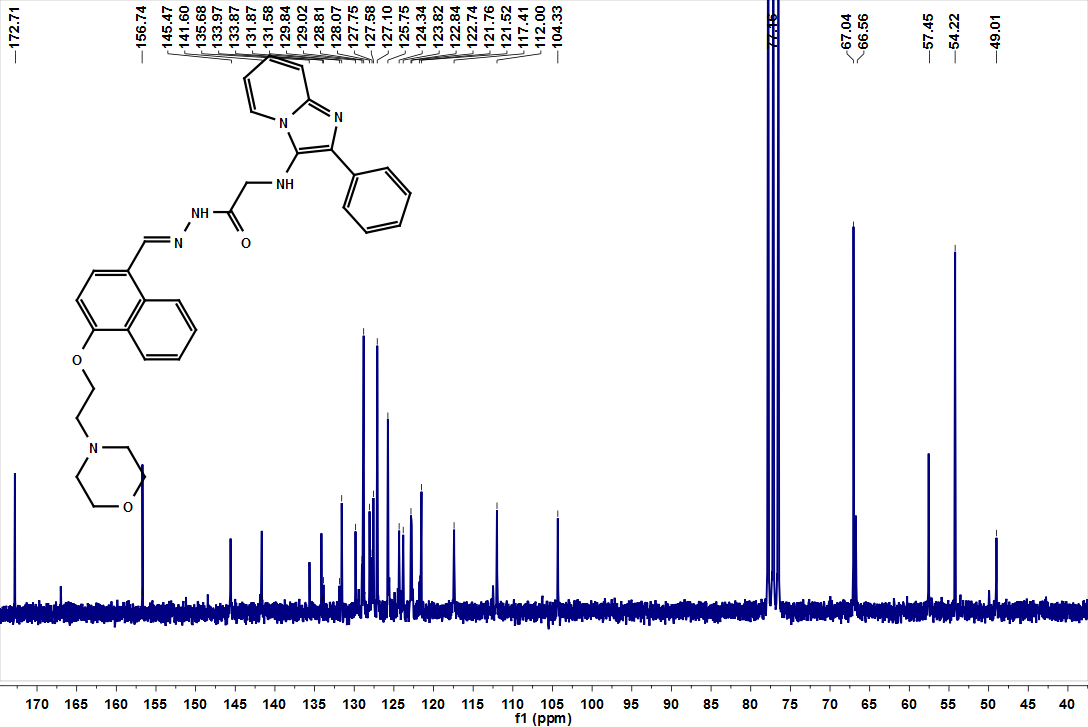

Supplement: File S1 — Figures S1–S26 and Table S1. Figure S1. 1H NMR spectrum of 1a (LASSBio-1507) (DMSO-d6, 200 MHz). Figure S2. 13C NMR spectrum of 1a (LASSBio-1507) (DMSO-d6, 50 MHz). Figure S3. 1H NMR spectrum of 1b (LASSBio-1616) (DMSO-d6, 200 MHz). Figure S4. 1H NMR spectrum of 1b (LASSBio-1616) (CDCl3, 200 MHz). Figure S5. 13C NMR spectrum of 1b (LASSBio-1616) (CDCl3, 50 MHz). Figure S6. 1H NMR spectrum of 1c (LASSBio-1535) (DMSO-d6, 200 MHz). Figure S7. 13C NMR spectrum of 1c (LASSBio-1535) (DMSO-d6, 50 MHz). Figure S8. 1H NMR spectrum of 1d (LASSBio-1695) (DMSO-d6, 200 MHz). Figure S9. 13C NMR spectrum of 1d (LASSBio-1695) (DMSO-d6, 50 MHz). Figure S10. 1H NMR spectrum of 1e (LASSBio-1696) (DMSO-d6, 200 MHz). Figure S11. 13C NMR spectrum of 1e (LASSBio-1696) (DMSO-d6, 50 MHz). Figure S12. 1H NMR spectrum of 1f (LASSBio-1463) (DMSO-d6, 300 MHz). Figure S13. 1H NMR spectrum of 1f (LASSBio-1463) (DMSO-d6, 300 MHz, T∼90°C). Figure S14. 13C NMR spectrum of 1f (LASSBio-1463) (DMSO-d6, 50 MHz). Figure S15. 1H NMR spectrum of 1g (LASSBio-1626) (DMSO-d6, 300 MHz). Figure S16. 1H NMR spectrum of 1g (LASSBio-1626) (DMSO-d6, 300 MHz, T∼90°C). Figure S17. 13C NMR spectrum of 1g (LASSBio-1626) (DMSO-d6, 50 MHz). Figure S18. 1H NMR spectrum of 1h (LASSBio-1697) (DMSO-d6, 200 MHz). Figure S19. 13C NMR spectrum of 1h (LASSBio-1697) (DMSO-d6, 50 MHz). Figure S20. 1H NMR spectrum of 1i (LASSBio-1749) (DMSO-d6, 200 MHz). Figure S21. 13C NMR spectrum of 1i (LASSBio-1749) (DMSO-d6, 50 MHz). Figure S22. 1H NMR spectrum of 1j (LASSBio-1698) (DMSO-d6, 200 MHz). Figure S23. 13C NMR spectrum of 1j (LASSBio-1698) (DMSO-d6, 50 MHz). Figura S24. 1H NMR spectrum of 1k (LASSBio-1615) (DMSO-d6, 200 MHz). Figura S25. 13C NMR spectrum of 1k (LASSBio-1615) (DMSO-d6, 50 MHz). Figura S26. Reverse phase HPLC spectrum of 1f (LASSBio-1463) (acetonitrile:water (60∶40)). Table S1. p38α MAPK inhibitory activity of compounds (1a–c, 1 k) at 10 µM. (ZIP) [file pone.0091660.s001.zip › Figure S5.tiff]

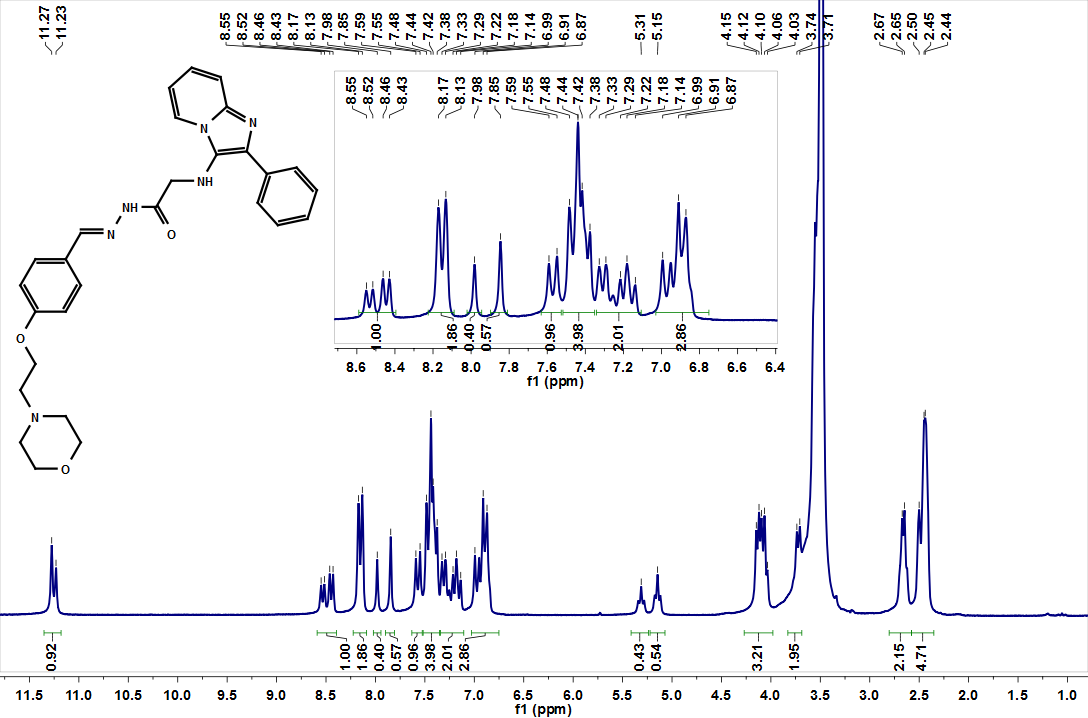

Supplement: File S1 — Figures S1–S26 and Table S1. Figure S1. 1H NMR spectrum of 1a (LASSBio-1507) (DMSO-d6, 200 MHz). Figure S2. 13C NMR spectrum of 1a (LASSBio-1507) (DMSO-d6, 50 MHz). Figure S3. 1H NMR spectrum of 1b (LASSBio-1616) (DMSO-d6, 200 MHz). Figure S4. 1H NMR spectrum of 1b (LASSBio-1616) (CDCl3, 200 MHz). Figure S5. 13C NMR spectrum of 1b (LASSBio-1616) (CDCl3, 50 MHz). Figure S6. 1H NMR spectrum of 1c (LASSBio-1535) (DMSO-d6, 200 MHz). Figure S7. 13C NMR spectrum of 1c (LASSBio-1535) (DMSO-d6, 50 MHz). Figure S8. 1H NMR spectrum of 1d (LASSBio-1695) (DMSO-d6, 200 MHz). Figure S9. 13C NMR spectrum of 1d (LASSBio-1695) (DMSO-d6, 50 MHz). Figure S10. 1H NMR spectrum of 1e (LASSBio-1696) (DMSO-d6, 200 MHz). Figure S11. 13C NMR spectrum of 1e (LASSBio-1696) (DMSO-d6, 50 MHz). Figure S12. 1H NMR spectrum of 1f (LASSBio-1463) (DMSO-d6, 300 MHz). Figure S13. 1H NMR spectrum of 1f (LASSBio-1463) (DMSO-d6, 300 MHz, T∼90°C). Figure S14. 13C NMR spectrum of 1f (LASSBio-1463) (DMSO-d6, 50 MHz). Figure S15. 1H NMR spectrum of 1g (LASSBio-1626) (DMSO-d6, 300 MHz). Figure S16. 1H NMR spectrum of 1g (LASSBio-1626) (DMSO-d6, 300 MHz, T∼90°C). Figure S17. 13C NMR spectrum of 1g (LASSBio-1626) (DMSO-d6, 50 MHz). Figure S18. 1H NMR spectrum of 1h (LASSBio-1697) (DMSO-d6, 200 MHz). Figure S19. 13C NMR spectrum of 1h (LASSBio-1697) (DMSO-d6, 50 MHz). Figure S20. 1H NMR spectrum of 1i (LASSBio-1749) (DMSO-d6, 200 MHz). Figure S21. 13C NMR spectrum of 1i (LASSBio-1749) (DMSO-d6, 50 MHz). Figure S22. 1H NMR spectrum of 1j (LASSBio-1698) (DMSO-d6, 200 MHz). Figure S23. 13C NMR spectrum of 1j (LASSBio-1698) (DMSO-d6, 50 MHz). Figura S24. 1H NMR spectrum of 1k (LASSBio-1615) (DMSO-d6, 200 MHz). Figura S25. 13C NMR spectrum of 1k (LASSBio-1615) (DMSO-d6, 50 MHz). Figura S26. Reverse phase HPLC spectrum of 1f (LASSBio-1463) (acetonitrile:water (60∶40)). Table S1. p38α MAPK inhibitory activity of compounds (1a–c, 1 k) at 10 µM. (ZIP) [file pone.0091660.s001.zip › Figure S6.tiff]

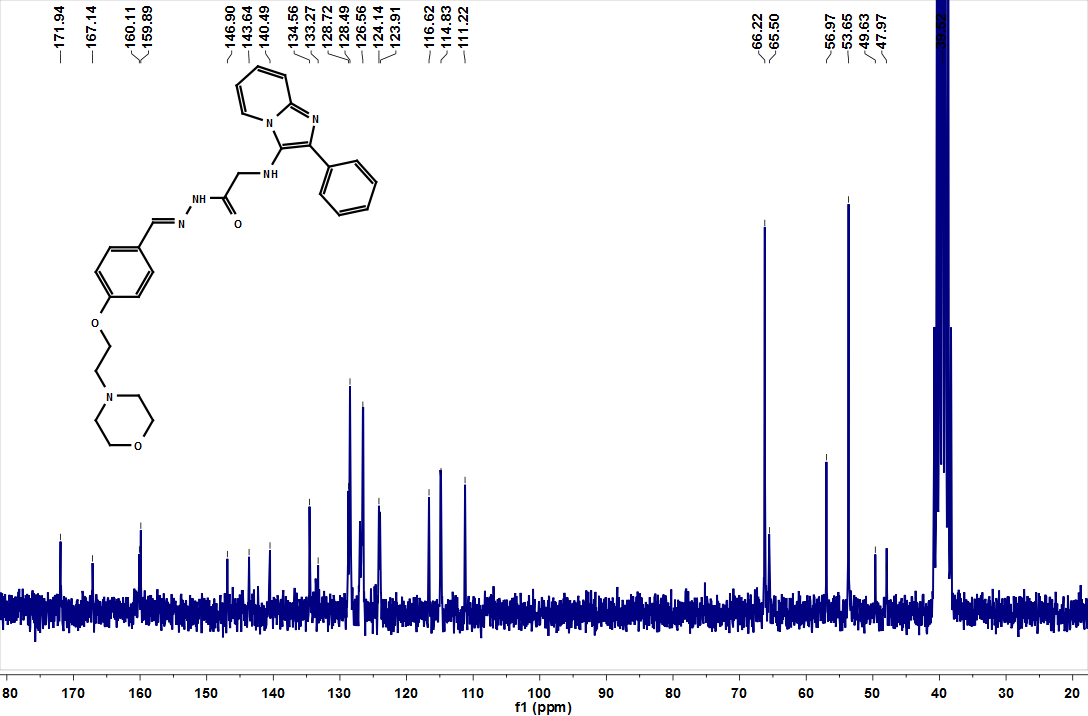

Supplement: File S1 — Figures S1–S26 and Table S1. Figure S1. 1H NMR spectrum of 1a (LASSBio-1507) (DMSO-d6, 200 MHz). Figure S2. 13C NMR spectrum of 1a (LASSBio-1507) (DMSO-d6, 50 MHz). Figure S3. 1H NMR spectrum of 1b (LASSBio-1616) (DMSO-d6, 200 MHz). Figure S4. 1H NMR spectrum of 1b (LASSBio-1616) (CDCl3, 200 MHz). Figure S5. 13C NMR spectrum of 1b (LASSBio-1616) (CDCl3, 50 MHz). Figure S6. 1H NMR spectrum of 1c (LASSBio-1535) (DMSO-d6, 200 MHz). Figure S7. 13C NMR spectrum of 1c (LASSBio-1535) (DMSO-d6, 50 MHz). Figure S8. 1H NMR spectrum of 1d (LASSBio-1695) (DMSO-d6, 200 MHz). Figure S9. 13C NMR spectrum of 1d (LASSBio-1695) (DMSO-d6, 50 MHz). Figure S10. 1H NMR spectrum of 1e (LASSBio-1696) (DMSO-d6, 200 MHz). Figure S11. 13C NMR spectrum of 1e (LASSBio-1696) (DMSO-d6, 50 MHz). Figure S12. 1H NMR spectrum of 1f (LASSBio-1463) (DMSO-d6, 300 MHz). Figure S13. 1H NMR spectrum of 1f (LASSBio-1463) (DMSO-d6, 300 MHz, T∼90°C). Figure S14. 13C NMR spectrum of 1f (LASSBio-1463) (DMSO-d6, 50 MHz). Figure S15. 1H NMR spectrum of 1g (LASSBio-1626) (DMSO-d6, 300 MHz). Figure S16. 1H NMR spectrum of 1g (LASSBio-1626) (DMSO-d6, 300 MHz, T∼90°C). Figure S17. 13C NMR spectrum of 1g (LASSBio-1626) (DMSO-d6, 50 MHz). Figure S18. 1H NMR spectrum of 1h (LASSBio-1697) (DMSO-d6, 200 MHz). Figure S19. 13C NMR spectrum of 1h (LASSBio-1697) (DMSO-d6, 50 MHz). Figure S20. 1H NMR spectrum of 1i (LASSBio-1749) (DMSO-d6, 200 MHz). Figure S21. 13C NMR spectrum of 1i (LASSBio-1749) (DMSO-d6, 50 MHz). Figure S22. 1H NMR spectrum of 1j (LASSBio-1698) (DMSO-d6, 200 MHz). Figure S23. 13C NMR spectrum of 1j (LASSBio-1698) (DMSO-d6, 50 MHz). Figura S24. 1H NMR spectrum of 1k (LASSBio-1615) (DMSO-d6, 200 MHz). Figura S25. 13C NMR spectrum of 1k (LASSBio-1615) (DMSO-d6, 50 MHz). Figura S26. Reverse phase HPLC spectrum of 1f (LASSBio-1463) (acetonitrile:water (60∶40)). Table S1. p38α MAPK inhibitory activity of compounds (1a–c, 1 k) at 10 µM. (ZIP) [file pone.0091660.s001.zip › Figure S7.tiff]

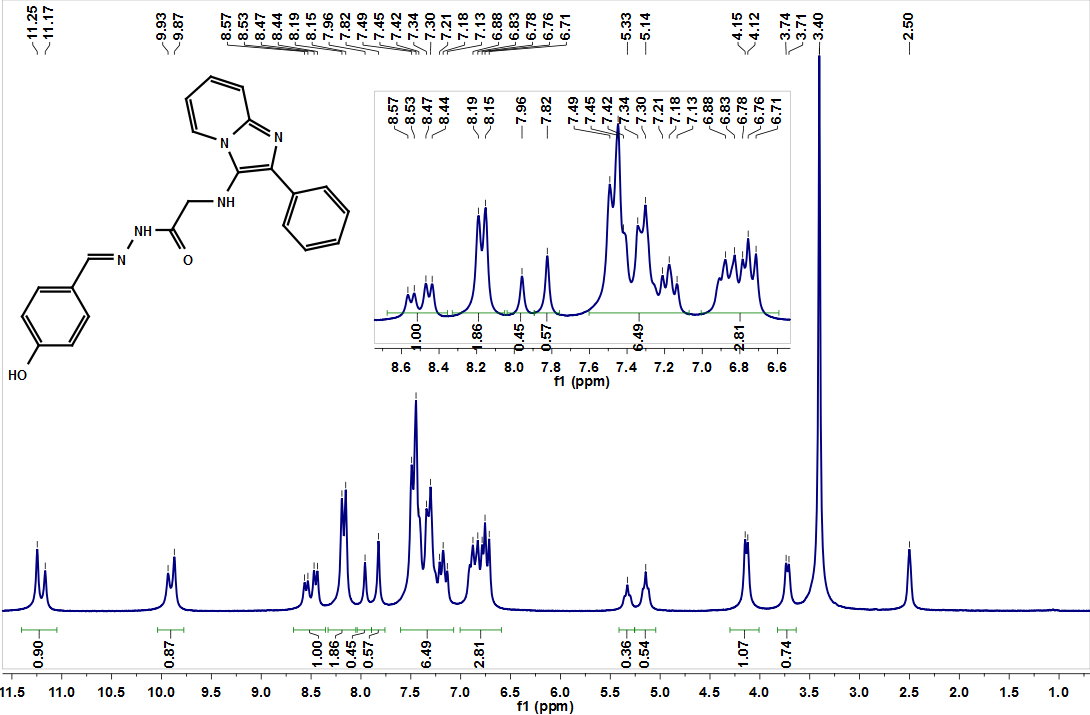

Supplement: File S1 — Figures S1–S26 and Table S1. Figure S1. 1H NMR spectrum of 1a (LASSBio-1507) (DMSO-d6, 200 MHz). Figure S2. 13C NMR spectrum of 1a (LASSBio-1507) (DMSO-d6, 50 MHz). Figure S3. 1H NMR spectrum of 1b (LASSBio-1616) (DMSO-d6, 200 MHz). Figure S4. 1H NMR spectrum of 1b (LASSBio-1616) (CDCl3, 200 MHz). Figure S5. 13C NMR spectrum of 1b (LASSBio-1616) (CDCl3, 50 MHz). Figure S6. 1H NMR spectrum of 1c (LASSBio-1535) (DMSO-d6, 200 MHz). Figure S7. 13C NMR spectrum of 1c (LASSBio-1535) (DMSO-d6, 50 MHz). Figure S8. 1H NMR spectrum of 1d (LASSBio-1695) (DMSO-d6, 200 MHz). Figure S9. 13C NMR spectrum of 1d (LASSBio-1695) (DMSO-d6, 50 MHz). Figure S10. 1H NMR spectrum of 1e (LASSBio-1696) (DMSO-d6, 200 MHz). Figure S11. 13C NMR spectrum of 1e (LASSBio-1696) (DMSO-d6, 50 MHz). Figure S12. 1H NMR spectrum of 1f (LASSBio-1463) (DMSO-d6, 300 MHz). Figure S13. 1H NMR spectrum of 1f (LASSBio-1463) (DMSO-d6, 300 MHz, T∼90°C). Figure S14. 13C NMR spectrum of 1f (LASSBio-1463) (DMSO-d6, 50 MHz). Figure S15. 1H NMR spectrum of 1g (LASSBio-1626) (DMSO-d6, 300 MHz). Figure S16. 1H NMR spectrum of 1g (LASSBio-1626) (DMSO-d6, 300 MHz, T∼90°C). Figure S17. 13C NMR spectrum of 1g (LASSBio-1626) (DMSO-d6, 50 MHz). Figure S18. 1H NMR spectrum of 1h (LASSBio-1697) (DMSO-d6, 200 MHz). Figure S19. 13C NMR spectrum of 1h (LASSBio-1697) (DMSO-d6, 50 MHz). Figure S20. 1H NMR spectrum of 1i (LASSBio-1749) (DMSO-d6, 200 MHz). Figure S21. 13C NMR spectrum of 1i (LASSBio-1749) (DMSO-d6, 50 MHz). Figure S22. 1H NMR spectrum of 1j (LASSBio-1698) (DMSO-d6, 200 MHz). Figure S23. 13C NMR spectrum of 1j (LASSBio-1698) (DMSO-d6, 50 MHz). Figura S24. 1H NMR spectrum of 1k (LASSBio-1615) (DMSO-d6, 200 MHz). Figura S25. 13C NMR spectrum of 1k (LASSBio-1615) (DMSO-d6, 50 MHz). Figura S26. Reverse phase HPLC spectrum of 1f (LASSBio-1463) (acetonitrile:water (60∶40)). Table S1. p38α MAPK inhibitory activity of compounds (1a–c, 1 k) at 10 µM. (ZIP) [file pone.0091660.s001.zip › Figure S8.tiff]

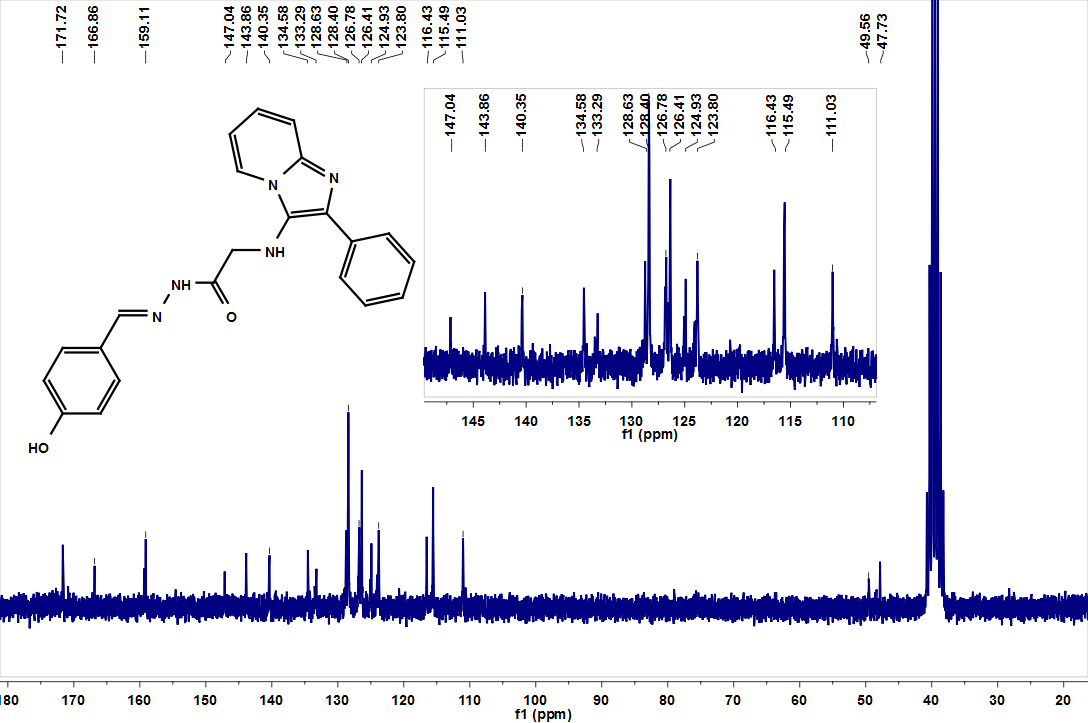

Supplement: File S1 — Figures S1–S26 and Table S1. Figure S1. 1H NMR spectrum of 1a (LASSBio-1507) (DMSO-d6, 200 MHz). Figure S2. 13C NMR spectrum of 1a (LASSBio-1507) (DMSO-d6, 50 MHz). Figure S3. 1H NMR spectrum of 1b (LASSBio-1616) (DMSO-d6, 200 MHz). Figure S4. 1H NMR spectrum of 1b (LASSBio-1616) (CDCl3, 200 MHz). Figure S5. 13C NMR spectrum of 1b (LASSBio-1616) (CDCl3, 50 MHz). Figure S6. 1H NMR spectrum of 1c (LASSBio-1535) (DMSO-d6, 200 MHz). Figure S7. 13C NMR spectrum of 1c (LASSBio-1535) (DMSO-d6, 50 MHz). Figure S8. 1H NMR spectrum of 1d (LASSBio-1695) (DMSO-d6, 200 MHz). Figure S9. 13C NMR spectrum of 1d (LASSBio-1695) (DMSO-d6, 50 MHz). Figure S10. 1H NMR spectrum of 1e (LASSBio-1696) (DMSO-d6, 200 MHz). Figure S11. 13C NMR spectrum of 1e (LASSBio-1696) (DMSO-d6, 50 MHz). Figure S12. 1H NMR spectrum of 1f (LASSBio-1463) (DMSO-d6, 300 MHz). Figure S13. 1H NMR spectrum of 1f (LASSBio-1463) (DMSO-d6, 300 MHz, T∼90°C). Figure S14. 13C NMR spectrum of 1f (LASSBio-1463) (DMSO-d6, 50 MHz). Figure S15. 1H NMR spectrum of 1g (LASSBio-1626) (DMSO-d6, 300 MHz). Figure S16. 1H NMR spectrum of 1g (LASSBio-1626) (DMSO-d6, 300 MHz, T∼90°C). Figure S17. 13C NMR spectrum of 1g (LASSBio-1626) (DMSO-d6, 50 MHz). Figure S18. 1H NMR spectrum of 1h (LASSBio-1697) (DMSO-d6, 200 MHz). Figure S19. 13C NMR spectrum of 1h (LASSBio-1697) (DMSO-d6, 50 MHz). Figure S20. 1H NMR spectrum of 1i (LASSBio-1749) (DMSO-d6, 200 MHz). Figure S21. 13C NMR spectrum of 1i (LASSBio-1749) (DMSO-d6, 50 MHz). Figure S22. 1H NMR spectrum of 1j (LASSBio-1698) (DMSO-d6, 200 MHz). Figure S23. 13C NMR spectrum of 1j (LASSBio-1698) (DMSO-d6, 50 MHz). Figura S24. 1H NMR spectrum of 1k (LASSBio-1615) (DMSO-d6, 200 MHz). Figura S25. 13C NMR spectrum of 1k (LASSBio-1615) (DMSO-d6, 50 MHz). Figura S26. Reverse phase HPLC spectrum of 1f (LASSBio-1463) (acetonitrile:water (60∶40)). Table S1. p38α MAPK inhibitory activity of compounds (1a–c, 1 k) at 10 µM. (ZIP) [file pone.0091660.s001.zip › Figure S9.tiff]

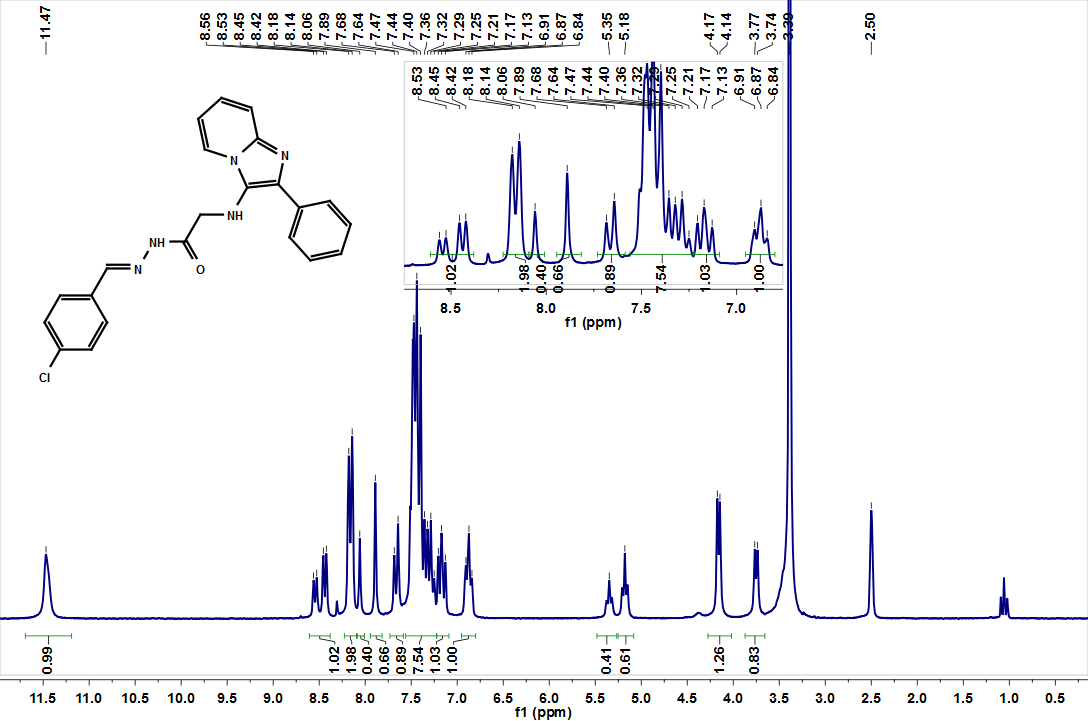

Supplement: File S1 — Figures S1–S26 and Table S1. Figure S1. 1H NMR spectrum of 1a (LASSBio-1507) (DMSO-d6, 200 MHz). Figure S2. 13C NMR spectrum of 1a (LASSBio-1507) (DMSO-d6, 50 MHz). Figure S3. 1H NMR spectrum of 1b (LASSBio-1616) (DMSO-d6, 200 MHz). Figure S4. 1H NMR spectrum of 1b (LASSBio-1616) (CDCl3, 200 MHz). Figure S5. 13C NMR spectrum of 1b (LASSBio-1616) (CDCl3, 50 MHz). Figure S6. 1H NMR spectrum of 1c (LASSBio-1535) (DMSO-d6, 200 MHz). Figure S7. 13C NMR spectrum of 1c (LASSBio-1535) (DMSO-d6, 50 MHz). Figure S8. 1H NMR spectrum of 1d (LASSBio-1695) (DMSO-d6, 200 MHz). Figure S9. 13C NMR spectrum of 1d (LASSBio-1695) (DMSO-d6, 50 MHz). Figure S10. 1H NMR spectrum of 1e (LASSBio-1696) (DMSO-d6, 200 MHz). Figure S11. 13C NMR spectrum of 1e (LASSBio-1696) (DMSO-d6, 50 MHz). Figure S12. 1H NMR spectrum of 1f (LASSBio-1463) (DMSO-d6, 300 MHz). Figure S13. 1H NMR spectrum of 1f (LASSBio-1463) (DMSO-d6, 300 MHz, T∼90°C). Figure S14. 13C NMR spectrum of 1f (LASSBio-1463) (DMSO-d6, 50 MHz). Figure S15. 1H NMR spectrum of 1g (LASSBio-1626) (DMSO-d6, 300 MHz). Figure S16. 1H NMR spectrum of 1g (LASSBio-1626) (DMSO-d6, 300 MHz, T∼90°C). Figure S17. 13C NMR spectrum of 1g (LASSBio-1626) (DMSO-d6, 50 MHz). Figure S18. 1H NMR spectrum of 1h (LASSBio-1697) (DMSO-d6, 200 MHz). Figure S19. 13C NMR spectrum of 1h (LASSBio-1697) (DMSO-d6, 50 MHz). Figure S20. 1H NMR spectrum of 1i (LASSBio-1749) (DMSO-d6, 200 MHz). Figure S21. 13C NMR spectrum of 1i (LASSBio-1749) (DMSO-d6, 50 MHz). Figure S22. 1H NMR spectrum of 1j (LASSBio-1698) (DMSO-d6, 200 MHz). Figure S23. 13C NMR spectrum of 1j (LASSBio-1698) (DMSO-d6, 50 MHz). Figura S24. 1H NMR spectrum of 1k (LASSBio-1615) (DMSO-d6, 200 MHz). Figura S25. 13C NMR spectrum of 1k (LASSBio-1615) (DMSO-d6, 50 MHz). Figura S26. Reverse phase HPLC spectrum of 1f (LASSBio-1463) (acetonitrile:water (60∶40)). Table S1. p38α MAPK inhibitory activity of compounds (1a–c, 1 k) at 10 µM. (ZIP) [file pone.0091660.s001.zip › Figure S10.tiff]

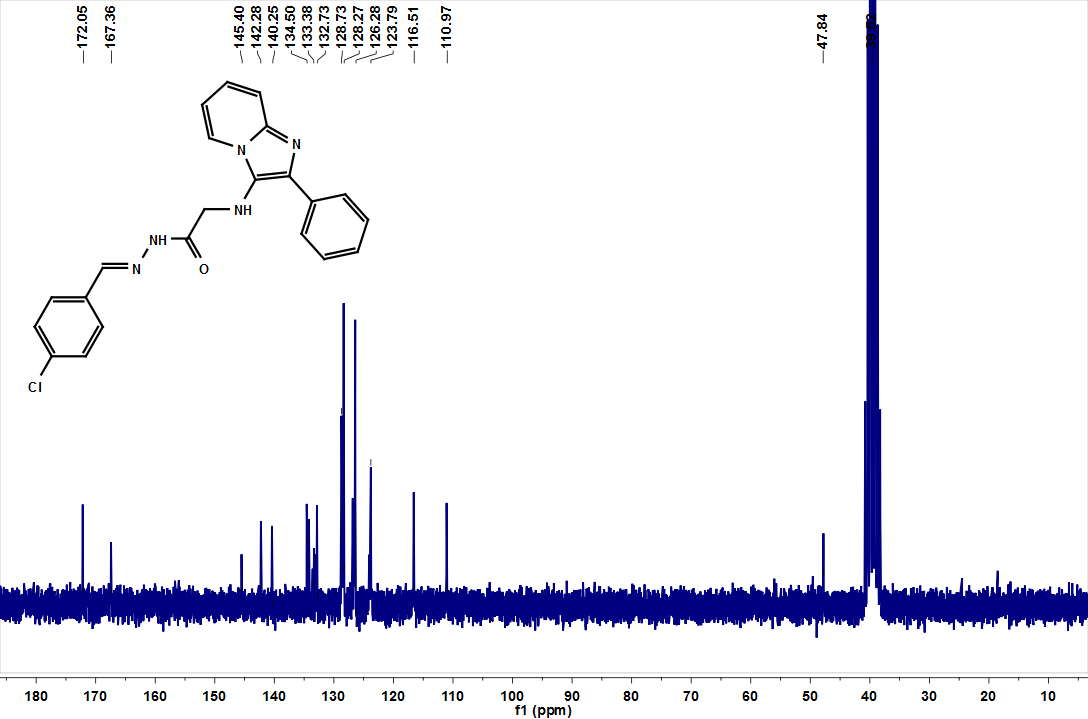

Supplement: File S1 — Figures S1–S26 and Table S1. Figure S1. 1H NMR spectrum of 1a (LASSBio-1507) (DMSO-d6, 200 MHz). Figure S2. 13C NMR spectrum of 1a (LASSBio-1507) (DMSO-d6, 50 MHz). Figure S3. 1H NMR spectrum of 1b (LASSBio-1616) (DMSO-d6, 200 MHz). Figure S4. 1H NMR spectrum of 1b (LASSBio-1616) (CDCl3, 200 MHz). Figure S5. 13C NMR spectrum of 1b (LASSBio-1616) (CDCl3, 50 MHz). Figure S6. 1H NMR spectrum of 1c (LASSBio-1535) (DMSO-d6, 200 MHz). Figure S7. 13C NMR spectrum of 1c (LASSBio-1535) (DMSO-d6, 50 MHz). Figure S8. 1H NMR spectrum of 1d (LASSBio-1695) (DMSO-d6, 200 MHz). Figure S9. 13C NMR spectrum of 1d (LASSBio-1695) (DMSO-d6, 50 MHz). Figure S10. 1H NMR spectrum of 1e (LASSBio-1696) (DMSO-d6, 200 MHz). Figure S11. 13C NMR spectrum of 1e (LASSBio-1696) (DMSO-d6, 50 MHz). Figure S12. 1H NMR spectrum of 1f (LASSBio-1463) (DMSO-d6, 300 MHz). Figure S13. 1H NMR spectrum of 1f (LASSBio-1463) (DMSO-d6, 300 MHz, T∼90°C). Figure S14. 13C NMR spectrum of 1f (LASSBio-1463) (DMSO-d6, 50 MHz). Figure S15. 1H NMR spectrum of 1g (LASSBio-1626) (DMSO-d6, 300 MHz). Figure S16. 1H NMR spectrum of 1g (LASSBio-1626) (DMSO-d6, 300 MHz, T∼90°C). Figure S17. 13C NMR spectrum of 1g (LASSBio-1626) (DMSO-d6, 50 MHz). Figure S18. 1H NMR spectrum of 1h (LASSBio-1697) (DMSO-d6, 200 MHz). Figure S19. 13C NMR spectrum of 1h (LASSBio-1697) (DMSO-d6, 50 MHz). Figure S20. 1H NMR spectrum of 1i (LASSBio-1749) (DMSO-d6, 200 MHz). Figure S21. 13C NMR spectrum of 1i (LASSBio-1749) (DMSO-d6, 50 MHz). Figure S22. 1H NMR spectrum of 1j (LASSBio-1698) (DMSO-d6, 200 MHz). Figure S23. 13C NMR spectrum of 1j (LASSBio-1698) (DMSO-d6, 50 MHz). Figura S24. 1H NMR spectrum of 1k (LASSBio-1615) (DMSO-d6, 200 MHz). Figura S25. 13C NMR spectrum of 1k (LASSBio-1615) (DMSO-d6, 50 MHz). Figura S26. Reverse phase HPLC spectrum of 1f (LASSBio-1463) (acetonitrile:water (60∶40)). Table S1. p38α MAPK inhibitory activity of compounds (1a–c, 1 k) at 10 µM. (ZIP) [file pone.0091660.s001.zip › Figure S11.tiff]

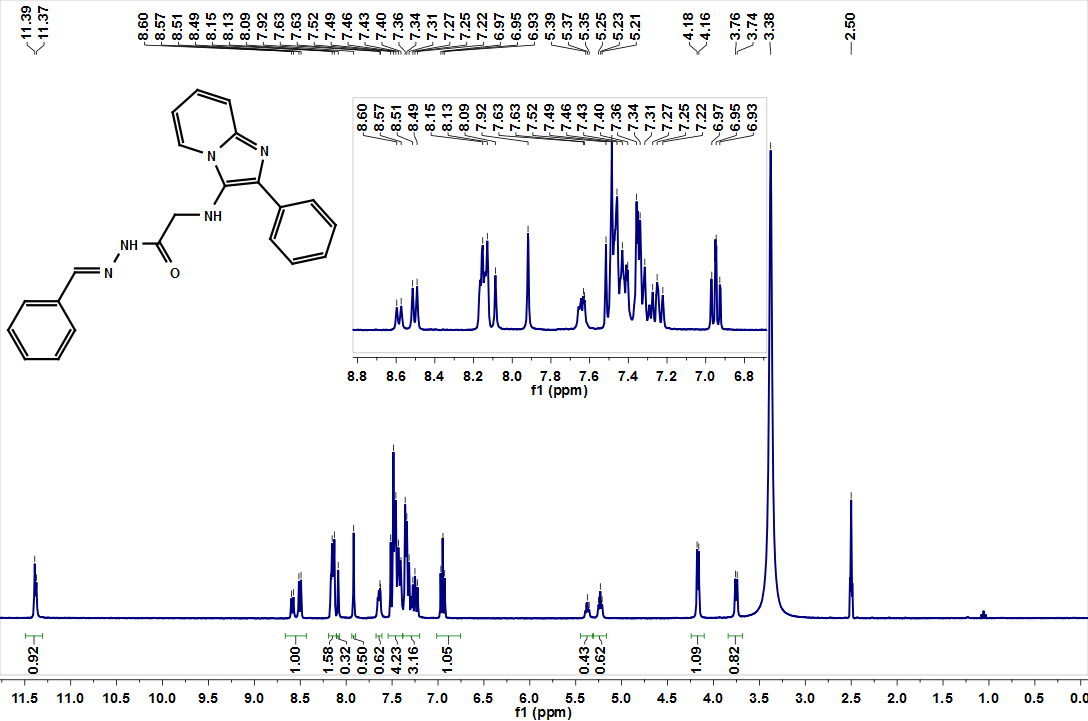

Supplement: File S1 — Figures S1–S26 and Table S1. Figure S1. 1H NMR spectrum of 1a (LASSBio-1507) (DMSO-d6, 200 MHz). Figure S2. 13C NMR spectrum of 1a (LASSBio-1507) (DMSO-d6, 50 MHz). Figure S3. 1H NMR spectrum of 1b (LASSBio-1616) (DMSO-d6, 200 MHz). Figure S4. 1H NMR spectrum of 1b (LASSBio-1616) (CDCl3, 200 MHz). Figure S5. 13C NMR spectrum of 1b (LASSBio-1616) (CDCl3, 50 MHz). Figure S6. 1H NMR spectrum of 1c (LASSBio-1535) (DMSO-d6, 200 MHz). Figure S7. 13C NMR spectrum of 1c (LASSBio-1535) (DMSO-d6, 50 MHz). Figure S8. 1H NMR spectrum of 1d (LASSBio-1695) (DMSO-d6, 200 MHz). Figure S9. 13C NMR spectrum of 1d (LASSBio-1695) (DMSO-d6, 50 MHz). Figure S10. 1H NMR spectrum of 1e (LASSBio-1696) (DMSO-d6, 200 MHz). Figure S11. 13C NMR spectrum of 1e (LASSBio-1696) (DMSO-d6, 50 MHz). Figure S12. 1H NMR spectrum of 1f (LASSBio-1463) (DMSO-d6, 300 MHz). Figure S13. 1H NMR spectrum of 1f (LASSBio-1463) (DMSO-d6, 300 MHz, T∼90°C). Figure S14. 13C NMR spectrum of 1f (LASSBio-1463) (DMSO-d6, 50 MHz). Figure S15. 1H NMR spectrum of 1g (LASSBio-1626) (DMSO-d6, 300 MHz). Figure S16. 1H NMR spectrum of 1g (LASSBio-1626) (DMSO-d6, 300 MHz, T∼90°C). Figure S17. 13C NMR spectrum of 1g (LASSBio-1626) (DMSO-d6, 50 MHz). Figure S18. 1H NMR spectrum of 1h (LASSBio-1697) (DMSO-d6, 200 MHz). Figure S19. 13C NMR spectrum of 1h (LASSBio-1697) (DMSO-d6, 50 MHz). Figure S20. 1H NMR spectrum of 1i (LASSBio-1749) (DMSO-d6, 200 MHz). Figure S21. 13C NMR spectrum of 1i (LASSBio-1749) (DMSO-d6, 50 MHz). Figure S22. 1H NMR spectrum of 1j (LASSBio-1698) (DMSO-d6, 200 MHz). Figure S23. 13C NMR spectrum of 1j (LASSBio-1698) (DMSO-d6, 50 MHz). Figura S24. 1H NMR spectrum of 1k (LASSBio-1615) (DMSO-d6, 200 MHz). Figura S25. 13C NMR spectrum of 1k (LASSBio-1615) (DMSO-d6, 50 MHz). Figura S26. Reverse phase HPLC spectrum of 1f (LASSBio-1463) (acetonitrile:water (60∶40)). Table S1. p38α MAPK inhibitory activity of compounds (1a–c, 1 k) at 10 µM. (ZIP) [file pone.0091660.s001.zip › Figure S12.tiff]

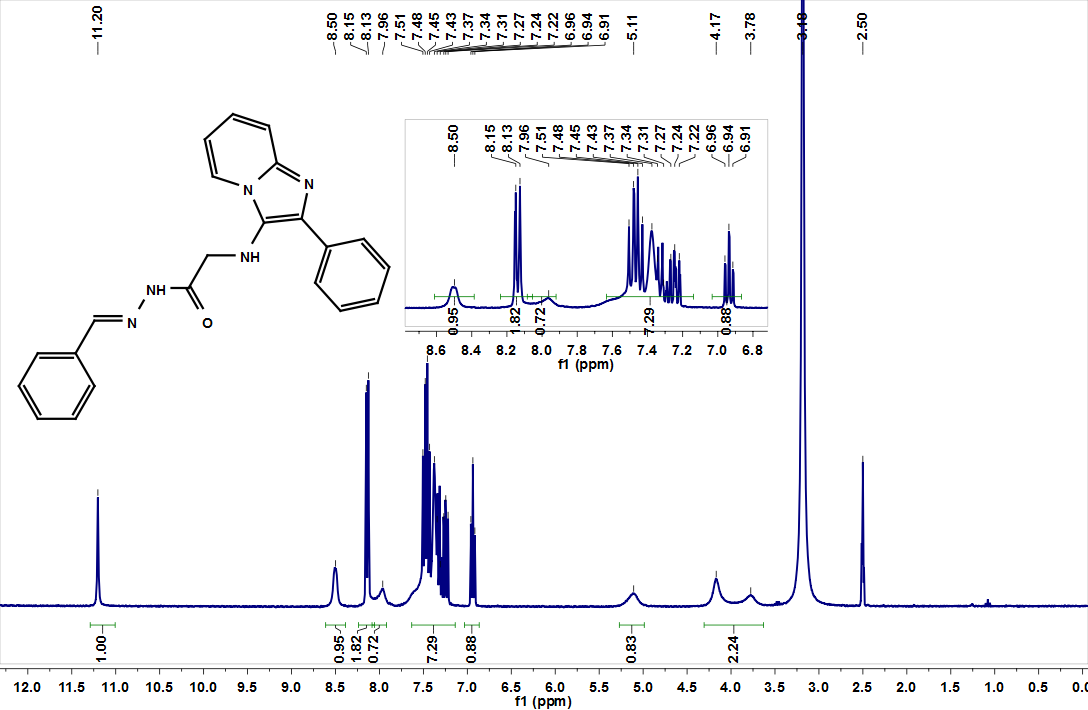

Supplement: File S1 — Figures S1–S26 and Table S1. Figure S1. 1H NMR spectrum of 1a (LASSBio-1507) (DMSO-d6, 200 MHz). Figure S2. 13C NMR spectrum of 1a (LASSBio-1507) (DMSO-d6, 50 MHz). Figure S3. 1H NMR spectrum of 1b (LASSBio-1616) (DMSO-d6, 200 MHz). Figure S4. 1H NMR spectrum of 1b (LASSBio-1616) (CDCl3, 200 MHz). Figure S5. 13C NMR spectrum of 1b (LASSBio-1616) (CDCl3, 50 MHz). Figure S6. 1H NMR spectrum of 1c (LASSBio-1535) (DMSO-d6, 200 MHz). Figure S7. 13C NMR spectrum of 1c (LASSBio-1535) (DMSO-d6, 50 MHz). Figure S8. 1H NMR spectrum of 1d (LASSBio-1695) (DMSO-d6, 200 MHz). Figure S9. 13C NMR spectrum of 1d (LASSBio-1695) (DMSO-d6, 50 MHz). Figure S10. 1H NMR spectrum of 1e (LASSBio-1696) (DMSO-d6, 200 MHz). Figure S11. 13C NMR spectrum of 1e (LASSBio-1696) (DMSO-d6, 50 MHz). Figure S12. 1H NMR spectrum of 1f (LASSBio-1463) (DMSO-d6, 300 MHz). Figure S13. 1H NMR spectrum of 1f (LASSBio-1463) (DMSO-d6, 300 MHz, T∼90°C). Figure S14. 13C NMR spectrum of 1f (LASSBio-1463) (DMSO-d6, 50 MHz). Figure S15. 1H NMR spectrum of 1g (LASSBio-1626) (DMSO-d6, 300 MHz). Figure S16. 1H NMR spectrum of 1g (LASSBio-1626) (DMSO-d6, 300 MHz, T∼90°C). Figure S17. 13C NMR spectrum of 1g (LASSBio-1626) (DMSO-d6, 50 MHz). Figure S18. 1H NMR spectrum of 1h (LASSBio-1697) (DMSO-d6, 200 MHz). Figure S19. 13C NMR spectrum of 1h (LASSBio-1697) (DMSO-d6, 50 MHz). Figure S20. 1H NMR spectrum of 1i (LASSBio-1749) (DMSO-d6, 200 MHz). Figure S21. 13C NMR spectrum of 1i (LASSBio-1749) (DMSO-d6, 50 MHz). Figure S22. 1H NMR spectrum of 1j (LASSBio-1698) (DMSO-d6, 200 MHz). Figure S23. 13C NMR spectrum of 1j (LASSBio-1698) (DMSO-d6, 50 MHz). Figura S24. 1H NMR spectrum of 1k (LASSBio-1615) (DMSO-d6, 200 MHz). Figura S25. 13C NMR spectrum of 1k (LASSBio-1615) (DMSO-d6, 50 MHz). Figura S26. Reverse phase HPLC spectrum of 1f (LASSBio-1463) (acetonitrile:water (60∶40)). Table S1. p38α MAPK inhibitory activity of compounds (1a–c, 1 k) at 10 µM. (ZIP) [file pone.0091660.s001.zip › Figure S13.tiff]

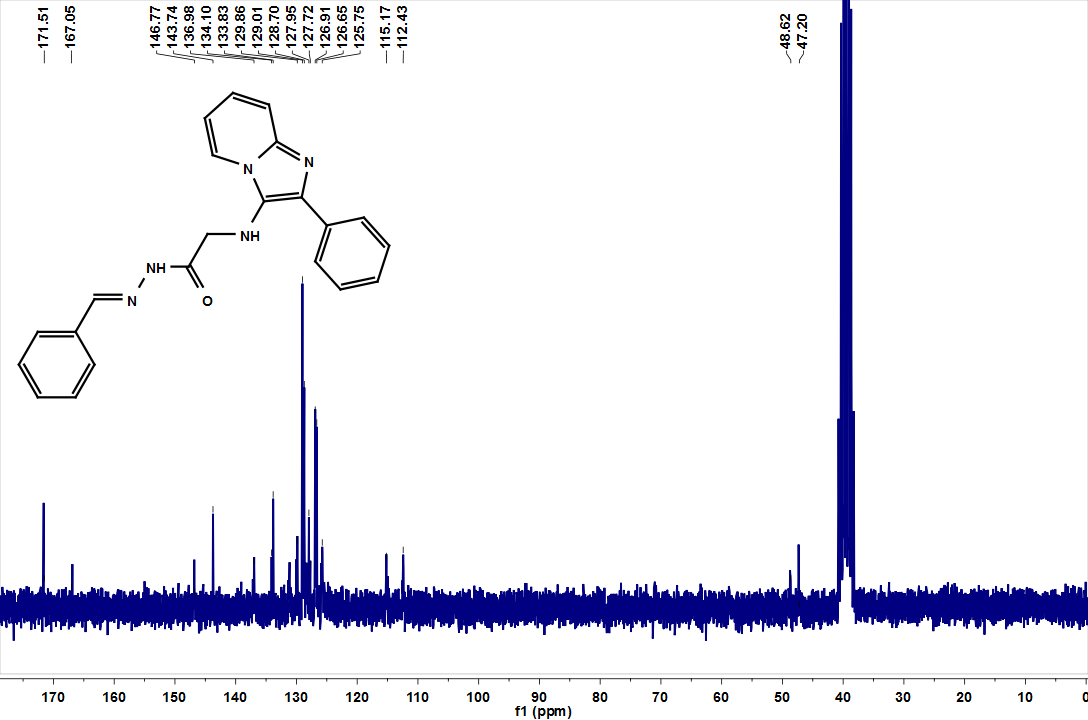

Supplement: File S1 — Figures S1–S26 and Table S1. Figure S1. 1H NMR spectrum of 1a (LASSBio-1507) (DMSO-d6, 200 MHz). Figure S2. 13C NMR spectrum of 1a (LASSBio-1507) (DMSO-d6, 50 MHz). Figure S3. 1H NMR spectrum of 1b (LASSBio-1616) (DMSO-d6, 200 MHz). Figure S4. 1H NMR spectrum of 1b (LASSBio-1616) (CDCl3, 200 MHz). Figure S5. 13C NMR spectrum of 1b (LASSBio-1616) (CDCl3, 50 MHz). Figure S6. 1H NMR spectrum of 1c (LASSBio-1535) (DMSO-d6, 200 MHz). Figure S7. 13C NMR spectrum of 1c (LASSBio-1535) (DMSO-d6, 50 MHz). Figure S8. 1H NMR spectrum of 1d (LASSBio-1695) (DMSO-d6, 200 MHz). Figure S9. 13C NMR spectrum of 1d (LASSBio-1695) (DMSO-d6, 50 MHz). Figure S10. 1H NMR spectrum of 1e (LASSBio-1696) (DMSO-d6, 200 MHz). Figure S11. 13C NMR spectrum of 1e (LASSBio-1696) (DMSO-d6, 50 MHz). Figure S12. 1H NMR spectrum of 1f (LASSBio-1463) (DMSO-d6, 300 MHz). Figure S13. 1H NMR spectrum of 1f (LASSBio-1463) (DMSO-d6, 300 MHz, T∼90°C). Figure S14. 13C NMR spectrum of 1f (LASSBio-1463) (DMSO-d6, 50 MHz). Figure S15. 1H NMR spectrum of 1g (LASSBio-1626) (DMSO-d6, 300 MHz). Figure S16. 1H NMR spectrum of 1g (LASSBio-1626) (DMSO-d6, 300 MHz, T∼90°C). Figure S17. 13C NMR spectrum of 1g (LASSBio-1626) (DMSO-d6, 50 MHz). Figure S18. 1H NMR spectrum of 1h (LASSBio-1697) (DMSO-d6, 200 MHz). Figure S19. 13C NMR spectrum of 1h (LASSBio-1697) (DMSO-d6, 50 MHz). Figure S20. 1H NMR spectrum of 1i (LASSBio-1749) (DMSO-d6, 200 MHz). Figure S21. 13C NMR spectrum of 1i (LASSBio-1749) (DMSO-d6, 50 MHz). Figure S22. 1H NMR spectrum of 1j (LASSBio-1698) (DMSO-d6, 200 MHz). Figure S23. 13C NMR spectrum of 1j (LASSBio-1698) (DMSO-d6, 50 MHz). Figura S24. 1H NMR spectrum of 1k (LASSBio-1615) (DMSO-d6, 200 MHz). Figura S25. 13C NMR spectrum of 1k (LASSBio-1615) (DMSO-d6, 50 MHz). Figura S26. Reverse phase HPLC spectrum of 1f (LASSBio-1463) (acetonitrile:water (60∶40)). Table S1. p38α MAPK inhibitory activity of compounds (1a–c, 1 k) at 10 µM. (ZIP) [file pone.0091660.s001.zip › Figure S14.tiff]

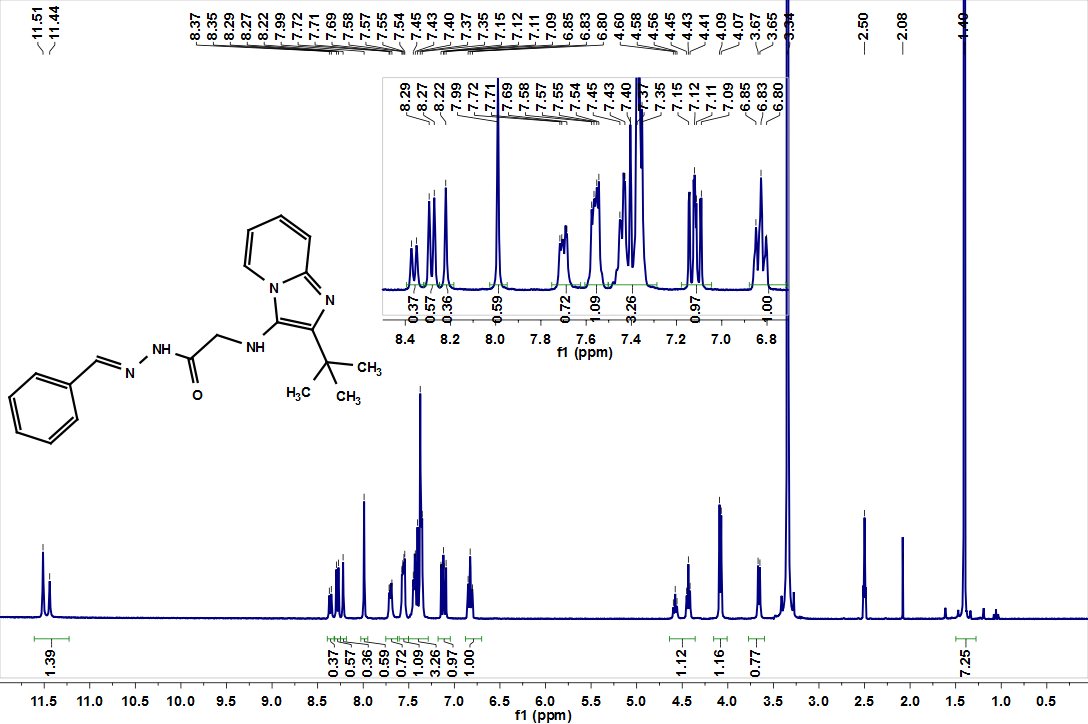

Supplement: File S1 — Figures S1–S26 and Table S1. Figure S1. 1H NMR spectrum of 1a (LASSBio-1507) (DMSO-d6, 200 MHz). Figure S2. 13C NMR spectrum of 1a (LASSBio-1507) (DMSO-d6, 50 MHz). Figure S3. 1H NMR spectrum of 1b (LASSBio-1616) (DMSO-d6, 200 MHz). Figure S4. 1H NMR spectrum of 1b (LASSBio-1616) (CDCl3, 200 MHz). Figure S5. 13C NMR spectrum of 1b (LASSBio-1616) (CDCl3, 50 MHz). Figure S6. 1H NMR spectrum of 1c (LASSBio-1535) (DMSO-d6, 200 MHz). Figure S7. 13C NMR spectrum of 1c (LASSBio-1535) (DMSO-d6, 50 MHz). Figure S8. 1H NMR spectrum of 1d (LASSBio-1695) (DMSO-d6, 200 MHz). Figure S9. 13C NMR spectrum of 1d (LASSBio-1695) (DMSO-d6, 50 MHz). Figure S10. 1H NMR spectrum of 1e (LASSBio-1696) (DMSO-d6, 200 MHz). Figure S11. 13C NMR spectrum of 1e (LASSBio-1696) (DMSO-d6, 50 MHz). Figure S12. 1H NMR spectrum of 1f (LASSBio-1463) (DMSO-d6, 300 MHz). Figure S13. 1H NMR spectrum of 1f (LASSBio-1463) (DMSO-d6, 300 MHz, T∼90°C). Figure S14. 13C NMR spectrum of 1f (LASSBio-1463) (DMSO-d6, 50 MHz). Figure S15. 1H NMR spectrum of 1g (LASSBio-1626) (DMSO-d6, 300 MHz). Figure S16. 1H NMR spectrum of 1g (LASSBio-1626) (DMSO-d6, 300 MHz, T∼90°C). Figure S17. 13C NMR spectrum of 1g (LASSBio-1626) (DMSO-d6, 50 MHz). Figure S18. 1H NMR spectrum of 1h (LASSBio-1697) (DMSO-d6, 200 MHz). Figure S19. 13C NMR spectrum of 1h (LASSBio-1697) (DMSO-d6, 50 MHz). Figure S20. 1H NMR spectrum of 1i (LASSBio-1749) (DMSO-d6, 200 MHz). Figure S21. 13C NMR spectrum of 1i (LASSBio-1749) (DMSO-d6, 50 MHz). Figure S22. 1H NMR spectrum of 1j (LASSBio-1698) (DMSO-d6, 200 MHz). Figure S23. 13C NMR spectrum of 1j (LASSBio-1698) (DMSO-d6, 50 MHz). Figura S24. 1H NMR spectrum of 1k (LASSBio-1615) (DMSO-d6, 200 MHz). Figura S25. 13C NMR spectrum of 1k (LASSBio-1615) (DMSO-d6, 50 MHz). Figura S26. Reverse phase HPLC spectrum of 1f (LASSBio-1463) (acetonitrile:water (60∶40)). Table S1. p38α MAPK inhibitory activity of compounds (1a–c, 1 k) at 10 µM. (ZIP) [file pone.0091660.s001.zip › Figure S15.tiff]

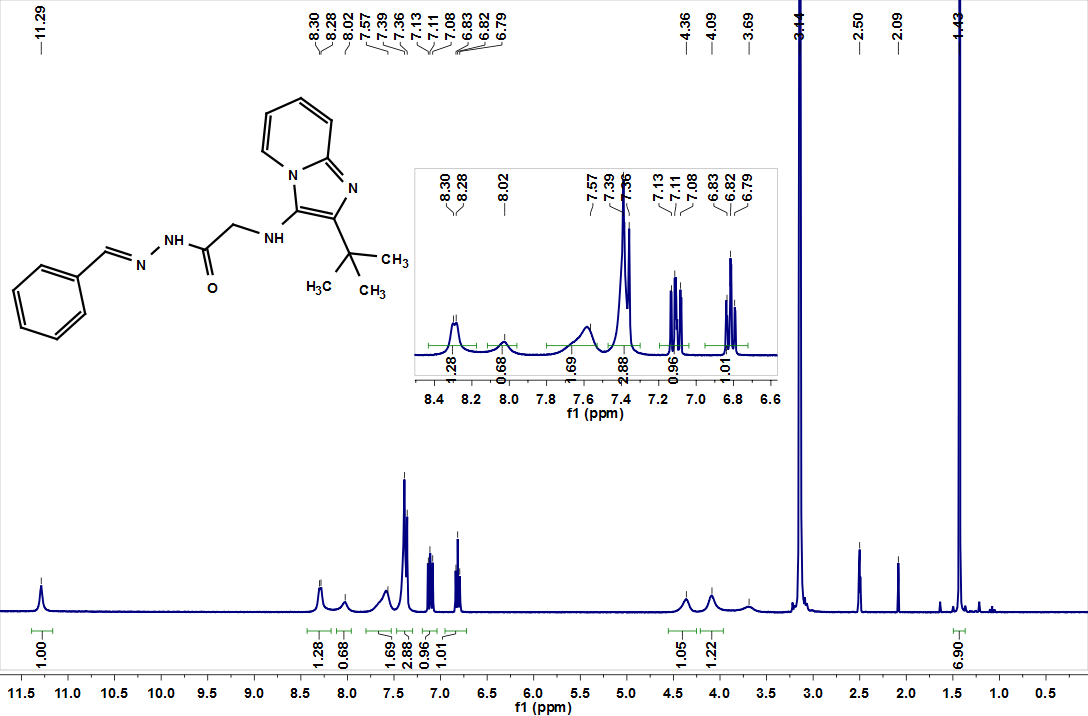

Supplement: File S1 — Figures S1–S26 and Table S1. Figure S1. 1H NMR spectrum of 1a (LASSBio-1507) (DMSO-d6, 200 MHz). Figure S2. 13C NMR spectrum of 1a (LASSBio-1507) (DMSO-d6, 50 MHz). Figure S3. 1H NMR spectrum of 1b (LASSBio-1616) (DMSO-d6, 200 MHz). Figure S4. 1H NMR spectrum of 1b (LASSBio-1616) (CDCl3, 200 MHz). Figure S5. 13C NMR spectrum of 1b (LASSBio-1616) (CDCl3, 50 MHz). Figure S6. 1H NMR spectrum of 1c (LASSBio-1535) (DMSO-d6, 200 MHz). Figure S7. 13C NMR spectrum of 1c (LASSBio-1535) (DMSO-d6, 50 MHz). Figure S8. 1H NMR spectrum of 1d (LASSBio-1695) (DMSO-d6, 200 MHz). Figure S9. 13C NMR spectrum of 1d (LASSBio-1695) (DMSO-d6, 50 MHz). Figure S10. 1H NMR spectrum of 1e (LASSBio-1696) (DMSO-d6, 200 MHz). Figure S11. 13C NMR spectrum of 1e (LASSBio-1696) (DMSO-d6, 50 MHz). Figure S12. 1H NMR spectrum of 1f (LASSBio-1463) (DMSO-d6, 300 MHz). Figure S13. 1H NMR spectrum of 1f (LASSBio-1463) (DMSO-d6, 300 MHz, T∼90°C). Figure S14. 13C NMR spectrum of 1f (LASSBio-1463) (DMSO-d6, 50 MHz). Figure S15. 1H NMR spectrum of 1g (LASSBio-1626) (DMSO-d6, 300 MHz). Figure S16. 1H NMR spectrum of 1g (LASSBio-1626) (DMSO-d6, 300 MHz, T∼90°C). Figure S17. 13C NMR spectrum of 1g (LASSBio-1626) (DMSO-d6, 50 MHz). Figure S18. 1H NMR spectrum of 1h (LASSBio-1697) (DMSO-d6, 200 MHz). Figure S19. 13C NMR spectrum of 1h (LASSBio-1697) (DMSO-d6, 50 MHz). Figure S20. 1H NMR spectrum of 1i (LASSBio-1749) (DMSO-d6, 200 MHz). Figure S21. 13C NMR spectrum of 1i (LASSBio-1749) (DMSO-d6, 50 MHz). Figure S22. 1H NMR spectrum of 1j (LASSBio-1698) (DMSO-d6, 200 MHz). Figure S23. 13C NMR spectrum of 1j (LASSBio-1698) (DMSO-d6, 50 MHz). Figura S24. 1H NMR spectrum of 1k (LASSBio-1615) (DMSO-d6, 200 MHz). Figura S25. 13C NMR spectrum of 1k (LASSBio-1615) (DMSO-d6, 50 MHz). Figura S26. Reverse phase HPLC spectrum of 1f (LASSBio-1463) (acetonitrile:water (60∶40)). Table S1. p38α MAPK inhibitory activity of compounds (1a–c, 1 k) at 10 µM. (ZIP) [file pone.0091660.s001.zip › Figure S16.tiff]

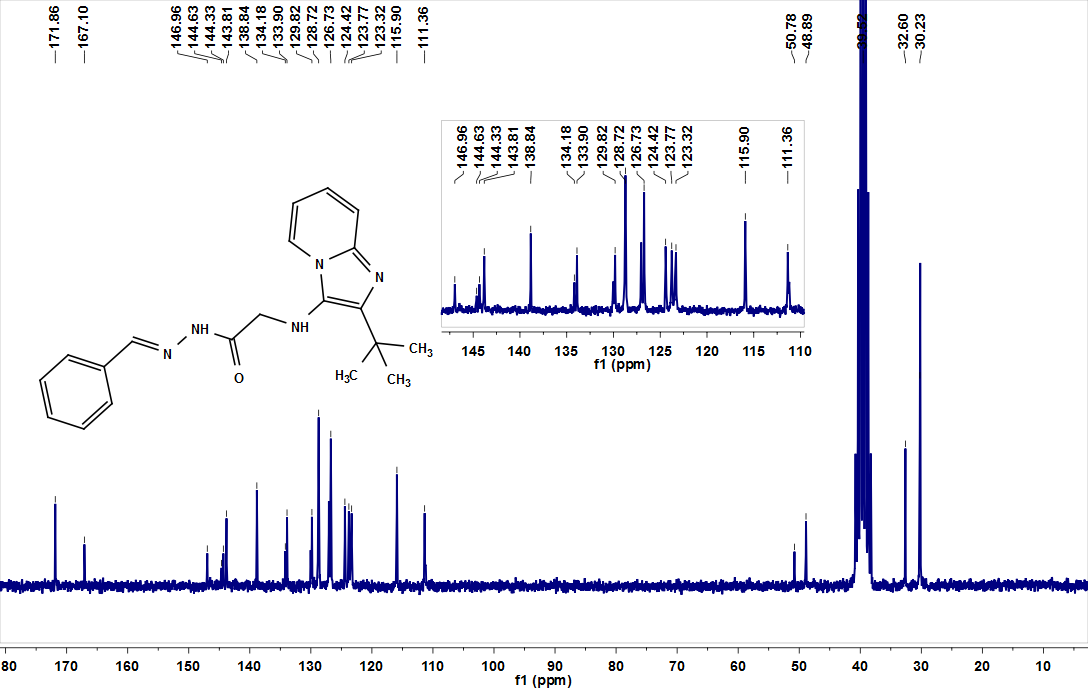

Supplement: File S1 — Figures S1–S26 and Table S1. Figure S1. 1H NMR spectrum of 1a (LASSBio-1507) (DMSO-d6, 200 MHz). Figure S2. 13C NMR spectrum of 1a (LASSBio-1507) (DMSO-d6, 50 MHz). Figure S3. 1H NMR spectrum of 1b (LASSBio-1616) (DMSO-d6, 200 MHz). Figure S4. 1H NMR spectrum of 1b (LASSBio-1616) (CDCl3, 200 MHz). Figure S5. 13C NMR spectrum of 1b (LASSBio-1616) (CDCl3, 50 MHz). Figure S6. 1H NMR spectrum of 1c (LASSBio-1535) (DMSO-d6, 200 MHz). Figure S7. 13C NMR spectrum of 1c (LASSBio-1535) (DMSO-d6, 50 MHz). Figure S8. 1H NMR spectrum of 1d (LASSBio-1695) (DMSO-d6, 200 MHz). Figure S9. 13C NMR spectrum of 1d (LASSBio-1695) (DMSO-d6, 50 MHz). Figure S10. 1H NMR spectrum of 1e (LASSBio-1696) (DMSO-d6, 200 MHz). Figure S11. 13C NMR spectrum of 1e (LASSBio-1696) (DMSO-d6, 50 MHz). Figure S12. 1H NMR spectrum of 1f (LASSBio-1463) (DMSO-d6, 300 MHz). Figure S13. 1H NMR spectrum of 1f (LASSBio-1463) (DMSO-d6, 300 MHz, T∼90°C). Figure S14. 13C NMR spectrum of 1f (LASSBio-1463) (DMSO-d6, 50 MHz). Figure S15. 1H NMR spectrum of 1g (LASSBio-1626) (DMSO-d6, 300 MHz). Figure S16. 1H NMR spectrum of 1g (LASSBio-1626) (DMSO-d6, 300 MHz, T∼90°C). Figure S17. 13C NMR spectrum of 1g (LASSBio-1626) (DMSO-d6, 50 MHz). Figure S18. 1H NMR spectrum of 1h (LASSBio-1697) (DMSO-d6, 200 MHz). Figure S19. 13C NMR spectrum of 1h (LASSBio-1697) (DMSO-d6, 50 MHz). Figure S20. 1H NMR spectrum of 1i (LASSBio-1749) (DMSO-d6, 200 MHz). Figure S21. 13C NMR spectrum of 1i (LASSBio-1749) (DMSO-d6, 50 MHz). Figure S22. 1H NMR spectrum of 1j (LASSBio-1698) (DMSO-d6, 200 MHz). Figure S23. 13C NMR spectrum of 1j (LASSBio-1698) (DMSO-d6, 50 MHz). Figura S24. 1H NMR spectrum of 1k (LASSBio-1615) (DMSO-d6, 200 MHz). Figura S25. 13C NMR spectrum of 1k (LASSBio-1615) (DMSO-d6, 50 MHz). Figura S26. Reverse phase HPLC spectrum of 1f (LASSBio-1463) (acetonitrile:water (60∶40)). Table S1. p38α MAPK inhibitory activity of compounds (1a–c, 1 k) at 10 µM. (ZIP) [file pone.0091660.s001.zip › Figure S17.tiff]

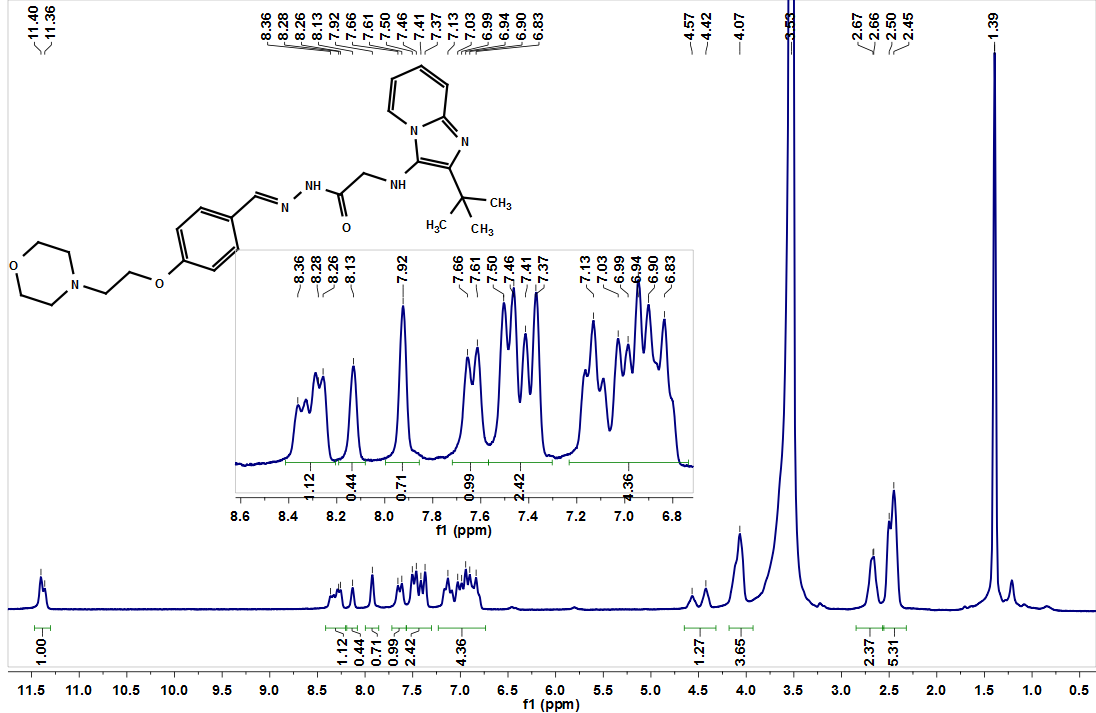

Supplement: File S1 — Figures S1–S26 and Table S1. Figure S1. 1H NMR spectrum of 1a (LASSBio-1507) (DMSO-d6, 200 MHz). Figure S2. 13C NMR spectrum of 1a (LASSBio-1507) (DMSO-d6, 50 MHz). Figure S3. 1H NMR spectrum of 1b (LASSBio-1616) (DMSO-d6, 200 MHz). Figure S4. 1H NMR spectrum of 1b (LASSBio-1616) (CDCl3, 200 MHz). Figure S5. 13C NMR spectrum of 1b (LASSBio-1616) (CDCl3, 50 MHz). Figure S6. 1H NMR spectrum of 1c (LASSBio-1535) (DMSO-d6, 200 MHz). Figure S7. 13C NMR spectrum of 1c (LASSBio-1535) (DMSO-d6, 50 MHz). Figure S8. 1H NMR spectrum of 1d (LASSBio-1695) (DMSO-d6, 200 MHz). Figure S9. 13C NMR spectrum of 1d (LASSBio-1695) (DMSO-d6, 50 MHz). Figure S10. 1H NMR spectrum of 1e (LASSBio-1696) (DMSO-d6, 200 MHz). Figure S11. 13C NMR spectrum of 1e (LASSBio-1696) (DMSO-d6, 50 MHz). Figure S12. 1H NMR spectrum of 1f (LASSBio-1463) (DMSO-d6, 300 MHz). Figure S13. 1H NMR spectrum of 1f (LASSBio-1463) (DMSO-d6, 300 MHz, T∼90°C). Figure S14. 13C NMR spectrum of 1f (LASSBio-1463) (DMSO-d6, 50 MHz). Figure S15. 1H NMR spectrum of 1g (LASSBio-1626) (DMSO-d6, 300 MHz). Figure S16. 1H NMR spectrum of 1g (LASSBio-1626) (DMSO-d6, 300 MHz, T∼90°C). Figure S17. 13C NMR spectrum of 1g (LASSBio-1626) (DMSO-d6, 50 MHz). Figure S18. 1H NMR spectrum of 1h (LASSBio-1697) (DMSO-d6, 200 MHz). Figure S19. 13C NMR spectrum of 1h (LASSBio-1697) (DMSO-d6, 50 MHz). Figure S20. 1H NMR spectrum of 1i (LASSBio-1749) (DMSO-d6, 200 MHz). Figure S21. 13C NMR spectrum of 1i (LASSBio-1749) (DMSO-d6, 50 MHz). Figure S22. 1H NMR spectrum of 1j (LASSBio-1698) (DMSO-d6, 200 MHz). Figure S23. 13C NMR spectrum of 1j (LASSBio-1698) (DMSO-d6, 50 MHz). Figura S24. 1H NMR spectrum of 1k (LASSBio-1615) (DMSO-d6, 200 MHz). Figura S25. 13C NMR spectrum of 1k (LASSBio-1615) (DMSO-d6, 50 MHz). Figura S26. Reverse phase HPLC spectrum of 1f (LASSBio-1463) (acetonitrile:water (60∶40)). Table S1. p38α MAPK inhibitory activity of compounds (1a–c, 1 k) at 10 µM. (ZIP) [file pone.0091660.s001.zip › Figure S18.tiff]

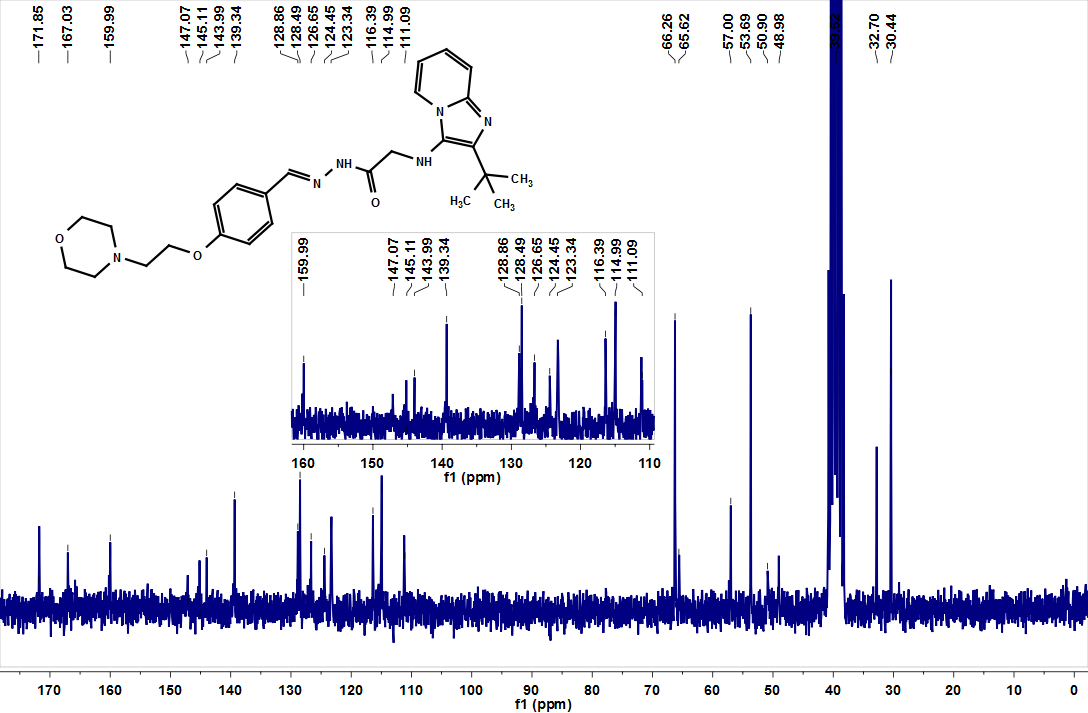

Supplement: File S1 — Figures S1–S26 and Table S1. Figure S1. 1H NMR spectrum of 1a (LASSBio-1507) (DMSO-d6, 200 MHz). Figure S2. 13C NMR spectrum of 1a (LASSBio-1507) (DMSO-d6, 50 MHz). Figure S3. 1H NMR spectrum of 1b (LASSBio-1616) (DMSO-d6, 200 MHz). Figure S4. 1H NMR spectrum of 1b (LASSBio-1616) (CDCl3, 200 MHz). Figure S5. 13C NMR spectrum of 1b (LASSBio-1616) (CDCl3, 50 MHz). Figure S6. 1H NMR spectrum of 1c (LASSBio-1535) (DMSO-d6, 200 MHz). Figure S7. 13C NMR spectrum of 1c (LASSBio-1535) (DMSO-d6, 50 MHz). Figure S8. 1H NMR spectrum of 1d (LASSBio-1695) (DMSO-d6, 200 MHz). Figure S9. 13C NMR spectrum of 1d (LASSBio-1695) (DMSO-d6, 50 MHz). Figure S10. 1H NMR spectrum of 1e (LASSBio-1696) (DMSO-d6, 200 MHz). Figure S11. 13C NMR spectrum of 1e (LASSBio-1696) (DMSO-d6, 50 MHz). Figure S12. 1H NMR spectrum of 1f (LASSBio-1463) (DMSO-d6, 300 MHz). Figure S13. 1H NMR spectrum of 1f (LASSBio-1463) (DMSO-d6, 300 MHz, T∼90°C). Figure S14. 13C NMR spectrum of 1f (LASSBio-1463) (DMSO-d6, 50 MHz). Figure S15. 1H NMR spectrum of 1g (LASSBio-1626) (DMSO-d6, 300 MHz). Figure S16. 1H NMR spectrum of 1g (LASSBio-1626) (DMSO-d6, 300 MHz, T∼90°C). Figure S17. 13C NMR spectrum of 1g (LASSBio-1626) (DMSO-d6, 50 MHz). Figure S18. 1H NMR spectrum of 1h (LASSBio-1697) (DMSO-d6, 200 MHz). Figure S19. 13C NMR spectrum of 1h (LASSBio-1697) (DMSO-d6, 50 MHz). Figure S20. 1H NMR spectrum of 1i (LASSBio-1749) (DMSO-d6, 200 MHz). Figure S21. 13C NMR spectrum of 1i (LASSBio-1749) (DMSO-d6, 50 MHz). Figure S22. 1H NMR spectrum of 1j (LASSBio-1698) (DMSO-d6, 200 MHz). Figure S23. 13C NMR spectrum of 1j (LASSBio-1698) (DMSO-d6, 50 MHz). Figura S24. 1H NMR spectrum of 1k (LASSBio-1615) (DMSO-d6, 200 MHz). Figura S25. 13C NMR spectrum of 1k (LASSBio-1615) (DMSO-d6, 50 MHz). Figura S26. Reverse phase HPLC spectrum of 1f (LASSBio-1463) (acetonitrile:water (60∶40)). Table S1. p38α MAPK inhibitory activity of compounds (1a–c, 1 k) at 10 µM. (ZIP) [file pone.0091660.s001.zip › Figure S19.tiff]

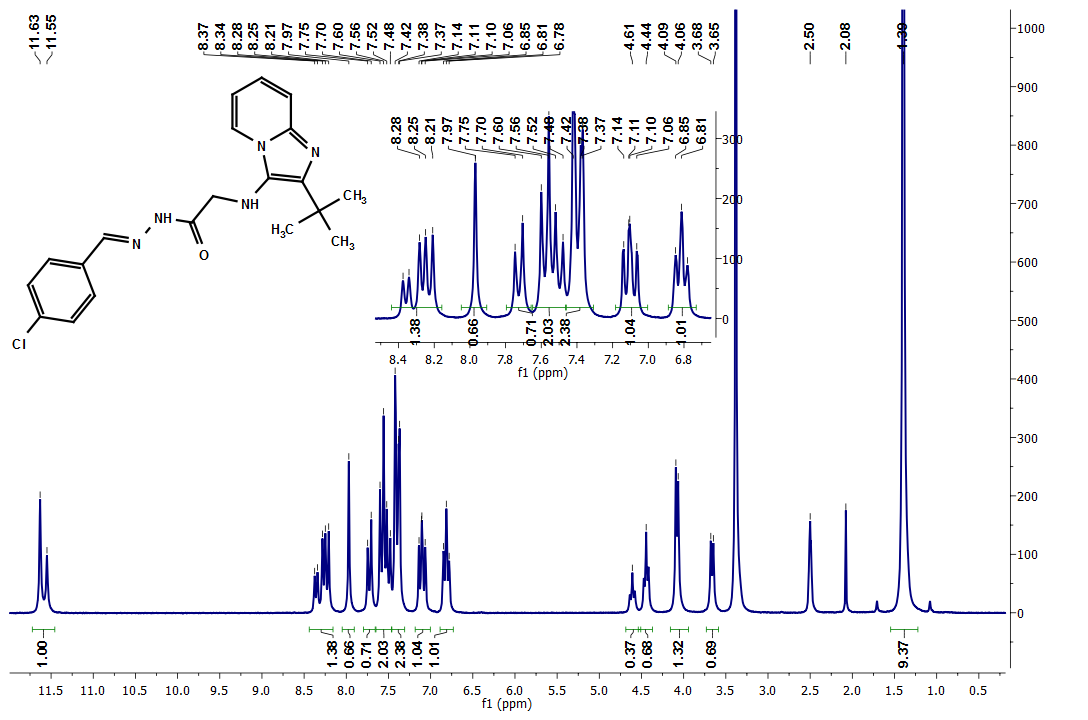

Supplement: File S1 — Figures S1–S26 and Table S1. Figure S1. 1H NMR spectrum of 1a (LASSBio-1507) (DMSO-d6, 200 MHz). Figure S2. 13C NMR spectrum of 1a (LASSBio-1507) (DMSO-d6, 50 MHz). Figure S3. 1H NMR spectrum of 1b (LASSBio-1616) (DMSO-d6, 200 MHz). Figure S4. 1H NMR spectrum of 1b (LASSBio-1616) (CDCl3, 200 MHz). Figure S5. 13C NMR spectrum of 1b (LASSBio-1616) (CDCl3, 50 MHz). Figure S6. 1H NMR spectrum of 1c (LASSBio-1535) (DMSO-d6, 200 MHz). Figure S7. 13C NMR spectrum of 1c (LASSBio-1535) (DMSO-d6, 50 MHz). Figure S8. 1H NMR spectrum of 1d (LASSBio-1695) (DMSO-d6, 200 MHz). Figure S9. 13C NMR spectrum of 1d (LASSBio-1695) (DMSO-d6, 50 MHz). Figure S10. 1H NMR spectrum of 1e (LASSBio-1696) (DMSO-d6, 200 MHz). Figure S11. 13C NMR spectrum of 1e (LASSBio-1696) (DMSO-d6, 50 MHz). Figure S12. 1H NMR spectrum of 1f (LASSBio-1463) (DMSO-d6, 300 MHz). Figure S13. 1H NMR spectrum of 1f (LASSBio-1463) (DMSO-d6, 300 MHz, T∼90°C). Figure S14. 13C NMR spectrum of 1f (LASSBio-1463) (DMSO-d6, 50 MHz). Figure S15. 1H NMR spectrum of 1g (LASSBio-1626) (DMSO-d6, 300 MHz). Figure S16. 1H NMR spectrum of 1g (LASSBio-1626) (DMSO-d6, 300 MHz, T∼90°C). Figure S17. 13C NMR spectrum of 1g (LASSBio-1626) (DMSO-d6, 50 MHz). Figure S18. 1H NMR spectrum of 1h (LASSBio-1697) (DMSO-d6, 200 MHz). Figure S19. 13C NMR spectrum of 1h (LASSBio-1697) (DMSO-d6, 50 MHz). Figure S20. 1H NMR spectrum of 1i (LASSBio-1749) (DMSO-d6, 200 MHz). Figure S21. 13C NMR spectrum of 1i (LASSBio-1749) (DMSO-d6, 50 MHz). Figure S22. 1H NMR spectrum of 1j (LASSBio-1698) (DMSO-d6, 200 MHz). Figure S23. 13C NMR spectrum of 1j (LASSBio-1698) (DMSO-d6, 50 MHz). Figura S24. 1H NMR spectrum of 1k (LASSBio-1615) (DMSO-d6, 200 MHz). Figura S25. 13C NMR spectrum of 1k (LASSBio-1615) (DMSO-d6, 50 MHz). Figura S26. Reverse phase HPLC spectrum of 1f (LASSBio-1463) (acetonitrile:water (60∶40)). Table S1. p38α MAPK inhibitory activity of compounds (1a–c, 1 k) at 10 µM. (ZIP) [file pone.0091660.s001.zip › Figure S20.tiff]

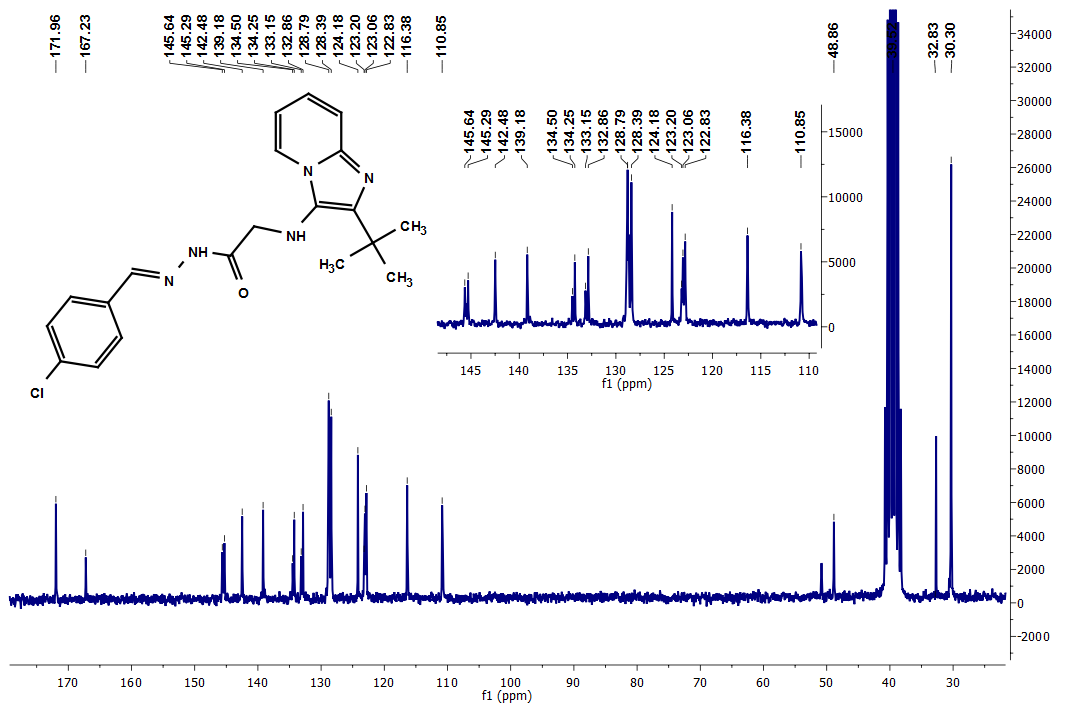

Supplement: File S1 — Figures S1–S26 and Table S1. Figure S1. 1H NMR spectrum of 1a (LASSBio-1507) (DMSO-d6, 200 MHz). Figure S2. 13C NMR spectrum of 1a (LASSBio-1507) (DMSO-d6, 50 MHz). Figure S3. 1H NMR spectrum of 1b (LASSBio-1616) (DMSO-d6, 200 MHz). Figure S4. 1H NMR spectrum of 1b (LASSBio-1616) (CDCl3, 200 MHz). Figure S5. 13C NMR spectrum of 1b (LASSBio-1616) (CDCl3, 50 MHz). Figure S6. 1H NMR spectrum of 1c (LASSBio-1535) (DMSO-d6, 200 MHz). Figure S7. 13C NMR spectrum of 1c (LASSBio-1535) (DMSO-d6, 50 MHz). Figure S8. 1H NMR spectrum of 1d (LASSBio-1695) (DMSO-d6, 200 MHz). Figure S9. 13C NMR spectrum of 1d (LASSBio-1695) (DMSO-d6, 50 MHz). Figure S10. 1H NMR spectrum of 1e (LASSBio-1696) (DMSO-d6, 200 MHz). Figure S11. 13C NMR spectrum of 1e (LASSBio-1696) (DMSO-d6, 50 MHz). Figure S12. 1H NMR spectrum of 1f (LASSBio-1463) (DMSO-d6, 300 MHz). Figure S13. 1H NMR spectrum of 1f (LASSBio-1463) (DMSO-d6, 300 MHz, T∼90°C). Figure S14. 13C NMR spectrum of 1f (LASSBio-1463) (DMSO-d6, 50 MHz). Figure S15. 1H NMR spectrum of 1g (LASSBio-1626) (DMSO-d6, 300 MHz). Figure S16. 1H NMR spectrum of 1g (LASSBio-1626) (DMSO-d6, 300 MHz, T∼90°C). Figure S17. 13C NMR spectrum of 1g (LASSBio-1626) (DMSO-d6, 50 MHz). Figure S18. 1H NMR spectrum of 1h (LASSBio-1697) (DMSO-d6, 200 MHz). Figure S19. 13C NMR spectrum of 1h (LASSBio-1697) (DMSO-d6, 50 MHz). Figure S20. 1H NMR spectrum of 1i (LASSBio-1749) (DMSO-d6, 200 MHz). Figure S21. 13C NMR spectrum of 1i (LASSBio-1749) (DMSO-d6, 50 MHz). Figure S22. 1H NMR spectrum of 1j (LASSBio-1698) (DMSO-d6, 200 MHz). Figure S23. 13C NMR spectrum of 1j (LASSBio-1698) (DMSO-d6, 50 MHz). Figura S24. 1H NMR spectrum of 1k (LASSBio-1615) (DMSO-d6, 200 MHz). Figura S25. 13C NMR spectrum of 1k (LASSBio-1615) (DMSO-d6, 50 MHz). Figura S26. Reverse phase HPLC spectrum of 1f (LASSBio-1463) (acetonitrile:water (60∶40)). Table S1. p38α MAPK inhibitory activity of compounds (1a–c, 1 k) at 10 µM. (ZIP) [file pone.0091660.s001.zip › Figure S21.tiff]

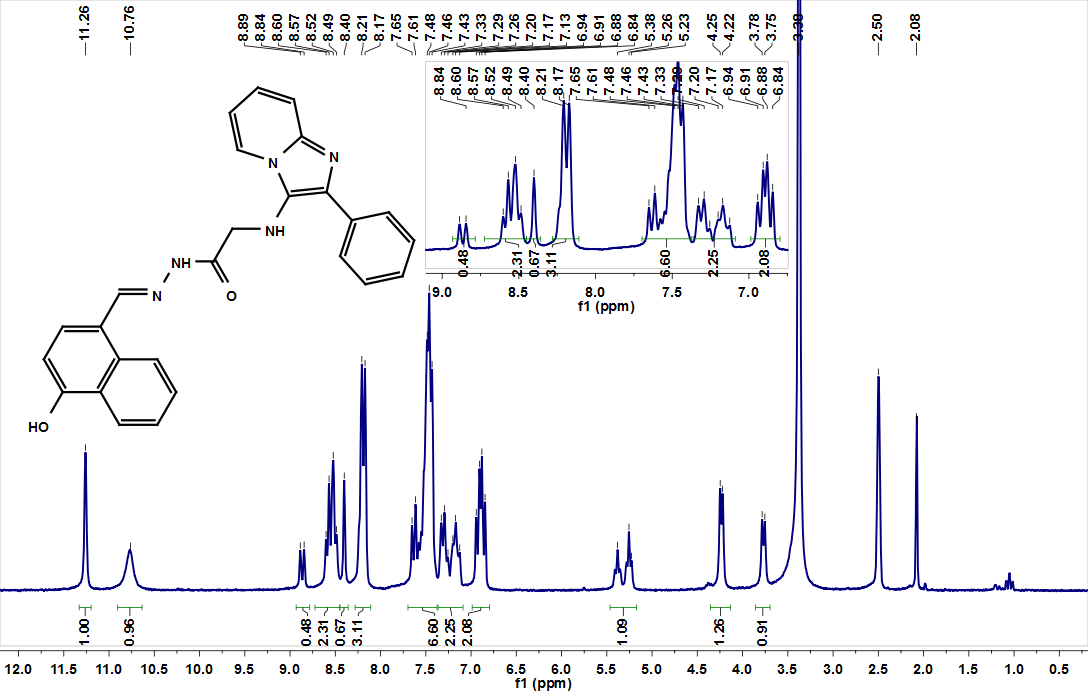

Supplement: File S1 — Figures S1–S26 and Table S1. Figure S1. 1H NMR spectrum of 1a (LASSBio-1507) (DMSO-d6, 200 MHz). Figure S2. 13C NMR spectrum of 1a (LASSBio-1507) (DMSO-d6, 50 MHz). Figure S3. 1H NMR spectrum of 1b (LASSBio-1616) (DMSO-d6, 200 MHz). Figure S4. 1H NMR spectrum of 1b (LASSBio-1616) (CDCl3, 200 MHz). Figure S5. 13C NMR spectrum of 1b (LASSBio-1616) (CDCl3, 50 MHz). Figure S6. 1H NMR spectrum of 1c (LASSBio-1535) (DMSO-d6, 200 MHz). Figure S7. 13C NMR spectrum of 1c (LASSBio-1535) (DMSO-d6, 50 MHz). Figure S8. 1H NMR spectrum of 1d (LASSBio-1695) (DMSO-d6, 200 MHz). Figure S9. 13C NMR spectrum of 1d (LASSBio-1695) (DMSO-d6, 50 MHz). Figure S10. 1H NMR spectrum of 1e (LASSBio-1696) (DMSO-d6, 200 MHz). Figure S11. 13C NMR spectrum of 1e (LASSBio-1696) (DMSO-d6, 50 MHz). Figure S12. 1H NMR spectrum of 1f (LASSBio-1463) (DMSO-d6, 300 MHz). Figure S13. 1H NMR spectrum of 1f (LASSBio-1463) (DMSO-d6, 300 MHz, T∼90°C). Figure S14. 13C NMR spectrum of 1f (LASSBio-1463) (DMSO-d6, 50 MHz). Figure S15. 1H NMR spectrum of 1g (LASSBio-1626) (DMSO-d6, 300 MHz). Figure S16. 1H NMR spectrum of 1g (LASSBio-1626) (DMSO-d6, 300 MHz, T∼90°C). Figure S17. 13C NMR spectrum of 1g (LASSBio-1626) (DMSO-d6, 50 MHz). Figure S18. 1H NMR spectrum of 1h (LASSBio-1697) (DMSO-d6, 200 MHz). Figure S19. 13C NMR spectrum of 1h (LASSBio-1697) (DMSO-d6, 50 MHz). Figure S20. 1H NMR spectrum of 1i (LASSBio-1749) (DMSO-d6, 200 MHz). Figure S21. 13C NMR spectrum of 1i (LASSBio-1749) (DMSO-d6, 50 MHz). Figure S22. 1H NMR spectrum of 1j (LASSBio-1698) (DMSO-d6, 200 MHz). Figure S23. 13C NMR spectrum of 1j (LASSBio-1698) (DMSO-d6, 50 MHz). Figura S24. 1H NMR spectrum of 1k (LASSBio-1615) (DMSO-d6, 200 MHz). Figura S25. 13C NMR spectrum of 1k (LASSBio-1615) (DMSO-d6, 50 MHz). Figura S26. Reverse phase HPLC spectrum of 1f (LASSBio-1463) (acetonitrile:water (60∶40)). Table S1. p38α MAPK inhibitory activity of compounds (1a–c, 1 k) at 10 µM. (ZIP) [file pone.0091660.s001.zip › Figure S22.tiff]

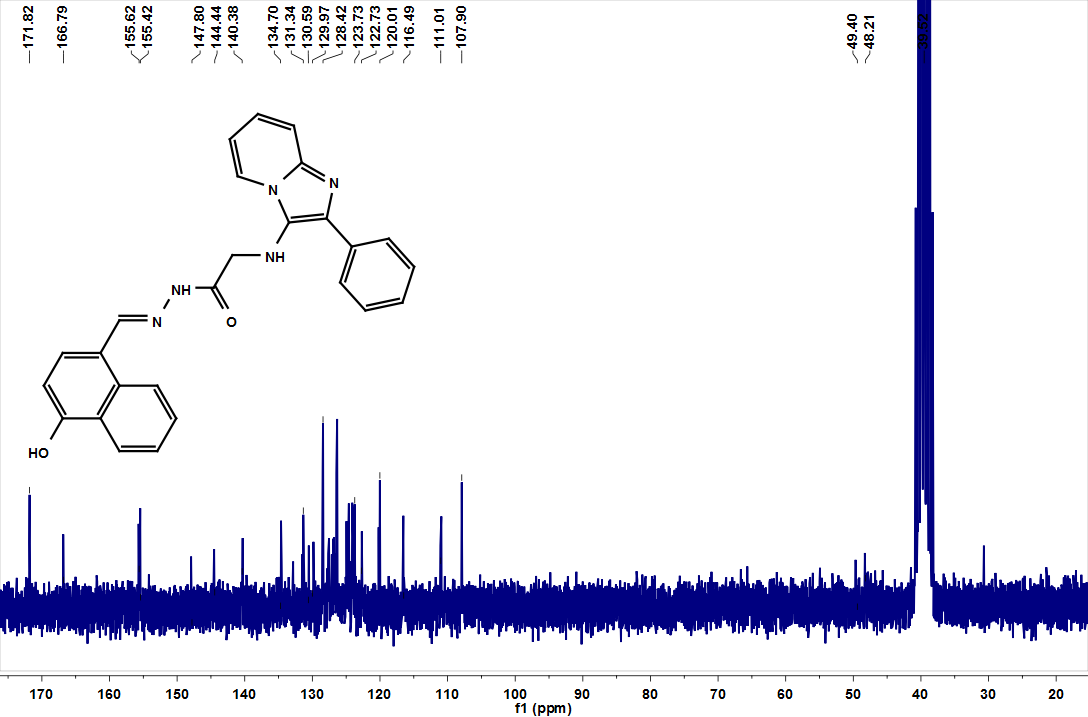

Supplement: File S1 — Figures S1–S26 and Table S1. Figure S1. 1H NMR spectrum of 1a (LASSBio-1507) (DMSO-d6, 200 MHz). Figure S2. 13C NMR spectrum of 1a (LASSBio-1507) (DMSO-d6, 50 MHz). Figure S3. 1H NMR spectrum of 1b (LASSBio-1616) (DMSO-d6, 200 MHz). Figure S4. 1H NMR spectrum of 1b (LASSBio-1616) (CDCl3, 200 MHz). Figure S5. 13C NMR spectrum of 1b (LASSBio-1616) (CDCl3, 50 MHz). Figure S6. 1H NMR spectrum of 1c (LASSBio-1535) (DMSO-d6, 200 MHz). Figure S7. 13C NMR spectrum of 1c (LASSBio-1535) (DMSO-d6, 50 MHz). Figure S8. 1H NMR spectrum of 1d (LASSBio-1695) (DMSO-d6, 200 MHz). Figure S9. 13C NMR spectrum of 1d (LASSBio-1695) (DMSO-d6, 50 MHz). Figure S10. 1H NMR spectrum of 1e (LASSBio-1696) (DMSO-d6, 200 MHz). Figure S11. 13C NMR spectrum of 1e (LASSBio-1696) (DMSO-d6, 50 MHz). Figure S12. 1H NMR spectrum of 1f (LASSBio-1463) (DMSO-d6, 300 MHz). Figure S13. 1H NMR spectrum of 1f (LASSBio-1463) (DMSO-d6, 300 MHz, T∼90°C). Figure S14. 13C NMR spectrum of 1f (LASSBio-1463) (DMSO-d6, 50 MHz). Figure S15. 1H NMR spectrum of 1g (LASSBio-1626) (DMSO-d6, 300 MHz). Figure S16. 1H NMR spectrum of 1g (LASSBio-1626) (DMSO-d6, 300 MHz, T∼90°C). Figure S17. 13C NMR spectrum of 1g (LASSBio-1626) (DMSO-d6, 50 MHz). Figure S18. 1H NMR spectrum of 1h (LASSBio-1697) (DMSO-d6, 200 MHz). Figure S19. 13C NMR spectrum of 1h (LASSBio-1697) (DMSO-d6, 50 MHz). Figure S20. 1H NMR spectrum of 1i (LASSBio-1749) (DMSO-d6, 200 MHz). Figure S21. 13C NMR spectrum of 1i (LASSBio-1749) (DMSO-d6, 50 MHz). Figure S22. 1H NMR spectrum of 1j (LASSBio-1698) (DMSO-d6, 200 MHz). Figure S23. 13C NMR spectrum of 1j (LASSBio-1698) (DMSO-d6, 50 MHz). Figura S24. 1H NMR spectrum of 1k (LASSBio-1615) (DMSO-d6, 200 MHz). Figura S25. 13C NMR spectrum of 1k (LASSBio-1615) (DMSO-d6, 50 MHz). Figura S26. Reverse phase HPLC spectrum of 1f (LASSBio-1463) (acetonitrile:water (60∶40)). Table S1. p38α MAPK inhibitory activity of compounds (1a–c, 1 k) at 10 µM. (ZIP) [file pone.0091660.s001.zip › Figure S23.tiff]

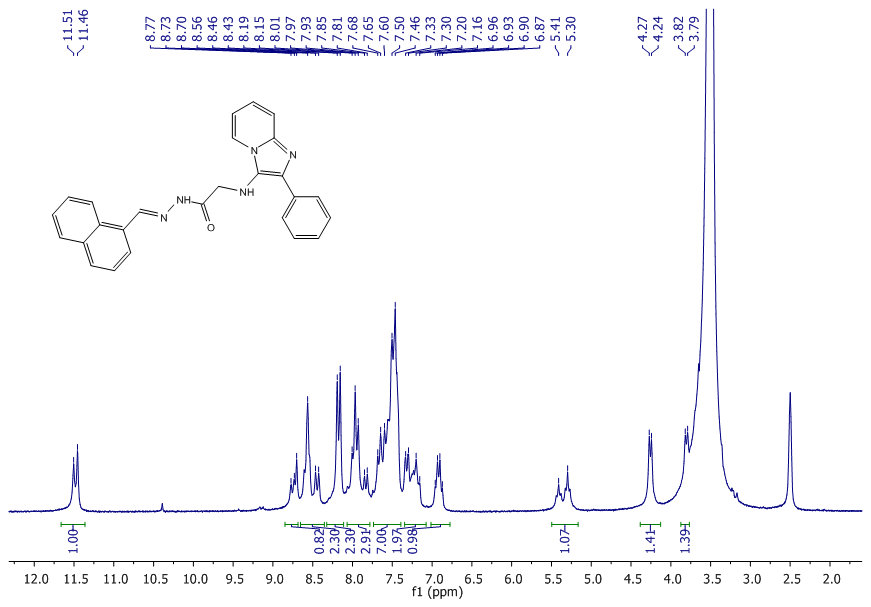

Supplement: File S1 — Figures S1–S26 and Table S1. Figure S1. 1H NMR spectrum of 1a (LASSBio-1507) (DMSO-d6, 200 MHz). Figure S2. 13C NMR spectrum of 1a (LASSBio-1507) (DMSO-d6, 50 MHz). Figure S3. 1H NMR spectrum of 1b (LASSBio-1616) (DMSO-d6, 200 MHz). Figure S4. 1H NMR spectrum of 1b (LASSBio-1616) (CDCl3, 200 MHz). Figure S5. 13C NMR spectrum of 1b (LASSBio-1616) (CDCl3, 50 MHz). Figure S6. 1H NMR spectrum of 1c (LASSBio-1535) (DMSO-d6, 200 MHz). Figure S7. 13C NMR spectrum of 1c (LASSBio-1535) (DMSO-d6, 50 MHz). Figure S8. 1H NMR spectrum of 1d (LASSBio-1695) (DMSO-d6, 200 MHz). Figure S9. 13C NMR spectrum of 1d (LASSBio-1695) (DMSO-d6, 50 MHz). Figure S10. 1H NMR spectrum of 1e (LASSBio-1696) (DMSO-d6, 200 MHz). Figure S11. 13C NMR spectrum of 1e (LASSBio-1696) (DMSO-d6, 50 MHz). Figure S12. 1H NMR spectrum of 1f (LASSBio-1463) (DMSO-d6, 300 MHz). Figure S13. 1H NMR spectrum of 1f (LASSBio-1463) (DMSO-d6, 300 MHz, T∼90°C). Figure S14. 13C NMR spectrum of 1f (LASSBio-1463) (DMSO-d6, 50 MHz). Figure S15. 1H NMR spectrum of 1g (LASSBio-1626) (DMSO-d6, 300 MHz). Figure S16. 1H NMR spectrum of 1g (LASSBio-1626) (DMSO-d6, 300 MHz, T∼90°C). Figure S17. 13C NMR spectrum of 1g (LASSBio-1626) (DMSO-d6, 50 MHz). Figure S18. 1H NMR spectrum of 1h (LASSBio-1697) (DMSO-d6, 200 MHz). Figure S19. 13C NMR spectrum of 1h (LASSBio-1697) (DMSO-d6, 50 MHz). Figure S20. 1H NMR spectrum of 1i (LASSBio-1749) (DMSO-d6, 200 MHz). Figure S21. 13C NMR spectrum of 1i (LASSBio-1749) (DMSO-d6, 50 MHz). Figure S22. 1H NMR spectrum of 1j (LASSBio-1698) (DMSO-d6, 200 MHz). Figure S23. 13C NMR spectrum of 1j (LASSBio-1698) (DMSO-d6, 50 MHz). Figura S24. 1H NMR spectrum of 1k (LASSBio-1615) (DMSO-d6, 200 MHz). Figura S25. 13C NMR spectrum of 1k (LASSBio-1615) (DMSO-d6, 50 MHz). Figura S26. Reverse phase HPLC spectrum of 1f (LASSBio-1463) (acetonitrile:water (60∶40)). Table S1. p38α MAPK inhibitory activity of compounds (1a–c, 1 k) at 10 µM. (ZIP) [file pone.0091660.s001.zip › Figure S24.tif]

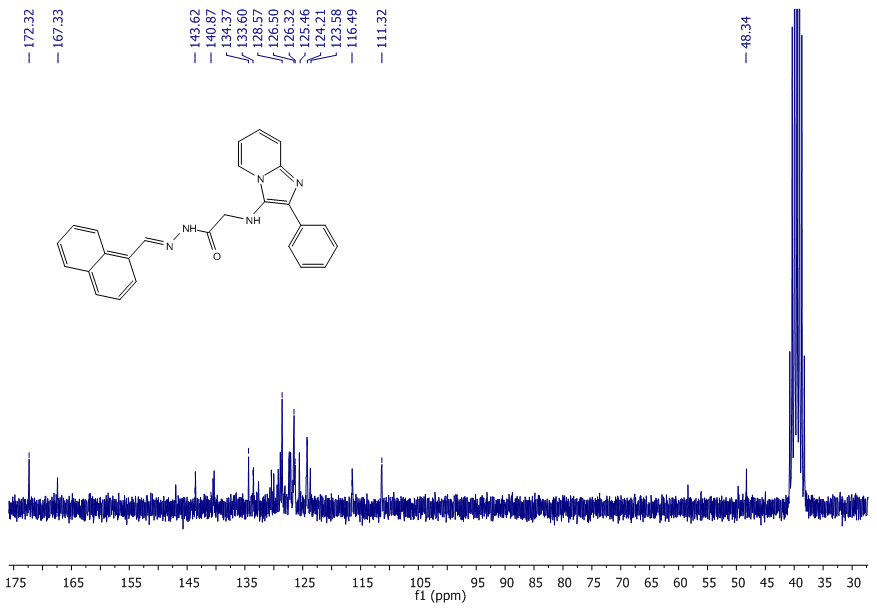

Supplement: File S1 — Figures S1–S26 and Table S1. Figure S1. 1H NMR spectrum of 1a (LASSBio-1507) (DMSO-d6, 200 MHz). Figure S2. 13C NMR spectrum of 1a (LASSBio-1507) (DMSO-d6, 50 MHz). Figure S3. 1H NMR spectrum of 1b (LASSBio-1616) (DMSO-d6, 200 MHz). Figure S4. 1H NMR spectrum of 1b (LASSBio-1616) (CDCl3, 200 MHz). Figure S5. 13C NMR spectrum of 1b (LASSBio-1616) (CDCl3, 50 MHz). Figure S6. 1H NMR spectrum of 1c (LASSBio-1535) (DMSO-d6, 200 MHz). Figure S7. 13C NMR spectrum of 1c (LASSBio-1535) (DMSO-d6, 50 MHz). Figure S8. 1H NMR spectrum of 1d (LASSBio-1695) (DMSO-d6, 200 MHz). Figure S9. 13C NMR spectrum of 1d (LASSBio-1695) (DMSO-d6, 50 MHz). Figure S10. 1H NMR spectrum of 1e (LASSBio-1696) (DMSO-d6, 200 MHz). Figure S11. 13C NMR spectrum of 1e (LASSBio-1696) (DMSO-d6, 50 MHz). Figure S12. 1H NMR spectrum of 1f (LASSBio-1463) (DMSO-d6, 300 MHz). Figure S13. 1H NMR spectrum of 1f (LASSBio-1463) (DMSO-d6, 300 MHz, T∼90°C). Figure S14. 13C NMR spectrum of 1f (LASSBio-1463) (DMSO-d6, 50 MHz). Figure S15. 1H NMR spectrum of 1g (LASSBio-1626) (DMSO-d6, 300 MHz). Figure S16. 1H NMR spectrum of 1g (LASSBio-1626) (DMSO-d6, 300 MHz, T∼90°C). Figure S17. 13C NMR spectrum of 1g (LASSBio-1626) (DMSO-d6, 50 MHz). Figure S18. 1H NMR spectrum of 1h (LASSBio-1697) (DMSO-d6, 200 MHz). Figure S19. 13C NMR spectrum of 1h (LASSBio-1697) (DMSO-d6, 50 MHz). Figure S20. 1H NMR spectrum of 1i (LASSBio-1749) (DMSO-d6, 200 MHz). Figure S21. 13C NMR spectrum of 1i (LASSBio-1749) (DMSO-d6, 50 MHz). Figure S22. 1H NMR spectrum of 1j (LASSBio-1698) (DMSO-d6, 200 MHz). Figure S23. 13C NMR spectrum of 1j (LASSBio-1698) (DMSO-d6, 50 MHz). Figura S24. 1H NMR spectrum of 1k (LASSBio-1615) (DMSO-d6, 200 MHz). Figura S25. 13C NMR spectrum of 1k (LASSBio-1615) (DMSO-d6, 50 MHz). Figura S26. Reverse phase HPLC spectrum of 1f (LASSBio-1463) (acetonitrile:water (60∶40)). Table S1. p38α MAPK inhibitory activity of compounds (1a–c, 1 k) at 10 µM. (ZIP) [file pone.0091660.s001.zip › Figure S25.tif]

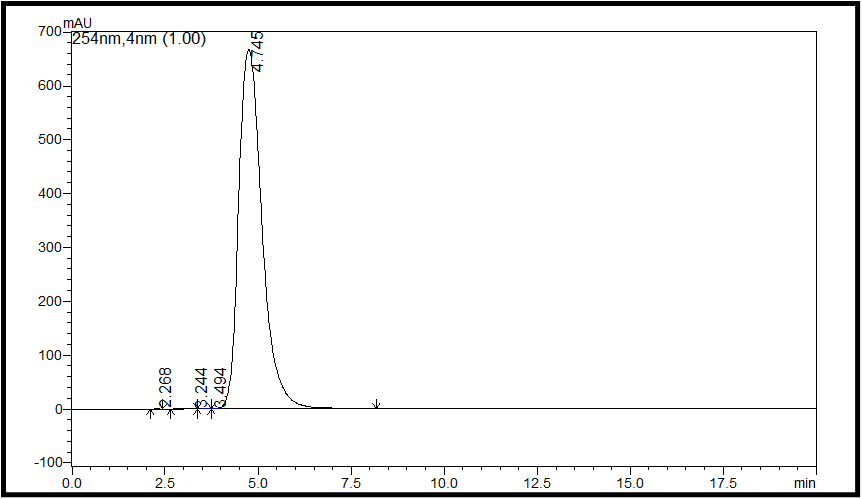

Supplement: File S1 — Figures S1–S26 and Table S1. Figure S1. 1H NMR spectrum of 1a (LASSBio-1507) (DMSO-d6, 200 MHz). Figure S2. 13C NMR spectrum of 1a (LASSBio-1507) (DMSO-d6, 50 MHz). Figure S3. 1H NMR spectrum of 1b (LASSBio-1616) (DMSO-d6, 200 MHz). Figure S4. 1H NMR spectrum of 1b (LASSBio-1616) (CDCl3, 200 MHz). Figure S5. 13C NMR spectrum of 1b (LASSBio-1616) (CDCl3, 50 MHz). Figure S6. 1H NMR spectrum of 1c (LASSBio-1535) (DMSO-d6, 200 MHz). Figure S7. 13C NMR spectrum of 1c (LASSBio-1535) (DMSO-d6, 50 MHz). Figure S8. 1H NMR spectrum of 1d (LASSBio-1695) (DMSO-d6, 200 MHz). Figure S9. 13C NMR spectrum of 1d (LASSBio-1695) (DMSO-d6, 50 MHz). Figure S10. 1H NMR spectrum of 1e (LASSBio-1696) (DMSO-d6, 200 MHz). Figure S11. 13C NMR spectrum of 1e (LASSBio-1696) (DMSO-d6, 50 MHz). Figure S12. 1H NMR spectrum of 1f (LASSBio-1463) (DMSO-d6, 300 MHz). Figure S13. 1H NMR spectrum of 1f (LASSBio-1463) (DMSO-d6, 300 MHz, T∼90°C). Figure S14. 13C NMR spectrum of 1f (LASSBio-1463) (DMSO-d6, 50 MHz). Figure S15. 1H NMR spectrum of 1g (LASSBio-1626) (DMSO-d6, 300 MHz). Figure S16. 1H NMR spectrum of 1g (LASSBio-1626) (DMSO-d6, 300 MHz, T∼90°C). Figure S17. 13C NMR spectrum of 1g (LASSBio-1626) (DMSO-d6, 50 MHz). Figure S18. 1H NMR spectrum of 1h (LASSBio-1697) (DMSO-d6, 200 MHz). Figure S19. 13C NMR spectrum of 1h (LASSBio-1697) (DMSO-d6, 50 MHz). Figure S20. 1H NMR spectrum of 1i (LASSBio-1749) (DMSO-d6, 200 MHz). Figure S21. 13C NMR spectrum of 1i (LASSBio-1749) (DMSO-d6, 50 MHz). Figure S22. 1H NMR spectrum of 1j (LASSBio-1698) (DMSO-d6, 200 MHz). Figure S23. 13C NMR spectrum of 1j (LASSBio-1698) (DMSO-d6, 50 MHz). Figura S24. 1H NMR spectrum of 1k (LASSBio-1615) (DMSO-d6, 200 MHz). Figura S25. 13C NMR spectrum of 1k (LASSBio-1615) (DMSO-d6, 50 MHz). Figura S26. Reverse phase HPLC spectrum of 1f (LASSBio-1463) (acetonitrile:water (60∶40)). Table S1. p38α MAPK inhibitory activity of compounds (1a–c, 1 k) at 10 µM. (ZIP) [file pone.0091660.s001.zip › Figure S26.tif]
