# Supplementary material for: Novel Potent Imidazo[1,2-a]pyridine-N-Glycinyl-Hydrazone Inhibitors of TNF-α Production: In Vitro and In Vivo Studies
Source: PLoS One. 2014 Mar 14;9(3):e91660. doi: 10.1371/journal.pone.0091660 (PMC3954757; doi:10.1371/journal.pone.0091660)
Supplement: File S1 — Figures S1–S26 and Table S1. Figure S1. 1H NMR spectrum of 1a (LASSBio-1507) (DMSO-d6, 200 MHz). Figure S2. 13C NMR spectrum of 1a (LASSBio-1507) (DMSO-d6, 50 MHz). Figure S3. 1H NMR spectrum of 1b (LASSBio-1616) (DMSO-d6, 200 MHz). Figure S4. 1H NMR spectrum of 1b (LASSBio-1616) (CDCl3, 200 MHz). Figure S5. 13C NMR spectrum of 1b (LASSBio-1616) (CDCl3, 50 MHz). Figure S6. 1H NMR spectrum of 1c (LASSBio-1535) (DMSO-d6, 200 MHz). Figure S7. 13C NMR spectrum of 1c (LASSBio-1535) (DMSO-d6, 50 MHz). Figure S8. 1H NMR spectrum of 1d (LASSBio-1695) (DMSO-d6, 200 MHz). Figure S9. 13C NMR spectrum of 1d (LASSBio-1695) (DMSO-d6, 50 MHz). Figure S10. 1H NMR spectrum of 1e (LASSBio-1696) (DMSO-d6, 200 MHz). Figure S11. 13C NMR spectrum of 1e (LASSBio-1696) (DMSO-d6, 50 MHz). Figure S12. 1H NMR spectrum of 1f (LASSBio-1463) (DMSO-d6, 300 MHz). Figure S13. 1H NMR spectrum of 1f (LASSBio-1463) (DMSO-d6, 300 MHz, T∼90°C). Figure S14. 13C NMR spectrum of 1f (LASSBio-1463) (DMSO-d6, 50 MHz). Figure S15. 1H NMR spectrum of 1g (LASSBio-1626) (DMSO-d6, 300 MHz). Figure S16. 1H NMR spectrum of 1g (LASSBio-1626) (DMSO-d6, 300 MHz, T∼90°C). Figure S17. 13C NMR spectrum of 1g (LASSBio-1626) (DMSO-d6, 50 MHz). Figure S18. 1H NMR spectrum of 1h (LASSBio-1697) (DMSO-d6, 200 MHz). Figure S19. 13C NMR spectrum of 1h (LASSBio-1697) (DMSO-d6, 50 MHz). Figure S20. 1H NMR spectrum of 1i (LASSBio-1749) (DMSO-d6, 200 MHz). Figure S21. 13C NMR spectrum of 1i (LASSBio-1749) (DMSO-d6, 50 MHz). Figure S22. 1H NMR spectrum of 1j (LASSBio-1698) (DMSO-d6, 200 MHz). Figure S23. 13C NMR spectrum of 1j (LASSBio-1698) (DMSO-d6, 50 MHz). Figura S24. 1H NMR spectrum of 1k (LASSBio-1615) (DMSO-d6, 200 MHz). Figura S25. 13C NMR spectrum of 1k (LASSBio-1615) (DMSO-d6, 50 MHz). Figura S26. Reverse phase HPLC spectrum of 1f (LASSBio-1463) (acetonitrile:water (60∶40)). Table S1. p38α MAPK inhibitory activity of compounds (1a–c, 1 k) at 10 µM. (ZIP) [file pone.0091660.s001.zip › Table S1.doc]

**Table S1. p38α MAPK inhibitory activity of compounds (1a-c, 1k) at 10 μM.**

| **Compound** | **% inhibition of p38α MAPK*** |
| --- | --- |
| **1a (LASSBio-1507)** | **11.81** |
| **1b (LASSBio-1616)** | **6.50** |
| **1c (LASSBio-1535)** | **22.56** |
| **1k (LASSBio-1615)** | **17.20** |

***This assay was performed as described by Goettert MR, Graeser, Laufer SA (2010) Optimization of a nonradioactive immunosorbent assay for p38 alpha mitogen-activated protein kinase activity. Anal Biochem 406: 233–234.**
